# Cancer Reduces Transcriptome Specialization

**DOI:** 10.1371/journal.pone.0010398

**Published:** 2010-05-03

**Authors:** Octavio Martínez, M. Humberto Reyes-Valdés, Luis Herrera-Estrella

**Affiliations:** 1 Laboratorio Nacional de Genómica para la Biodiversidad, Centro de Investigación y de Estudios Avanzados del Instituto Politécnico Nacional, Irapuato, Guanajuato, México; 2 Department of Plant Breeding, Universidad Autónoma Agraria Antonio Narro, Saltillo, Coahuila, México; Michigan State University, United States of America

## Abstract

A central goal of cancer biology is to understand how cells from this family of genetic diseases undergo specific morphological and physiological changes and regress to a de-regulated state of the cell cycle. The fact that tumors are unable to perform most of the specific functions of the original tissue led us to hypothesize that the degree of specialization of the transcriptome of cancerous tissues must be less than their normal counterparts. With the aid of information theory tools, we analyzed four datasets derived from transcriptomes of normal and tumor tissues to quantitatively test the hypothesis that cancer reduces transcriptome specialization. Here, we show that the transcriptional specialization of a tumor is significantly less than the corresponding normal tissue and comparable with the specialization of dedifferentiated embryonic stem cells. Furthermore, we demonstrate that the drop in specialization in cancerous tissues is largely due to a decrease in expression of genes that are highly specific to the normal organ. This approach gives us a better understanding of carcinogenesis and offers new tools for the identification of genes that are highly influential in cancer progression.

## Introduction

Cancer is a complex family of acquired genetic diseases in which a single cell clone and its progeny accumulate heritable changes that cause a malignant phenotype of deregulated cell growth and differentiation [1]. Numerous studies have been performed to better understand the alterations that occur in the transcription profile during the progression of cancer [2]. These experiments have been carried out by directly counting the tags of expressed genes using serial analysis of gene expression (SAGE) [3], expressed sequence tags (ESTs) [4], and other counting strategies, or by indirectly measuring the levels of transcription using DNA microarrays [5]. In many cases, these experiments have detected genes that are preferentially expressed in a cancer tumor and can serve as molecular markers of malignancy. In addition, they can also detect significant alterations in the transcription level of sets of genes that participate in complex signaling networks. Changes in these networks represent distortions of the pathways that regulate the physiology of normal cells [6].

Cancer cells lose the ability to perform the normal functions of the original tissue, and at the same time, gain characteristics that allow them to survive as an independent and frequently invasive tumor. As cell lines evolve from a normal to a cancerous state, mutations drive an increase in genetic diversity [7]. This process occurs in parallel with the selection of phenotypes and genotypes that permit the pre-cancerous cells to thrive in their microenvironment [8]. Tumor cells often lack the differentiation that is present in the normal tissue that they originate from. Since the mid-19^th^ century, this fact has lead pathologists to suggest that tumors arise from embryo-like cells [9]. Given that cancer must arise from a cell that has the potential to divide, two nonexclusive hypotheses of the cellular origin of tumors have historically been proposed. The first hypothesis states that malignancy arises from stem cells due to maturation arrest; the second states that cancer arises from the dedifferentiation of mature cells [10]. More recently, however, the concept of “cancer stem cells”, or rare cells with a limitless potential for self-renewal, has gained acceptance as a subpopulation of cells that drives tumorigenesis. This hypothesis is based on findings that have shown that in some cases, only a subset of the cells within a tumor have unlimited proliferative potential [11]. However, this hypothesis remains controversial, since the growth of certain malignant tumors is driven by a substantial percentage of tumor cells that are not cancer stem cells (greater than 10%) [12]. Regardless, there is clear evidence that the undifferentiated phenotype of many tumor cells resembles the phenotype of undifferentiated normal cells, such as embryonic stem cells. Moreover, the study of gene expression in cancer tumors has revealed that poorly differentiated tumors show preferential overexpression of genes normally enriched in embryonic stem cells, supporting the possibility that these genes contribute to the stem cell-like phenotypes shown by many tumors [13].

Previously, we described the development of indexes based on Shannon's information theory to measure transcriptome diversity, specialization, and gene specificity of normal organs and tissues [14]. In that study, we obtained an index of gene specificity, *S_i_*, which has a value of zero for genes that are equally expressed in all tissues and has a defined maximum value when a gene is expressed in only one tissue. Transcriptome specialization, *δ_j_*, therefore, is defined as the average gene specificity expressed in the transcriptome (see Materials and Methods). In general, a tissue is more specialized if specific genes are highly expressed in it. We also demonstrated that human organs have a particular degree of diversity and specialization that is related to their functionality. In this study, we applied information theory tools to compare the transcriptome diversity and specialization of cancerous tumors vs. their normal counterparts. We show that the specialization of cancer tissues generally diminishes when compared with their normal counterparts, which is mainly due to the decrease in expression of highly specific genes.

## Results

We hypothesized that the morphological and functional changes that occur during cancer progression would lead to substantial changes in the cancer transcriptome, including a reduction in specialization, when compared to that of analogous normal tissues. To test this hypothesis in a broad framework, we selected three collections of gene tags and one microarray experiment. Datasets ***A*** and ***B*** are selected collections of cDNA libraries from the “Cancer Genome Anatomy Project” [15] for human and mouse tissues, respectively. Dataset ***C*** consists of SAGE libraries from normal human and tumor tissues obtained from the “Human Transcriptome Map” project [16] and dataset ***D*** is a microarray study of human tissues in normal and pre-cancerous states that were paired by patient [17]. Datasets ***A*** and ***B*** incorporate five embryonic stem cell (ESC) and one hematopoietic stem cell (HSC) libraries and were included in the analysis based on their degree of dedifferentiation. These datasets were subjected to the analysis of information properties of the transcriptome as previously described [14]. In the counting tags datasets, we assessed the statistical significance of the differences in specialization. In each case, we obtained the specificity (*S_i_*) and Target Specificity (*TS_ij_*) for the genes studied in the datasets, which allowed for the selection of putative overexpressed genes in cancer or normal tissues as well as the discrimination of genes preferentially expressed in a given condition.

### Evaluating transcriptome specialization in normal and cancerous tissues

The analysis of datasets ***A*** and ***B*** for estimating the transcriptome diversity (*H_j,_*) and specialization (*δ_j_*) indexes were done at three levels of cDNA library grouping with the following designation: individual cDNA libraries were defined as “ungrouped”; assembled cDNA libraries originating from the same kind of organ and tissue state (normal or cancer) were defined as “grouped”; and a further clustering of the cDNA libraries that only considered the tissue state and not the organ of origin were defined as “complete grouping” (see Materials and Methods). *H_j_* measured the variability of the distributions of transcripts and *δ_j_* assessed the average specificity of the genes expressed in the transcriptome. Visualization of the positions of the transcriptomes in the (*H_j_*, *δ_j_*) coordinates allowed us to effectively evaluate the relative differences in these significant parameters. Results for the ungrouped analysis, dataset D analysis, tables of confidence intervals for the relevant parameters, and dissection of the differences in transcriptome specialization are discussed in Supporting Text S1.

**Figure 1:**
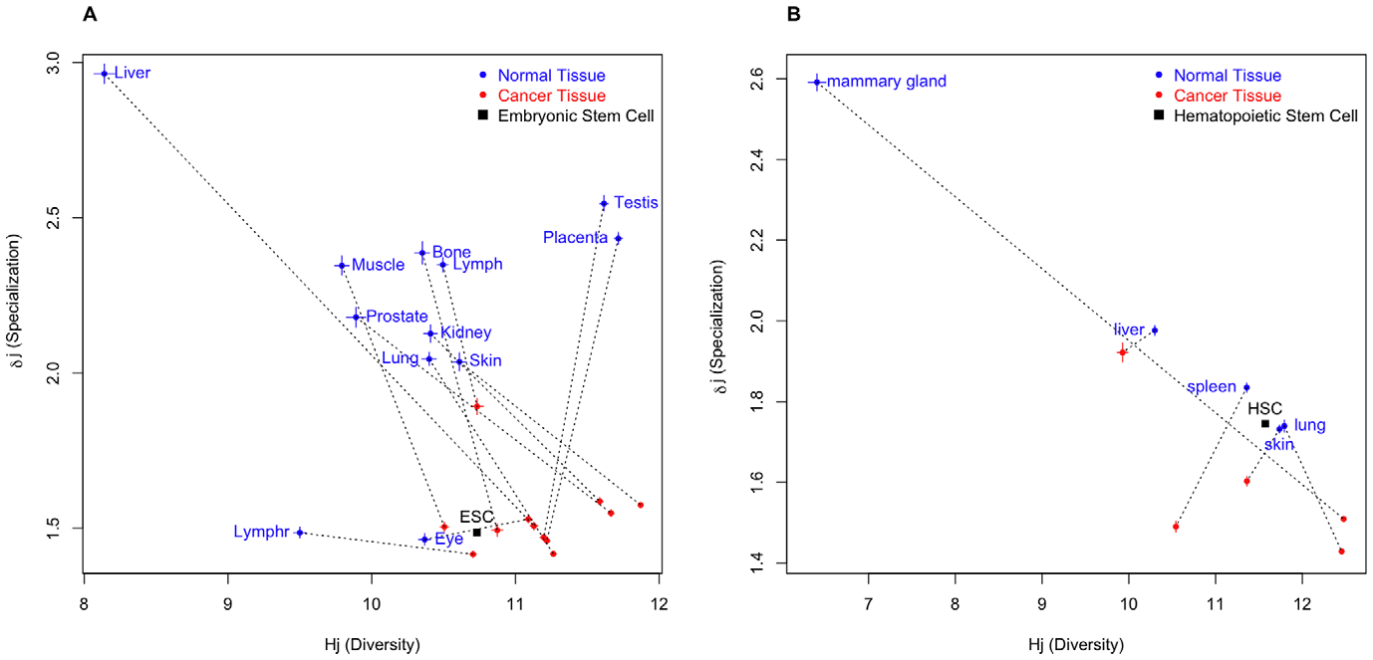
Scatter plot of *H_j_* (Diversity) and *δ_j_* (Specialization) in transcriptomes of normal tissues (blue), cancerous tissues (red), and stem cells (black). Comparable data sets are linked by a discontinuous line. ***A*** - Human data from 53 libraries of 13 distinct tissues with a total of 671,197 tags for 28,087 genes; grouped analyses. ***B*** - Mouse data from 29 libraries of 5 distinct tissues and with a total of 541,453 expressed tags for 25,044 distinct genes; grouped analyses. Data for ***A*** and ***B*** are from the “Cancer Genome Anatomy Project” (http://cgap.nci.nih.gov/). Approximate 95% confidence intervals for diversity and specialization are plotted as continuous colored lines. See Supporting Text S1 as well as Figure S1, Figure S2, Figure S3, Figure S4, Figure S5 and Figure S6 that illustrate individual comparisons and details.

Figure 1 presents scatter plots for the levels of transcriptome diversity, *H_j_*, and specialization, *δ_j_*, at the grouped level for datasets ***A*** and ***B***. When comparing 12 pairs of human analogous tissues, 11 cancerous tissues had significantly less specialization than their normal counterparts, with eye cancer being the only tissue that had an estimated specialization that was greater that its normal counterpart (Figure 1A, Table S4, and Supporting Text S1). However, after further analysis, we concluded that the eye tissue sample is most likely distorted due to the smaller sample size of the normal eye library (10,679 tags) compared to the cancerous eye library (42,029 tags) (see Supporting Text S1). This likely prevented the correct estimation of eye-specific genes in the normal library. All changes in specialization of the transcriptomes are statistically significant (Table S4 and Table S5; P<0.01). Figure 1A also shows that transcriptome diversity, measured by *H_j_*, increased in the cancerous states of all tissues, with the exception of testis and placenta. The increase in *H_j_* indicates a more even distribution of the transcription levels of expressed genes, which is most likely due to a decrease in the expression of genes prevalent in the normal tissues. As shown in Figure 1A, we observed that the specialization of the grouped ESC libraries is at the same level as the majority of the cancer tissues. This is in agreement with the low morphological specialization of ESCs.

In order to assess the drop in specialization of the cancerous tissues, we compared the average specificity of genes that were over-expressed in normal tissues to that in cancerous tissues. In general, we found that there was a significantly greater average specificity of genes over-expressed in normal tissues, suggesting that the decrease in specialization was due to the reduction or elimination of the expression of highly specialized genes in normal tissues during carcinogenesis (see Table S11 and Figure S11). We also analyzed the ten most influential genes that caused the reduction in specialization in all eleven tissues of dataset ***A.*** For each tissue, we found examples of organ-specific genes that were switched off in the corresponding cancer tissue, including Chondroadherin (*CHAD)* in bone, Uromodulin (*UMOD)* in kidney, the acid phosphatase prostate specific (*ACPP)* gene in the prostate, and a gene for the spermatogenesis-associated protein in the testis (Table S12).

To confirm our hypothesis that specialization decreases in cancerous tissues, we examined a completely independent model of mouse tissues (dataset ***B***). In this analysis, all of the normal tissues showed significantly greater specialization than the corresponding cancerous tissues (Figure 1B, Table S6 and Table S7; P<0.01). Dataset ***B*** also included a library of HSCs obtained from bone marrow. These cells showed a level of specialization comparable with normal lung and skin even when undifferentiated (Figure 1B). In four of the five organs studied in dataset ***B***, the average specificity of the genes that were over-expressed in normal tissues was significantly greater than the corresponding value for cancerous tissues, with the exception of the mammary gland (Table S11). However, genes related to milk production, which were within the most influential genes of the mammary gland and have a high specificity of expression in this tissue (Table S13 and Table S14), showed an extreme drop in expression in the cancerous tissue. These results explain the general drop in specialization seen in the mammary gland. In addition, the scatter plots of gene frequency change between normal and cancerous tissues vs. specialization showed a prevalence of highly specific, over-expressed genes in normal tissues from the five organs studied (Figure S12). We conclude that highly specific genes that have diminished expression in the cancerous tissues drive the drop in specialization, similar to that seen in dataset **A**.

**Figure 2:**
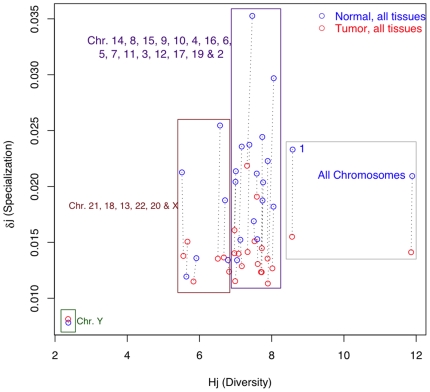
Scatter plot of *H_j_* (Diversity) and *δ_j_* (Specialization) in transcriptomes of normal (blue) and tumor (red) tissues in dataset *C*. Human expression data are from the “Human Transcriptome Map” project (http://bioinfo.amc.uva.nl/HTMseq/controller), datasets “All tissues normal” and “All tissues tumor”. Data consist of 18,609,073 tags for a total of 62,916 loci by chromosome. See Figure S7, Figure S8, Figure S9 and Figure S10 that amplify the boxes of this figure presenting the 95% confidence intervals for the estimates.

The data from the “Human Transcriptome Map” (dataset ***C***) consist of a collection of SAGE gene tags that belong to heterogeneous normal and tumor tissues that are grouped by chromosome. We disregarded the obvious differences in transcription profiles between distinct organs and only tested the hypothesis that specialization diminishes in the tumor transcriptomes. It is worth noting that in contrast to the analyses of datasets ***A*** and ***B***, where gene specificity was estimated for the combination of tissue and condition, in dataset C the specificity is estimated only with regard to the state of the tissue (normal vs. tumor) and disregards the tissue of origin. Therefore, gene specificity in this dataset only refers to normal or tumor tissues and implies that a much lower estimated specialization would be observed. A large and significant change in transcriptome specialization between the normal and tumor tissues was seen for all chromosomes (Figure 2, Figure S7, Figure S8, Figure S9 Figure S10, Table S9, and Supporting Text S1), with the exception of chromosome Y, for which the difference is not significant. Most of the differences that are significant (23 out of 24) are in the expected direction and have less specialization in the tumor transcriptomes. One exception to this was chromosome 18, for which the change in specialization is in the opposite direction (see Supporting Text S1). However, the analysis of all loci together (Figure 2 and Table S9) strongly supports the hypothesis that cancer reduces specialization of the tissues.

In our previous study, we showed that the estimated rank of variation of diversity and specialization in the human transcriptome is much smaller when using microarrays than when counting gene tags [14]. This is due to the relatively narrower dynamic range of microarrays compared with tag counting strategies [18], which distorts both high and low expressed genes. Despite these shortcomings, the analysis of normal (TDLUs) and precancerous (HELUs) tissues paired by patient (dataset ***D***) showed a large drop in specialization in precancerous tissues in seven of the eight cases studied (Figure S13).

### Genes detected only in cancer

The information theory approach for studying the transcriptome has the advantage of allowing an estimation of the degree of global gene specificity, *S_i_*, of each gene studied, as well as its target specificity, *TS_ij_*, a parameter that measures the specificity of a given gene for a selected transcriptome (see Mathematical Addendum in Supporting Text S1). These tools permit the easy selection of genes that are preferentially expressed in cancer tissues and therefore have the potential to serve as molecular markers of malignancy. In addition, these indexes may aid in identifying genes that are specific to a particular kind of cancer or genes that are not significantly altered during the development of cancer and therefore can serve as marker controls when measuring genes of varying expression. It is important to note that when a gene in a particular dataset is detected in only cancer tissues, it cannot be inferred to be exclusively in cancer, since it could also be present in normal tissues at undetectable levels. However, genes with high levels of expression that are only found in cancer tissues are good candidates for being significantly up-regulated in cancer.

To identify genes that are differentially expressed in cancer tissues, we determined gene specificity (*S_i_*) and target specificity (*TS_ij_*) in dataset ***A*** using the complete grouping analysis. Table 1 shows examples of genes represented in cancer tissues at the highest proportion of expression level (greater than 1 in 10,000) and absent from all normal tissues. These genes were only detected in the cancer tissues, with a number of tags (ranging from 54 to 535) in cancer tissues and no tags in normal tissues (maximum *S_i_* in the analysis). To statistically validate the upregulated frequency of these genes, we applied the Fisher exact test [19] with the Bonferroni correction for multiple testing [20] (see Methods). Table 2 presents genes that were detected in only one type of cancer. The identification of these kinds of genes was possible (trough *S_i_* and *TS_ij_*) due to the inclusion of various types of cancer tissues in the analysis. Table S10 shows examples of genes exclusively expressed at relatively high rate in tumor tissues in the analysis of dataset ***C***.

**Table 1:** Genes exclusively detected by the fully grouped analysis in cancer tissues in dataset *A* (human data) at high expression frequency (>0.0001).

Gene Symbol	Gene Description	Frequency *p_ij_*	Tissues	P-value
*SILV*	Silver homolog (mouse)	0.00109391	3	<2.2e–16
*DHRS2*	Dehydrogenase/reductase (SDR family) member 2	0.00024536	4	5.838e–13
*SOX10*	SRY (sex determining region Y)-box 10	0.00023105	2	3.732e–12
*TRAF7*	TNF receptor-associated factor 7	0.00022083	10	9.017e–12
*C10orf2*	Chromosome 10 open reading frame 2 (Twinkle)	0.00020038	8	1.467e–10
*PRPS1*	Phosphoribosyl pyrophosphate synthetase 1	0.00016971	10	5.846e–09
*MLANA*	Melan-A	0.00015949	1	1.311e–08
*AIPL1*	Aryl hydrocarbon receptor interacting protein-like 1	0.00015949	2	1.311e–08
*MAGEA6*	Melanoma antigen family A, 6	0.00015949	5	1.311e–08
*KLHL21*	Kelch-like 21 (Drosophila)	0.00014517	9	8.442e–08
*GNB3*	Guanine nucleotide binding protein (G protein), beta polypeptide 3	0.00014313	2	8.137e–08
*KIFC1*	Kinesin family member C1	0.00014108	10	1.322e–07
*S100B*	S100 calcium binding protein B	0.00013904	2	1.287e–07
*CDT1*	Chromatin licensing and DNA replication factor 1	0.00013086	11	3.23e–07
*ZWINT*	ZW10 interactor antisense	0.00012677	10	8.912e–07
*XAB2*	XPA binding protein 2	0.00011655	9	1.997e–06
*SLC45A2*	Solute carrier family 45, member 2	0.00011041	2	5.299e–06

Frequency *p_ij_* – Relative average frequency of expression in cancer tissues. Tissues **–** Number of cancerous tissues where the gene was expressed in the 12 tissues studied in dataset ***A***. P-value for the Fisher's Exact Test for the frequency of expression of the gene in normal vs. cancer tissues; significant at an α≈0.05 experiment-wise confidence level by Bonferroni correction.

**Table 2:** Examples of genes highly expressed in only one type of cancer in dataset *A.*

Organ	Gen symbol	Gen description	*p_ij_*	P-value
**Eye**	*OTX2*	Orthodenticle homeobox 2	0.00074	0.00131448
**Liver**	*ASGR2*	Asialoglycoprotein receptor 2	0.00063	0.01024596
**Lung**	*T*	T, brachyury homolog (mouse)	0.00050	0.00692187
**Lymph**	*C4orf7*	Chromosome 4 open reading frame 7	0.00065	0.01260259
**Limphr**	*IL9R*	Interleukin 9 receptor	0.00052	0.001396
**Placenta**	*DNMT3L*	DNA (cytosine-5-)-methyltransferase 3-like	0.00084	4.0955e–07
**Skin**	*MLANA*	Melan-A	0.00066	0.00964147

Frequency *p_ij_* – Relative average frequency of expression in cancer tissues. P-value for the Fisher's Exact Test for the frequency of expression of the gene in the normal vs. cancer tissues

## Discussion

The use of information theory tools to quantitatively assess changes in steady state transcript abundances allowed us to examine four different datasets to determine whether cancerous tissues have less transcriptome specialization than their normal counterparts. The results obtained from these analyses showed that specialization of the cancer transcriptome decreased when compared to the normal tissue equivalent. The decrease in transcriptome specialization was due mainly to a reduction in the expression level of genes that are tissue-specific and usually expressed at high levels in normal tissue (see Supporting Text S1 and Table S11, Table S12, Table S13, Table S14, Table S15 and Table S16). These results are in agreement with the observation that tumors often show morphologically dedifferentiated cell types in a manner similar to that observed in stem cells [21]. In addition, molecular evidence has shown that poorly differentiated cancer tumors overexpress genes that are enriched in embryonic stem cells [13]. It is not completely clear whether cancer initiates by a process of de-regulation of organ stem cells or by a de-novo dedifferentiation of organ cells driven by the mutations that arise during the development of the tumor [7].

All high throughput transcriptome studies that used either counting tag strategies or microarrays only measured relative changes in transcription levels. This approach makes the universally accepted assumption that all cells have the same absolute transcriptional activity. However, this assumption lacks experimental validation, especially in the case of cancer cells. The method used in this study measured relative levels of gene expression (the set of *p_ij_*) to assess gene specificity, transcriptome diversity, and specialization. Therefore, we cannot rule out the possibility that all of the genes could have a higher absolute expression level in cancer than in normal tissues. Nevertheless, a general increase in transcription in cancerous cells would not have a major impact in the transcriptome specialization or in the specificity of gene expression.

Transcriptome specialization, δ_i_, is measured exclusively in the context of the organs or tissues included in the analysis and reflects the organ or tissue bias towards the expression of specific genes. To estimate the “true” specialization of a tissue, all distinct cell types of a given organ must be included separately in the analysis. This was not fulfilled in the analysis performed here due to limitations in the data used in this study. A second factor that affects the estimation of specialization is the sample size, or more specifically, the number of gene tags employed. Highly specific genes tend to be expressed in a small subset of the cells that form an organ and thus have a high probability of not having any gene tags and not being present if the sample is relatively small. As a result, specialization tends to be underestimated in small sample sizes. In the case of dataset ***A***, the total number of tags was 620,696, with 131,623 (21%) tags corresponding to normal tissues and the remaining 489,073 (79%) tags corresponding to cancerous tissues. Therefore, the potential for underestimation of specialization was higher for normal tissues than for cancerous tissues. Nevertheless, Figure 1A shows strong evidence of less specialization in the cancerous tissues. This was observed in datasets ***B*** and ***C*** as well.

Human organs are comprised of different numbers and types of cells and therefore have distinct levels of complexity. A more complex organ will have a greater number of distinct cell types, and as a result, the estimation of its diversity and specialization will be less precise and require a larger sample size for accuracy. In contrast, tumors are formed by a small number of distinct cell types and the estimation of its diversity and specialization will be more precise with a given sample size. This is evident by the size of the confidence intervals for each point in Figure 1 (also see Figure S1, Figure S2, Figure S3, Figure S4, Figure S5 and Figure S6). In both cases (datasets ***A*** and ***B***), the size of the confidence intervals is larger for the normal tissues analyzed than for their cancerous counterparts. Nevertheless, the differences in specialization between normal and cancerous tissues are several confidence intervals apart, demonstrating that the conclusions are statistically robust (Table S4, Table S5, Table S6, Table S7 and Table S8).

It is well known that tumor cells are often undifferentiated and resemble embryonic stem cells [9]. To compare the level of specialization of cancer tissues with that of ESCs, we included five libraries of ESCs in dataset ***A*** and analyzed them individually (Figure S1) or as a group (Figure 1A). The position of the ESC in Figure 1 and Figure S1 corroborates that the level of specialization of stem cells is comparable to the majority of the cancer tissues analysed. These data confirm the correlation between the phenotypic dedifferentiation and the drop in specialization in both ESCs and cancer cells. Unfortunately, data showing the degree of dedifferentiation in the distinct tumors analysed in datasets ***A***, ***B,*** and ***C*** were not present in the databases, and therefore we could not infer whether there is a relationship between the degree of dedifferentiation of the tumor and its drop in specialization. However, we hypothesize that this relationship probably exists, since the degree of dedifferentiation of the tumor appears to correlate with the expression of the sets of genes that are enriched in ESCs [13].

We analyzed a library of HSCs as part of dataset ***B***. This library was made from FACS-purified, hematopoietic stem cells obtained from bone marrow and represents cells that can differentiate into myelomonocytic cells, B cells, or T cells. In contrast with the ESCs of Figure 1, these cells originated from a specialized adult organ. As shown in Figure 1B, HSCs have a level of estimated specialization comparable to that of normal lung and higher than that of normal skin. This indicates that relatively undifferentiated cell types can present a relatively high specialization of the transcriptome. Our conclusion is also supported by the transcriptome analysis of normal lymphatic tissues (Lymph and Lymphr; Figure 1A).

We propose that the fitness of a pre-cancerous cell, in the context of a tumor, will be increased if the genes related to the original function of the parental tissue are switched off, because this highly expressed and specific set of genes represents a high cost in energy and resources that would be disadvantageous in the context of the tumor. Our hypothesis suggests that if the expression of these highly expressed and specific genes is reduced or turned off, then a decrease in tissue specialization should be observed. Dissecting the reduction in specialization through analysis of the individual genetic components will provide a better understanding of carcinogenesis. Moreover, if the drop in expression of at least some of these genes precedes morphological changes in the pre-cancerous cells, the drop could be exploited for diagnostic proposes. Our hypothesis is not contradicted by the observation of dedifferentiation in cancer tissues, but rather parallels this finding: tissues with a greater dedifferentiated phenotype will express a less specialized transcriptome.

The analysis in dataset ***C*** was performed on loci that were grouped by chromosomes from a heterogeneous mixture of tissues classified only as “normal” or “tumor”. Therefore, the specificity of the loci is only estimated with regard to this criterion and not with regard to the organ of origin as in datasets ***A*** and ***B.*** As a consequence, the specialization estimated for the “normal” and “tumor” tissues is much smaller than the rank of specialization estimated when the organ of origin is taken into account (compare figures 1 and 2). Despite smaller differences in specialization between normal and cancerous tissues in dataset ***C***, the data are statistically significant for all chromosomes (except for chromosome Y) and all cases, except for chromosome 18, indicate that a drop in specialization occurs in tumors (Table S8). Interestingly, chromosome 18 contains several tumor suppressor genes including *DDC*, *DPC4,* and *JV18-1/MADR2* [22], and therefore the high expression of these genes could drive the observed increase in specialization (see Table S15). Taken together, these data serve an independent confirmation of the hypothesis that transcriptome specialization diminishes in tumors. We predict that enhanced understanding of the mechanisms responsible for the drop in specialization that occurs in tumors through better characterization of cancer transcriptome profiles will lead to the development of new molecular diagnosis tools and intervention techniques.

From the analysis of grouped normal and cancerous tissues in dataset ***A*** (“complete grouping”; see Methods) we detected 14,573 genes (52%) out of a total of 28,087 genes that were represented in either normal or cancerous tissues only (estimated gene specificity *S_i_* = 1). Of these genes with maximum specificity, 6,220 (43%) were detected exclusively in cancer and the remaining 8,353 (57%) were detected exclusively in normal tissues. Our observations that particular genes were found in only one specific group (normal or cancerous tissues) were dependent upon the sample size and therefore required statistical analyses to determine the significance. The Fisher's exact test with Bonferroni correction (see Methods) concluded that only 17 of the genes that were exclusively detected in cancerous tissues were significantly upregulated. These genes are presented in Table 1. Table S17 presents the Gene Ontology classifications for the genes presented in Table 1.

If information theory indexes are effective in identifying genes upregulated in cancer, they should also detect genes that have previously been reported to be associated with cancer. This was indeed the case, as the list of genes exclusively detected in cancer (Table 1), including *TRAF7*, *PRPS1*, *CDT1,* and *ZWINT,* were previously reported as cancer marker genes [23], [24], [25], [26]. More importantly, this quantitative approach identifies genes potentially involved in cancer that have not been previously identified, such as *KLHL21*, *KIFC1*, and *XAB2* (Table 1). A description of the genes listed in Table 1 is presented in Supporting Text S1.

The genes listed in Table 2 were found to be present in only one type of cancer at significantly high levels of expression (grouped analysis, dataset ***A***) and exemplify the rich possibilities of data-mining using specificity (*S_i_*) and target specificity (*TS_ij_*) of gene expression. Among these genes, we found examples of cancer markers (*MLANA)* (also reported in Table 1), a recently described oncogene (*OTX2*) [27], and a gene used as a predictor of circulating tumor cells (*ASGR2*) [28]. In lung cancer, we identified a gene (*T*, the human T brachyury homologue) that has been reported to be epigenetically silenced in non-small-cell lung cancer [29], and in lymph cancer, we found a gene (*C4orf7)* that was previously reported to have significantly high expression in lymph node metastases [30]. Table S18 presents the Gene Ontology classifications for the genes presented in Table 2.

The analysis performed here did not take into account the mRNA splicing that forms distinct proteins, since the tags employed were only annotated at the gene level in the datasets. Further investigation is needed to assess the effect of splicing deregulation in cancer over the transcriptome diversity and specialization.

In the future, a more detailed functional analysis of genes with altered expression in cancer will provide a better understanding of their role in this dynamic process.

### General conclusions

Our present data advance the current hypothesis that cancer diminishes the specialization of affected tissues. This suggests that although cancer tissues gain specialized functions in cell cycle control, angiogenesis, and metastasis, they concomitantly exhibit a loss of specialization and deregulation of sets of genes with tissue-specific functions.

The application of gene specificity and target specificity provides a powerful, practical tool for data mining of the numerous studies that have already compared gene expression in normal and cancerous tissues. We have shown how this method has the capability to recover not only genes well known to be associated with cancer, but also less understood genes that are highly expressed and up-regulated in neoplasia. This approach will help us understand the process of tumor development and hopefully provide new possibilities for intervention with drugs and genomic medicine. These same tools can be used to identify genes that are down-regulated in cancer or control genes that remain constant during the process. In addition, these information tools can be easily adapted to existing web pages that show transcription results from counting gene tag strategies as well as microarrays experiments.

## Materials and Methods

### Datasets

#### Dataset *A* – Human expression data from the “Cancer Genome Anatomy Project”

The data are from the “Cancer Genome Anatomy Project” (http://cgap.nci.nih.gov/) [15] and were downloaded from the site ftp://ftp1.nci.nih.gov/pub/CGAP/ in July 2008. The data consist of diverse libraries of cDNA with expression profiles (specific genes identified and the number of tags found) for distinct human organs under diverse conditions. The data were downloaded and placed into a MySQL relational database for selection. The following criteria were applied in order to select the human data to be analyzed: 1) Only non-normalized libraries that reflected the true gene expression were considered; 2) the libraries had to contain at least 5000 gene tags; 3) only libraries derived from a single organ or tissue were considered (libraries from mixed organs and cell lines were not allowed); and 4) each library had to have a comparable library from normal tissue to be considered. In addition to the cancer libraries and their normal counterparts, we included five libraries of ESCs that were considered suitable for comparison of the specialization of non-differentiated cell types. These libraries also fulfilled the criteria (1–3) described above. Dataset ***A*** included 53 libraries from 13 distinct tissues that had a total of 671,197 gene tags for 28,087 human genes that fulfilled the above criteria. Table S1 presents the main characteristics of the libraries.

Table S1 indicates that in two cases (placenta and testis), two libraries from normal tissues of the same organ were selected. In various cases more than one library from a neoplasia were selected from the same tissue. Table S1 also shows that there are highly variable numbers of gene tags in each library, ranging from 5,003 in the testis library to 37,803 in the Skin library. On average, the libraries have 12,931 gene tags. The total number of distinct human genes represented in at least one of the libraries was 28,087.

#### Dataset *B* – Mouse expression data from the “Cancer Genome Anatomy Project”

Dataset ***B*** represents mouse data downloaded from the “Cancer Genome Anatomy Project” from the same site and the same date. The selection of libraries from this set followed the same criteria set for dataset ***A***. Dataset ***B*** also included a library of hematopoietic stem cells (HSC). Dataset ***B*** was comprised of 30 libraries from 6 distinct tissues with a total of 541,453 expressed gene tags for 25,044 distinct mouse genes that fulfilled the selection conditions described above. Table S2 presents the main characteristics of the libraries.

#### Dataset *C* – Human normal and tumor tissues from the “Human Transcriptome Map” (HTM)

The HTM project [Caron, 2001 #111] (http://bioinfo.amc.uva.nl/HTMseq/controller) integrates mapping data with genome-wide messenger RNA expression profiles as provided by serial analysis of gene expression (SAGE). The data consist of map and expression information and were downloaded by chromosome from the site in July 2008. For this study, we downloaded the dataset titled “All tissues normal” that contained 5,747,834 tags from normal human tissues and the dataset titled “All tissues tumor” that contained 12,861,239 tags from diverse cancer tumors. The combined datasets represented a total of 62,916 loci distributed along the human genome. The data were separately downloaded for each of the 24 chromosomes (Chromosomes 1 to 22, X, and Y) in files normalized to rates of 10,000 tags. The files were included in a relational MySQL database and re-converted to the original number of expressed tags in each library. The data were then placed in one file for each chromosome and a master file containing the data for all chromosomes. Table S3 presents the number of tags per chromosome in each of the two libraries (normal and tumor).

#### Dataset *D* – Human microarray data of normal and precancerous states in breast tissue

The dataset ***D*** consisted of a set of 16 microarrays from paired samples of normal, terminal duct lobular units (TDLUs; 8 samples) and hyperplastic, enlarged lobular units (HELUs; 8 samples) from RNA samples obtained by microdissection [Lee, 2007 #108]. The data were downloaded from the GEO database at the NCBI (GEO accession GDS2739; http://www.ncbi.nlm.nih.gov/) in July 2008. This dataset has expression information for 34,702 human genes in each of the 16 arrays.

### Information theory and statistical analyses

If we consider the relative frequencies of transcription *p_ij_* for the *i-th* gene (*i* = *1, 2, …, g*) in the *j-th* tissue or transcriptome (*j* = *1, 2, …, t*), then the diversity of the transcriptome of each tissue can be quantified by an adaptation of Shannon's entropy formula,
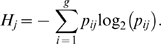



*H_j_* will vary from zero when only one gene is transcribed up to *log_2_(g)* where all *g* genes are transcribed at the same frequency: *1/g*. If we consider the average frequency of the *i-th* gene among tissues, say,
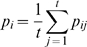



we can define gene specificity as
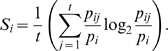



*S_i_* will attain a value of zero if the gene is transcribed at the same frequency in all tissues and a maximum value of *log_2_(t)* if the gene is exclusively expressed in a single tissue. Tissue specialization is then measured, for the *j-th* tissue, as the average gene specificity, say
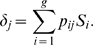



These formulas were previously described [14]. For further details see Mathematical Addendum in Supporting Text S1.

To apply the analysis of information parameters of the transcriptome, estimating its diversity, *H_j_*, and specialization, *δ_j_*, as well as the gene specificities, *S_i_*, and target specificities, *TS_ij_*, it is necessary to have estimates, *p_ij_*, of the relative frequency of expression of the *i-th* gene in the *j-th* transcriptome, where *i* = *1, 2, …, g*, the total number of genes, and *j* = *1, 2, …, t*, the total number of transcriptomes (tissues) studied [14] (see also Mathematical Addendum in Supporting Text S1). For datasets ***A***, ***B,*** and ***C***, which are the product of counting strategies, the values of *p_ij_* were obtained by dividing the number of tags for gene *i* in the *j* transcriptome (library or set of libraries) by the total number of tags obtained in the transcriptome *j*. For microarray data (dataset ***D***; see Supporting Text S1), the *p_ij_* were obtained by dividing the normalized signal obtained for gene *i* in the *j* transcriptome by the sum of normalized signals in the transcriptome *j* (microarray slide *j*). Using the matrices, {*p_ij_*}, the analysis was performed by using the statistical environment R [31], by use of a set of functions developed to such effect.

To obtain confidence intervals for the information parameters in the datasets ***A***, ***B***, and ***C***, we applied the bootstrap procedure [32]. This assumed a multinomial distribution for the number of tags in the transcriptome, and obtained B = 2000 bootstrap replicates, which are samples from the multinomial distribution where the total number of tags in the transcriptome, *n_j_*, and the observed relative frequencies, {*p_ij_*}, are assumed as parametric values. The assumption of multinomial distribution for the number of tags representing each gene in a transcriptome appears reasonable even if it most likely underestimates the real biological variation and possible dependencies in sets of genes present in the transcriptome. Having obtained the set of B = 2000 bootstrap replicates of each parameter, we applied the Bootstrap Percentile Method [32] at 95% confidence level to obtain the confidence intervals plotted in the figures presented (Figure 1A, Figure 1B, and supporting Figure S1, Figure S2, Figure S3, Figure S4, Figure S5, Figure S6, Figure S7, Figure S8, Figure S9 and Figure S10).

Using the same bootstrap approach, we obtained approximated 99% confidence intervals for the true differences between the specialization of pairs of transcriptomes, for example *Δ_mn_* = *δ_m_*−*δ_n_*, where *m* and *n* are the transcriptomes of interest (normal and cancer tissues), for which the hypothesis of equal specialization (H0: *Δ_mn_* = 0) needed to be tested. The null hypothesis of identical specialization was rejected when the approximated 99% confidence interval did not include the value of 0. We also tested the assumption of normality for the bootstrap estimates by means of the Shapiro-Wilks test [33] (Table S4, Table S5, Table S6, Table S7 and Table S8).

The estimation of confidence intervals and hypothesis testing was not feasible for the microarray data (dataset ***D***), since in that case, the original data belonged to an unknown continuous distribution, and the assumption of a given distribution is difficult without having a very good estimate of the (unknown) variances and covariances for the gene expression levels. The absence of true replicates in these data (dataset ***D***) made it impossible to obtain confidence intervals for the parameters or to perform statistical tests on the specialization differences.

### Dataset Grouping

The analysis for datasets ***A*** and ***B*** were done at three levels of grouping. First, the “ungrouped” analysis was performed by considering each of the individual libraries (Table S1 for dataset ***A*** and Table S2 for dataset ***B***), with comparisons between the levels of specialization being done between the normal and cancerous states in libraries of the same tissue (Figure S1, Figure S2, Figure S4, and Figure S5). The second level, designated here as “grouped analysis”, corresponds to grouping libraries that were from the same tissue and state (normal or cancerous). This was done by adding the tags for each gene in the corresponding datasets. This is a valid procedure since the libraries are independently obtained from the same organ in approximately the same tissue state (*normal* or *cancerous*). In addition, the variations that are disregarded correspond to biological differences between individuals from which the samples were obtained, putative differences in methodology of sequencing, and random variation. The third level of grouping, “complete grouping”, added tags from all distinct tissues in the same state (*normal* or *cancerous*) to form only two groups: normal and cancerous states. This “complete grouping” disregards or confounds the transcriptome variation given by the different nature of the tissues, but allows for the analysis of differences between cancerous and normal tissues, which is the main source of this study (Figure S3 and Figure S6). This procedure is analogous to the collapse of contingency tables routinely performed in the statistical analysis of discrete data [19].

To evaluate the statistical significance of genes exclusively detected in cancer in dataset ***A*** (Table 1) we used the Fisher's exact test [19] on the 2×2 contingency table produced by grouping the tags belonging to the categories “normal” or “cancer” and “gene I” or “gene no-I” for each one of the 6,220 genes found to be exclusively detected in cancer. Given that we were performing 6,220 tests, the Bonferroni correction for multiple testing [20] was applied by dividing the desired experimental-wise error Type I (α = 0.05) between the number of tests to be performed, for example 0.05/6,220≈7.5e–6. Only genes with P-value less than 7.5e–6 in the Fisher's exact test are presented in Table 1. The same procedure was performed to obtain the significance (P-value) for the data of Table 2, except that in this case, no correction was needed since only one gene was tested for each (independent) set of tags in a given type of cancer.

For dataset ***C***, which corresponds to human data for normal and tumor tissues grouped by chromosome, an individual analysis was performed for the loci in each chromosome followed by a complete analysis for all loci together. This analysis was performed on the non-normalized tags, since the information analysis normalizes the data by taking the relative frequencies of expression of each gene (*p_ij_*). As in the cases of datasets ***A*** and ***B***, a bootstrap analysis was performed to obtain confidence intervals for the parameters of interest and to test the differences in specialization between normal and tumor tissues.

## Supporting Information

Text S1 Includes supporting text, supporting methods, supporting discussion, mathematical addendum, functions source code and supporting references.
(0.88 MB PDF)

Figure S1 Estimated values of *Hj* (diversity) and *δj* (specialization) in each one of the libraries of dataset ***A***, ungrouped analysis.
(0.22 MB PDF)

Figure S2 Estimated values of *Hj* (diversity) and *δj* (specialization) in each one of the libraries of dataset ***A***, non-grouped analysis. One panel per organ.
(0.03 MB PDF)

Figure S3 Estimated values of *Hj* (diversity) and *δj* (specialization) in normal and cancerous transcriptomes obtained by grouping all organs in dataset ***A***.
(0.05 MB PDF)

Figure S4 Estimated values of *Hj* (diversity) and *δj* (specialization) in each one of the libraries of dataset ***B*** (mouse data), non-grouped analysis.
(0.07 MB PDF)

Figure S5 Estimated values of *Hj* (diversity) and *δj* (specialization) in each one of the libraries of dataset ***B*** (mouse data), non-grouped analysis. One panel per organ.
(0.08 MB PDF)

Figure S6 Estimated values of *Hj* (diversity) and *δj* (specialization) in normal and cancerous transcriptomes obtained by grouping all organs in dataset ***B*** (mouse data).
(0.05 MB PDF)

Figure S7 Estimated values of *Hj* (diversity) and *δj* (specialization) in the chromosome Y (dataset ***C***).
(0.05 MB PDF)

Figure S8 Estimated values of *Hj* (diversity) and *δj* (specialization) in chromosomes 21, 18, 13, 22, 20 and X (dataset ***C***).
(0.05 MB PDF)

Figure S9 Estimated values of *Hj* (diversity) and *δj* (specialization) in chromosomes 14, 8, 15, 9, 10, 16, 4, 6, 7, 5, 11, 3, 12, 17, 19 and 2. Dataset ***C***.
(0.06 MB PDF)

Figure S10 Estimated values of *Hj* (diversity) and *δj* (specialization) in chromosomes 1 and the set of all chromosomes taken together. Dataset ***C***.
(0.05 MB PDF)

Figure S11 Example of scatter plot for the differences in expression in Dataset ***A*** (Human dataset).
(0.36 MB PDF)

Figure S12 Example of scatter plot for the differences in expression in Dataset ***B*** (Mouse dataset).
(0.38 MB PDF)

Figure S13 Scatter plot of *Hj* (Diversity) and *δj* (Specialization) in transcriptomes of normal (blue) and precancerous (red) tissues in dataset ***D***.
(0.10 MB PDF)

Table S1 Human libraries from the "Cancer Genome Anatomy Project" selected for analysis (Dataset ***A***).
(0.07 MB PDF)

Table S2 Mouse libraries from the "Cancer Genome Anatomy Project" selected for analysis (Dataset ***B)***.
(0.05 MB PDF)

Table S3 Number of tags and loci per chromosome in dataset ***C*** (Human normal and tumor tissues from the "Human Transcriptome Map").
(0.02 MB PDF)

Table S4 Approximate 99% Confidence Intervals for the difference between specializations in all pairs of comparable tissues (normal versus cancer) in dataset ***A*** (grouped analysis).
(0.02 MB PDF)

Table S5 Approximate 99% Confidence Intervals for the difference between specializations in all pairs of comparable libraries (non-grouped analysis, dataset ***A***).
(0.03 MB PDF)

Table S6 Approximate 99% Confidence Intervals for the difference between specializations in all pairs of comparable tissues (normal versus cancer) in the ***B*** dataset (mouse data); grouped analysis.
(0.01 MB PDF)

Table S7 Approximate 99% Confidence Intervals for the difference between specializations in all pairs of comparable libraries in dataset ***B*** (mouse data); non-grouped analysis.
(0.03 MB PDF)

Table S8 Approximate 99% Confidence interval for the differences in specialization between normal and tumor tissues in chromosomes in the analysis of dataset ***C*** (Human Transcriptome Map).
(0.02 MB PDF)

Table S9 Number of tags and loci per chromosome in dataset ***C*** (Human normal and tumor tissues from the "Human Transcriptome Map").
(0.02 MB PDF)

Table S10 Examples of genes exclusively expressed at relatively high rate in tumor tissues in the analysis of dataset ***C*** (HTM).
(0.02 MB PDF)

Table S11 Statistical analyses of genes over expressed in normal and cancer tissues in the human dataset ***A*** with regard to their specificity and differences in frequency of expression.
(0.08 MB PDF)

Table S12 The ten genes with largest influence (See Eq. 8) in the change of specialization of cancerous tissues per organ. Dataset ***A*** (human dataset).
(0.14 MB PDF)

Table S13 Statistical analyses of genes over expressed in normal and cancer tissues in the mouse dataset (***B***) with regard to their specificity and differences in frequency of expression.
(0.07 MB PDF)

Table S14 The ten genes with largest influence (See Eq. 8) in the change of specialization of cancerous tissues per organ. Dataset ***B*** (mouse dataset).
(0.10 MB PDF)

Table S15 Statistical analyses of loci over expressed in normal and tumor tissues in Chromosome 18 of dataset (***C***) with regard to their specificity and differences in frequency of expression.
(0.06 MB PDF)

Table S16 The ten most influential loci in the increase of specialization of tumors in Chromosome 18 (dataset ***C***).
(0.05 MB PDF)

Table S17 Classifications by Gene Ontology of the genes presented in Table 1.
(0.05 MB PDF)

Table S18 Classifications by Gene Ontology for the genes presented in Table 2.
(0.05 MB PDF)

## References

[1] Klausner RD (2002). The fabric of cancer cell biology–Weaving together the strands.. Cancer Cell.

[2] Mihich E, Feunteun J, Friend S (2002). Thirteenth Annual Pezcoller Symposium: Focusing Analytical Tools on Complexity in Cancer.. Cancer Res.

[3] Porter D, Polyak K (2003). Cancer target discovery using SAGE.. Expert Opinion on Therapeutic Targets.

[4] Brentani H, Caballero OL, Camargo AA, da Silva AM, da Silva WA (2003). The generation and utilization of a cancer-oriented representation of the human transcriptome by using expressed sequence tags.. PNAS.

[5] Rhodes DR, Yu J, Shanker K, Deshpande N, Varambally R (2004). Large-scale meta-analysis of cancer microarray data identifies common transcriptional profiles of neoplastic transformation and progression.. Proceedings of the National Academy of Sciences of the United States of America.

[6] Majeti R, Becker MW, Tian Q, Lee T-LM, Yan X (2009). Dysregulated gene expression networks in human acute myelogenous leukemia stem cells..

[7] Maley CC, Galipeau PC, Finley JC, Wongsurawat VJ, Li X (2006). Genetic clonal diversity predicts progression to esophageal adenocarcinoma.. Nature Genetics.

[8] Gatenby RA, Gillies RJ (2008). A microenvironmental model of carcinogenesis.. Nat Rev Cancer.

[9] Sell S (2004). Stem cell origin of cancer and differentiation therapy.. Crit Rev Oncol Hematol.

[10] Sell S (1993). Cellular origin of cancer: dedifferentiation or stem cell maturation arrest?. Environ Health Perspect.

[11] Reya T, Morrison SJ, Clarke MF, Weissman IL (2001). Stem cells, cancer, and cancer stem cells.. Nature.

[12] Kelly PN, Dakic A, Adams JM, Nutt SL, Strasser A (2007). Tumor growth need not be driven by rare cancer stem cells.. Science.

[13] Ben-Porath I, Thomson MW, Carey VJ, Ge R, Bell GW (2008). An embryonic stem cell-like gene expression signature in poorly differentiated aggressive human tumors.. Nat Genet.

[14] Martínez O, Reyes-Valdés H (2008). Defining diversity, specialization, and gene specificity in transcriptomes through information theory.. Proceedings of the National Academy of Sciences.

[15] Riggins GJ, Strausberg RL (2001). Genome and genetic resources from the Cancer Genome Anatomy Project.. Hum Mol Genet.

[16] Caron H, Schaik Bv, Mee Mvd, Baas F, Riggins G (2001). The Human Transcriptome Map: Clustering of Highly Expressed Genes in Chromosomal Domains.. Science.

[17] Lee S, Medina D, Tsimelzon A, Mohsin SK, Mao S (2007). Alterations of Gene Expression in the Development of Early Hyperplastic Precursors of Breast Cancer.. Am J Pathol.

[18] t Hoen PAC, Ariyurek Y, Thygesen HH, Vreugdenhil E, Vossen RHAM (2008). Deep sequencing-based expression analysis shows major advances in robustness, resolution and inter-lab portability over five microarray platforms.. Nucl Acids Res.

[19] Everitt BS (1992).

[20] Ewens WJ, Grant GR (2001).

[21] Beachy PA, Karhadkar SS, Berman DM (2004). Tissue repair and stem cell renewal in carcinogenesis.. Nature.

[22] Wodarz D, Komarova NL (2005).

[23] Obuse C, Iwasaki O, Kiyomitsu T, Goshima G, Toyoda Y (2004). A conserved Mis12 centromere complex is linked to heterochromatic HP1 and outer kinetochore protein Zwint-1.. Nature Cell Biology.

[24] Reyes I, Tiwari R, Geliebter J, Reyes N (2007). DNA microarray analysis reveals metastasis-associated genes in rat prostate cancer cell lines.. Biomédica.

[25] Xouri G, Lygerou Z, Nishitani H, Pachnis V, Nurse P (2004). Cdt1 and geminin are down-regulated upon cell cycle exit and are over-expressed in cancer-derived cell lines.. European Journal of Biochemistry.

[26] Xu L-G, Li L-Y, Shu H-B (2004). TRAF7 Potentiates MEKK3-induced AP1 and CHOP Activation and Induces Apoptosis.. J Biol Chem.

[27] Di C, Liao S, Adamson DC, Parrett TJ, Broderick DK (2005). Identification of OTX2 as a Medulloblastoma Oncogene Whose Product can be Targeted by All-Trans Retinoic Acid.. Cancer Res.

[28] Smirnov DA, Zweitzig DR, Foulk BW, Miller MC, Doyle GV (2005). Global Gene Expression Profiling of Circulating Tumor Cells.. Cancer Res.

[29] Park JC, Chae YK, Son CH, Kim MS, Lee J (2008). Epigenetic silencing of human T (brachyury homologue) gene in non-small-cell lung cancer.. Biochemical and Biophysical Research Communications.

[30] Inamura K, Shimoji T, Ninomiya H, Hiramatsu M, Okui M (2007). A metastatic signature in entire lung adenocarcinomas irrespective of morphological heterogeneity.. Human Pathology.

[31] R-Development-Core-Team (2005). R: A language and environment for statistical computing..

[32] Efron B, Tibshirani RJ (1993). An introduction to the bootstrap; Cox DR, Hinkley DV, editors..

[33] Royston P (1982). An Extension of Shapiro and Wilk's W Test for Normality to Large Samples.. Applied Statistics.

