# Supplementary material for: Cancer Reduces Transcriptome Specialization
Source: PLoS One. 2010 May 3;5(5):e10398. doi: 10.1371/journal.pone.0010398 (PMC2862708; doi:10.1371/journal.pone.0010398)
Supplement: Text S1 — Includes supporting text, supporting methods, supporting discussion, mathematical addendum, functions source code and supporting references. (0.88 MB PDF) [file pone.0010398.s001.pdf]

Supporting Text

General considerations about cancer specialization

Hanahan and Weinberg suggested that the large diversity of cancer cell genotypes is a manifestation of six essential alterations in cell physiology that collectively dictate malignant growth: self-sufficiency in growth signals, insensitivity to growth-inhibitory (antigrowth) signals, evasion of programmed cell death (apoptosis), limitless replicative potential, sustained angiogenesis, and tissue invasion and metastasis [1]. These alterations imply the de-regulation of large sets of genes that are otherwise strictly coordinated to produce the physiological activities of normal tissues and organs. Cells in cancer tissues lose many of the normal physiological and morphological qualities that determine its tissue identity; they also gain new capacities as the ones mentioned above. The question is to which side the specialization balance will the cells go? Will they gain specialization by the characteristics acquired during the carcinogenesis process? Or, on the contrary, they will show a diminution of specialization caused by the de-regulation of the sets of genes that determined their normal behavior. The information theory tools permit to test and answer this disjunctive.

There are diverse sources of variation that can affect the diversity and specialization of the transcriptomes. First, the organ of origin is a main source of transcriptome variation, because distinct genes are expressed at different rates depending on the function performed by the involved cells; we have shown that this is the case in normal human tissues [2]. Probably next in importance are the development and physiological state of the organ, followed by genetic differences among the individuals sampled and the unavoidable random variation in the transcription rates. We need to add to these sources of variation the statistical error caused by sampling a rather small number of transcript tags, compared with the number of transcripts existent in the cells, or by indirectly measuring the transcription levels by means of microarrays. Here we are concerned with the hypothesis that the cancer tissues have a smaller level of specialization than their normal counterparts. This hypothesis seems reasonable because, in general terms, the cells in neoplasias do not fulfill the same (specialized) functions that are performed in normal tissues and this must imply the de-regulation in a large number of genes that, in turn, must decrease the tissue specialization measured by the average gene specificity, δ_j. As mentioned above a counter acting force for this diminution in specialization will be the activation of pathways related to mitosis and other functions that are gained by the cancer tissues in comparisons with their normal counterparts.

It has been demonstrated that cancer cell lines in general lose the tissue-specific up-regulation of genes, and distinct lines for the same cancer tissue are often very variable, to the extent that they do not reflect the transcriptome of the original tumor [3], thus cancer cell lines were not included in the analysis limiting the comparison to the transcriptomes of normal tissues and cancer tumors, but including embryonic stem cells (dataset ***A***, see Table S1) and hematopoietic stem cells of bone marrow origin (dataset ***B***, see Table S2) .

Dataset ***A*** – Human expression data from the "Cancer Genome Anatomy Project".
Figure 1 ***A*** in the main text presents the diversity *versus* specialization scatter plot resulting from the "grouped analysis" of dataset ***A***, including the approximate 95% confidence limits for the parameters, and Table S4 present the approximate 99% confidence intervals for the differences in specialization between comparable tissues. From Table S4 we can see that all differences in specialization between normal and cancerous tissues are statistically significant at $P<0.01$, because none of the 99% confidence intervals include the value of zero.

From Figure 1 ***A*** we notice that ten of the twelve normal tissues have larger specialization than the cancerous tissues, the exceptions being the Eye and Lymphr (See Table S1 for a description of the libraries). When comparing by pairs of analogous tissues in Figure 1 A –points linked with discontinuous lines, the only cancerous tissue that presents an estimated specialization larger that its normal counterpart is the Eye. This case is possibly due to the fact that many more tags (42,029) were obtained from the cancerous states than from the normal tissue (10,679) (Table S1), and thus the specialization of the normal eye transcriptome is severely underestimated, probably by the absence of many low expressed transcripts specific to the eye. This is partially confirmed by the fact that in the analysis of the individual libraries (Figure S1 and S2) the library from the cancerous eye with larger number of tags (Lib. No. 5606 with 26,788 tags, Table S1) has a higher specialization than the normal eye (Lib. No. 7269 with 10,679 tags) while the other library from a cancerous tissue (Lib. No. 5374 with 15,241 tags, Table S1) has a lower specialization than the normal tissue.

Ungrouped and complete grouping analysis of dataset *A* (Human tissues from the "Cancer Genome Anatomy Project")

From Figure S1 and FigureS2, corresponding to the "ungrouped analysis" of dataset ***A***, we can see that in general the 95% confidence intervals for the values of H_j (diversity) and δ_j (specialization) are small enough to infer a clear separation of the transcriptomes in the diversity x specialization space. However

there are cases where the intervals for H_j or δ_j are partially overlapped, but not cases where both intervals are overlapped at the same time which will mean that the transcriptomes were undistinguishable in diversity and specialization.

In the “ungrouped analysis” there are 39 comparisons possible between a healthy tissue and a corresponding neoplasia. From these 39 comparisons (Table S5), 37 (95%) show a larger specialization, δ_j, in the normal tissue when compared with the cancer state. To establish if the observed differences were statically significant we obtained approximated 95% confidence intervals for the differences in specialization. In Table S5 we can observe the confidence intervals for these differences and in 38 of the 39 cases the intervals does not contain the value of zero, implying the statistical significance of these differences. The exception is the difference in specialization between the libraries Lymphr and LymphrC1 that does not result significant. The largest differences in specialization (1.83 and 1.86) are exhibited between the liver and its two cancerous states respectively. In coincidence with this analysis, the liver was estimated to be the most specialized organ among 36 organs studied by DNA microarrays (see Figure S4 in [2]). In contrast, the two smallest and negative differences in specialization are -0.19 and -0.18, between Eye-EyeC2 and Skin-SkinC7. Even when these two results contradict the hypothesis that the cancerous state diminish the transcriptome specialization it is worth noting that for both tissues there are cases where the hypothesis is supported by other comparisons, one for eye (Eye-EyeC1 in Table S5) and six for skin (Skin versus the cancerous states C1 to C6; Table S5). If the hypothesis of decreasing specialization in cancerous states were false, we will expect a 19/38 (50%) chance to obtain significant results in that direction (discounting the non-significant differences; one in this case). In contrast we obtained 36/38 (95%) of comparisons in favor of the hypothesis of decreasing specialization in cancer states. Only in one case in Table S5 (Liver - LiverC2) the Shapiro-Wilks test for normality shows marginal significance, implying that in all other cases the distribution of the bootstrap estimates for the differences in specialization are distributed normally.

In the “complete grouping” analysis we lose all the information about the differences of expression per organ, but we will conserve the differences relevant for the normal versus cancerous states. Given that the original tags are added, the relative weight of each library in the final results is given by the total number of tags in the library (see Table S1), thus libraries with larger number of tags have more influence than those with a smaller number of tags. However, the results are basically the same if the same weight is given to each library, independently of its number of tags (data not shown). Figure S3 shows the result of the “complete grouping analysis” in this dataset.

From Figure S3 we can see a marked drop in both, H_j (diversity) and δ_j (specialization) in the transcriptomes of cancerous tissues when compared with normal ones. The tests for these differences were significant at the 99% confidence level (data not shown), and permit to infer that in general we can expect a loss of transcriptome specialization when the tissues develop from a normal to a cancerous state.

Use of S_i and TS_{ij} as data mining tools

Gene specificity, S_i, indicates, in an appropriate scale with a definite maximum ($log_2(t)$), how particular is the expression of the *i-th* gene among the transcriptomes analyzed. However, it does not point exactly to which transcriptome is this gene specific. On the other hand, the target specificity coefficient, TS_{ij}, lineally decompose the value of S_i in the components corresponding to each *j-th* transcriptome, measuring the specificity of the *i-th* gene in the *j-th* transcriptome, allowing to know how the specificity of the gene is shared among the transcriptomes and pinpointing exactly to which transcriptome is the gene specific, in the cases where the specificity reaches its maximum value. In is important to underline that the fact that an estimated value of S_i reaches it maximum in a given dataset does not imply that the gene is exclusively expressed in a given transcriptome, it only signals to the fact that in the sample studied tags for that gene were only found in one of the transcriptomes. Then it is necessary to test the null hypothesis H_0: $S_i > 0$, that is, the hypothesis of null specificity for the gene. This hypothesis can be tested via the independence hypothesis in a 2 x 2 contingency table, formed by grouping the gene tags in the categories "transcriptome *j*" and "not transcriptome *j*" (columns) and by rows "gene *i*" and "not gene *i*", where transcriptome *j* is the one where zero tags of the gene were expressed. This hypothesis can be tested for all values of S_i, even if S_i did not reached its maximum, but if many genes age tested then a correction for multi-testing, as the Bonferroni correction, must be employed (see Methods).

Discussion of some genes upregulated in cancer

Here we discuss a set of genes found to be upregulated in cancer in dataset ***A*** (human data) at relatively high expression frequency (Table 1).

The *KIFC1* (Kinesin family member C1) gene was present in 10 of the 12 cancer tissues, and undetected only in prostate and one of the lymph cancers. Kinesin superfamily proteins (KIFs) are motor proteins that transport membranous organelles and macromolecules fundamental for cellular functions along microtubules [4]. Specifically *KIFC1* is a putative mitotic motor [5] that has been proven to interacts with *KIF5B* and both are required for motility and fission of early endocytic vesicles

in mouse liver [6]. Interestingly, in a microarray experiment *KIFC1* is reported within a cluster of genes under-expressed in primary tumor tissue of breast cancer and tumor cultures when compared to immortal cell lines of the same kind of cancer [7], implying that this gene is highly up-regulated in the immortal cell lines of breast cancer studied. Even when there is a report of the use of an antisense oligonucleotide library to identify and validate a kinesin-like gene (Eg5) as a target for antineoplastic drug development [8], the understanding of the role of *KIFC1* in cancer appears to be poorly represented in the cancer literature. KIFC1 is classified by Gene Ontology with regard to biological process (BP), as a mitotic sister chromatid segregation, with molecular function (MF) associated to nucleotide binding and cellular component (CC) to nucleus (Table S17).

The *C10orf2* (Chromosome 10 open reading frame 2), also known as *PEO1* [9], encodes a putative helicase (Twinkle), which is related to the product of bacteriophage T7 gene 4, and co-localizes with mitochondrial DNA. Mutations in *C10orf2* may be associated with accelerated mitochondrial aging by increment of mutations and deletions of the mtDNA, therefore the function of Twinkle is inferred to be critical for lifetime maintenance of human mtDNA integrity [10]. The discovery of Twinkle and its mutant was labeled as a significant step towards understanding the complex group of disorders involving nuclear-mitochondrial miscommunication [11]. The *C10orf2* was present in eight of the twelve cancers studied (bone, eye, liver, lung, lymph, placenta, prostate and skin) and undetected in all normal tissues, thus it appears that *C10orf2* could be a promising biological marker of some types of malignancies. *C10orf2* do not have suitable Gen Ontology classifications

The *KLHL21* (Kelch-like 21) gene was found in 9 of the 12 cancer tissues studied, being non-detected only in bone, and the two lymph cancers studied. The Drosophila Kelch protein is required to maintain actin organization in ovarian ring canals [12]. In humans the kelch family of proteins is defined by a 50 amino-acid repeat that has been shown to associate with actin [13]. The region containing *KLHL21* in humans has been weakly associated with tumor suppressor activity in a study that selected the gene *CHD5* as the most likely gene with such activity in neuroblastomas [14]. Also *KLHL21*as been presented within a set of genes with descending expression pattern in follicular lymphoma HF4.9 cells after exposure to curcumin [15], but *KLHL21* does not appear often in the cancer literature and by its prevalence in dataset ***A*** it could be considered as a promising candidate to further studies for both, its function and as molecular marker of malignancy. *KLHL21* is classified by Gene Ontology with regard to biological process (BP), as ubiquitin-dependent protein catabolic process, with molecular function

(MF) associated to protein binding and with respect to cellular component (CC) it does not have an available classification (Table S17).

The gene *XAB2*, that encodes the XPA binding protein 2, was found in 9 of the 12 cancer tissues studied and was not detected in bone, liver or muscle cancer tissues. *XAB2* product is a tetratricopeptide repeat protein involved in transcription-coupled DNA repair and transcription by interacting with xeroderma pigmentosum group A protein (XPA), a factor central to nucleotide excision repair pathways [16,17]. Recent results indicated that the *XAB2* complex is a multifunctional factor involved in pre-mRNA splicing, transcription, and transcription-coupled repair [18]. The results presented here indicate that over expression of *XAB2* is a promising trancriptome marker of malignancy. *XAB2* is classified by Gene Ontology with regard to biological process (BP), as associated to blastocyst development, with molecular function (MF) associated to protein binding and with respect to cellular component (CC) as being intracellular (Table S17).

CDT1 encodes the chromatin licensing and DNA replication factor 1 that was found to be highly expressed in 11 of the 12 cancer tissues studied, failing to be detected only in one type of lymphatic cancer, but expressed in the Burkitt lymphoma. Since *CDT1* is required to license DNA for replication in yeast [19] and its deregulation induces chromosomal damage without rereplication and leads to chromosomal instability [20], being down-regulated upon cell cycle exit and over-expressed in cancer-derived cell lines [21] it potentially represents a true cancer marker gene. In this study geminin (*GMNN*), a DNA replication inhibitor, was also found only in cancer tissues with a transcription frequency of 5.32E-05 (data not shown). *CDT1* is classified by Gene Ontology with regard to biological process (BP), as DNA replication checkpoint, with molecular function (MF) associated to DNA binding and with respect to cellular component (CC) it is associated to the nucleaous (Table S17).

TRAF7 is a receptor-associated factor to the Tumor Necrosis Factor (TNF), and belongs to a family of adapter proteins that are involved in signaling by the TNF receptor family and the toll/interleukin-1 receptor (TIR). It has been suggested [22] that *TRAF7* specifically interacts with *MEKK3* and potentiates *MEKK3*-induced AP1 and *CHOP* (C/EBP-homologous protein) activation and induces apoptosis through caspase-dependent pathways. It has been demonstrated that *TRAF7* is an E3

ubiquitin ligase capable of self-ubiquitination [23]. *TRAF7* was found here in 10 of the 12 cancers studied (Table 1), been undetected in bone and lymph cancers. Given the ubiquity and high expression of this gene in cancer tissues it can be considered as a potential molecular marker of malignancy. *TRAF7* is classified by Gene Ontology with regard to biological process (BP), as related to the activation of MAPKKK activity, with molecular function (MF) associated to ubiquitin-protein ligase activity and with respect to cellular component (CC) to ubiquitin ligase complex (Table S17).

The gene with largest relative frequency among cancer tissues was *SILV*, found here in muscle, placenta and skin. This gene encode a product with homology to a chick melanosomal matrix protein and a bovine retinal pigment epithelial protein and it has been suggested that the silver locus product is a melanosomal matrix protein which may contribute to melanogenesis as a structural protein [24]. *SILV* and *MLANA*, other of the genes presented in Table 1 and that appears here only in skin cancer, are regulated by the microphthalmia transcription factor (MITF) [25]. Genes involved in melanin synthesis are commonly used in assays designed to detect melanoma micrometastasis, and some clinical trials defined lymph node-positive disease as detecting the expression of tyrosinase plus the expression of at least one of three other genes (*MLANA*, *SILV*, or *MAGEA3*) [26]. All these four genes were detected in dataset ***A*** expressed only in cancer tissues, but tyrosinase (*TYR*) does not appear as example in Table 1 because it is expressed at an average frequency of 6.34E-05 (data not shown). *SILV* is classified by Gene Ontology with regard to biological process (BP), as melanin biosynthetic process from tyrosine, it does not have an available classification by molecular function (MF) and is associated to extracellular region and with respect to cellular component (CC) (Table S17).

DHRS2, the second most expressed of the genes in Table 1, is present in cancers of liver, lymph, prostate and skin. *DHRS2* is a member of the SDR-type carbonyl reducing enzymes whose physiological roles are insufficiently understood at present [27]. Its antigen has been used in the molecular characterization of human breast tumors [28], where it was found to be up-regulated with an 11.9 fold change in tumor vessel cells. *DHRS2* was also found to be up-regulated after 5-aza-dC treatment in HCT116 colon cancer cell line (3.1 signal log ratio) [29]. *DHRS2* is classified by Gene Ontology with regard to biological process (BP) as related to oxidation reduction, with molecular function (MF) associated to binding and with respect to cellular component (CC) to nucleus (Table S17).

SOX10, found here only in placenta and skin cancers, is a member of the Sox genes that encode transcription factors belonging to the High Mobility Group superfamily and are generally conserved across species and involved in cell lineage determination [30,31]. In particular, *SOX10* has been associated with the maturation and survival of the melanocyte lineage [32]. Mutations in *SOX10* have been associated with neural crest-derived melanocyte deficiency in the Waardenburg syndrome [31]. *PRPS1*, which encodes a phosphoribosyl pyrophosphate synthetase, was found at high expression in 10 of the 12 cancers studied, being not detected only in cancerous muscle tissues and lymphoma of follicular mixed small and large cells. It has been reported that mutations in PRPS1 cause hereditary peripheral neuropathy with hearing loss and optic neuropathy [33], and its transcript has been found to up-regulated in cancer tissues [34] and rat prostate cancer cell lines (8.25 fold change) [35]. *SOX10* is classified by Gene Ontology with regard to biological process (BP), as "Regulation of transcription, DNA-dependent", with molecular function (MF) associated to DNA binding and with respect to cellular component (CC) to nucleus (Table S17).

AIPL1, the gene for aryl hydrocarbon receptor interacting protein-like 1, was found expressed only in cancers of eye and placenta. *AIPL1* is normally present in the developing photoreceptor layer of the human retina and within the photoreceptors of the adult retina, and is thought to be involved in cell cycle progression. Furthermore, mutations in *AIPL1* gene have been found in patients with Leber congenital amaurosis, a form of retinal degeneration [36]. *AIPL1* is classified by Gene Ontology with regard to biological process (BP), as associated to retina homeostasis, with molecular function (MF) associated to farnesylated protein binding and with respect to cellular component (CC) to photoreceptor inner segment (Table S17).

The *GNB3* (G-protein β3 subunit, Table S4) gene was found here only in cancers of eye and placenta, as the case of *AIPL1*. [37] reports that the 825C > T polymorphism in *GNB3* does not appear to be associated with breast cancer risk, but may influence development of metastasis in low-grade tumors. The gene *S100B* (S100 calcium binding protein B) was detected only in placenta and skin cancerous tissues. *S100* proteins are exclusively expressed in vertebrates and are the largest subgroup within the superfamily of EF-hand Ca 2+ -binding proteins [38]. It has been reported that serum levels of *S100B* are a strong prognostic factor for overall and long-term survival on patients with advanced melanoma [39]. *GNB3* is classified by Gene Ontology with regard to biological process (BP), as associated to signal transduction, with molecular function (MF) associated to GTPase activity and with respect to cellular component (CC) to nucleus (Table S17).

The *ZWINT* gene (ZW10 interactor antisense) was expressed in 10 of the 12 cancerous tissues studied, failing to be detected only in placenta and one type of lymphatic cancer, but expressed in the highly related Burkitt lymphoma. It has been demonstrated that *ZWINT-1* specifies localization of Zeste White 10 (ZW10), one of the proteins essential for the fidelity of chromosome segregation, and is essential for mitotic checkpoint signaling [40]. Even more, it has been proved that *ZWINT-1* is an authentic kinetochore protein required for chromosome segregation in humans, and that defects in kinetochore proteins often lead to aneuploidy and cancer [41,42]. Expression of *ZWINT* has been used as molecular classifier for cancer types [43], and has been reported to be responsive to treatment with the aromatase inhibitor letrozole [44]. *ZWINT* has also been used as negative control in assays to find secreted proteins biomarkers of cancer, given that the high expression of *ZWINT* is associated with cancer but its encoded protein is non secreted [45]. Also *ZWINT* has been found to be estrogen-regulated in MCF7 breast cancer cells [46]. *ZWINT* is classified by Gene Ontology with regard to biological process (BP), as associated to mitotic sister chromatid segregation, with molecular function (MF) associated to protein N-terminus binding and with respect to cellular component (CC) to condensed chromosome kinetochore (Table S17).

The gene *SLC45A2*, that encodes the solute carrier family 45 member 2, was found only in skin and testis cancerous tissues. Human *SLC45A2* (also called *MATP*) encodes a protein which is predicted to contain 12 putative transmembrane domains and is presumably located in the melanosomal membrane, probably functioning as a membrane transporter; however its precise role has not been elucidated [47]. Mutations in *SLC45A2* cause oculocutaneous albinism (OCA) type 4 and OCA patients are at a high risk of skin cancer [47]. Also polymorphisms in the promoter of *SLC45A2* have been associated with normal human skin color variation [48]. Our results show that the transcription level of *SLC45A2* could be considered as a biomarker candidate in skin and probably testis cancers. *SLC45A2* is classified by Gene Ontology with regard to biological process (BP), as associated to melanin biosynthetic process from tyrosine, with no molecular function associated to and with respect to cellular component (CC) it is associated to membrane (Table S17).

MEN1 is the gene responsible for the Multiple Endocrine Neoplasia type 1 [49], but the function of its gene product, menin, is uncertain. However [50] report that the menin protein interacts with a putative tumor metastasis suppressor nm23/nucleside diphosphate kinase and this interaction may play important roles in the biological functions of the menin protein, including tumor suppressor activity.

The *MEN1* gene was found in 9 of the 12 cancer tissues studied at relative frequency > 0.0001, and is undetected in bone, muscle and prostate. The P-value for this gene in the Fisher exact test was 1.3e-5 and thus it does not reached statistical significance and is not presented in Table 1.

The gene *SRXN1*, coding for the human Sulfiredoxin 1 homolog (*S. cerevisiae*) was detected in 8 of the 12 cancer tissues studied, being absent in lymph (both types), muscle and testis. Human sulfiredoxin can act as a regulator of the redox-activated thiol switch in cells by catalyzing deglutathionylation of a number of distinct proteins in response to oxidative and/or nitrosative stress, and this suggests that this protein has a central role in redox control with potential implications in cell signaling [51]. *SRXN1* has been found over expressed in many cancer studies and it has also being found showing high differential expression in smoker datasets [52,53], however in this study the P-value in the Fisher exact test (1.3e-5) was not considered to be statistically significant (see Methods) even when is was detected only in cancer tissues with a relative frequency > 0.0001

The transcribed locus *LOC255480* annotated as strongly similar to *NP_036980.1* (ferritin heavy chain), was found expressed in 9 of the 12 cancerous tissues studied, being absent in bone, one kind of lymph and testis cancers. Even when this gene was found with a high frequency of expression (>0.0001), it does not reach significance in this study (P-value=1.3e-5). The associated sequence *NP_036980.1* (gene *Fth1*) is reported to be overexpressed during hepatic tumor development and used as an early marker for hepatocellular carcinoma [54]. High expression of ferritin has also been found to be induced by tumor necrosis factor (TNF), a cytokine which mediates elements of the stress response [55].

The product encoded by *MYC* is a nuclear phosphoprotein with specific transcription factor activity, which plays a role in cell cycle progression, apoptosis and cellular transformation [56]. Mutations, overexpression, rearrangement and translocation of this gene have been associated with a variety of hematopoietic tumors, leukemias and lymphomas, including Burkitt lymphoma [56]. There is evidence to show that alternative translation initiations from an upstream, in-frame non-AUG (CUG) and a downstream AUG start site result in the production of two isoforms with distinct N-termini. The synthesis of non-AUG initiated protein is suppressed in Burkitt's lymphomas, suggesting its importance in the normal function of this gene [56]. The *MYC* transcript was found in 10 of the 12 cancer tissues studied and is absent only from the bone and eye cancer transcriptomes studied, but not reached

significance (P-value=1.2e-5) even when it was only detected in cancerous tissues at relatively high frequency (>0.0001).

Relatively high levels of transcription of the *DHX37* gene was detected in 10 of the 12 cancerous tissues studied, being undetected only in eye and prostate cancers, however the P-value for the hypothesis test of up-regulation of this gene (8.297e-6) is not low enough to be considered significant, and thus is not presented in Table 1. This gene encodes a box protein, characterized by the DEAH conserved motif, and could be a RNA helicase involved in cellular growth and division [57]. It has been reported that species-specific endogenous retroviruses shape the transcriptional network of the human tumor suppressor protein p53, a sequence-specific transcription factor that responds to cellular stresses by coordinating expression of genes involved in cell-cycle arrest, senescence, and apoptosis. p53 regulates genes of diverse biological pathways and is considered a pleiotropic master regulator. Interestingly *DHX37* showed increased p53 occupancy after DNA damage by using ChIP analysis of p53 in *HCT116* cells, implying that this gene is under the control of p53 [58]. Surprisingly, mentions about *DHX37* are almost absent from the cancer literature. However, in one study of the transcriptome of human mammary cell lines expressing different levels of *ERBB2*, a member of the family of transmembrane receptor tyrosine kinases that occurs in 15-30% of primary breast tumors, *DHX37* is shown as part of a list of genes down-regulated in C5.2 cell line, which expresses high levels of erbB2, *versus* the cell line HB4a that expresses basal levels of erbB2 [59]. *DHX37* is also mentioned as responsive to actinomycin D treatment in a study of nucleolar proteome dynamics [57]. Given the ubiquity (10 of 12 cancer tissues) and relative high expression of *DHX37* we think that the role of this gene in cancer deserve further investigation. *DHX37* is classified by Gene Ontology with regard to biological process (BP), as associated to Oxidation reduction, with molecular function (MF) associated to binding and with respect to cellular component (CC) it is associated to nucleus (Table S17).

Ungrouped and complete grouping analysis of dataset *B* (Mouse tissues from the "Cancer Genome Anatomy Project")

Figure 1 B presents the estimated levels of diversity, H_j, and specialization, δ_j, in 5 mouse tissues in normal and cancerous states. In this case four of the five cancerous tissues present a smaller level of specialization than the normal states, however, when comparing the normal tissues with their cancer counterparts, in all cases the normal tissues present a significant larger specialization than the corresponding cancerous states, supporting the hypothesis that in general cancer diminishes the specialization of the tissues. All these differences in specialization were statistically significant ($P<0.01$; Table S6).

From the "ungrouped analysis" of dataset ***B*** we obtained Figure S4 and S5 where we can see that from the 28 possible pair comparisons 23 (82%) give a significant decrease of specialization in the cancer compared with the normal tissues. However this rate varies from tissue to tissue; all cases are in agreement with our hypothesis in lung (6 comparisons), mammary gland (mg; 8 comparisons) and spleen (2 comparisons), while the lower rate is given by the liver (2/6) followed by skin (2/3). In all cases the differences are statistically significant and normally distributed (Table S7), however we need to take into account that many different sources of variation are influencing the estimation of specialization in the tissues; within these we can mention the genetic variation among the strains used, age and diet of the animals, specific type of cancer, state of development of the tumor, sequencing method and sample size obtained (number of tags in each library). To block the sources of variation, and test only the level of specialization in each organ depending of its state (normal or cancerous), we performed the "grouping analysis". In doing this we are loosing information only of the particular differences between the libraries of the same tissue and state, but that information is not relevant here because it represent either random noise or variation given by factors that are non pertinent to this study. The result of this analysis was presented in Figure 1 B, where we can see that all normal organs have a larger estimated specialization (δ_j) than the corresponding cancerous state. We can also notice that the 95% approximate confidence intervals for the parameters perfectly separate all sets in specific regions of the diversity by specialization space. The lengths of the confidence intervals depend upon the number of tags in each dataset, and this is reflected in Figure 1 B.

To evaluate the significance of the differences in specialization observed in Figure 1 B we obtained an approximate 99% confidence interval for those differences using the B = 2000 bootstrap replicates. The result of this procedure is presented in Table S7. As can be observed in Table S7 none of the 5 confidence intervals contain the zero value, and thus all differences in specialization are statistically

significant (P<0.01). In contrast with the analysis performed with individual libraries (Figure S4 and FigureS5), all comparisons agree with our hypothesis.

The results of the "complete grouping" for dataset B are shown in Figure S6, were we can see a marked drop in specialization in the combined transcriptome of cancerous tissues when compared with the combined transcriptome of the normal state. The estimated mean difference between specialization in the normal and cancer sets was 0.1101 with standard deviation of 0.0015 and 99% approximate confidence limits equal to 0.1063 and 0.1140 (lower and upper limits respectively obtained by the bootstrap percentile method); thus this difference is statistically significant.

Confidence intervals for specialization in Dataset *C* (Human normal and tumor tissues from the "Human Transcriptome Map")

Figure1 C presents the scatter plot of diversity versus specialization for normal and tumor transcriptomes from a complex mixture of tissues. The analysis was performed separately for the loci in each chromosome and then in group (All Chromosomes). For 21 of the 23 chromosomes there is a sharp decrease in the specialization of the tumor compared with the normal tissues, the exceptions being chromosomes Y and 18. The increase in specialization for the transcriptome of genes belonging to chromosome Y was not statistically significant, but it was for the case of chromosome 18 (Table S8). As result of the global analysis (All Chromosomes in Figure1 C), there is a global loss of specialization in the transcriptome of tumors when compared with the normal tissues, and this difference is statistically significant (P<0.01 see confidence limits in Table S8).

For clarity the confidence intervals for the values of diversity and specialization are not presented in Figure 2 but are separately plotted in Figs. S7, S8, S9 and S10, that amplify the colored boxes presented in Figure 2 and represent groups of chromosomes with similar diversity.

From figures S7 to S10 we can see that all, except chromosomes Y and 18, present a reduction in specialization in the tumor compared with the normal tissues. In all cases this reduction is statistically significant (P<0.01), as can be seen in Table S8 that presents the 99% confidence intervals for the differences in specialization in each chromosome and the set of all the chromosomes. The increase in specialization from normal to tumor tissues observed in chromosome 18 is also significant, but the corresponding increase observed in the chromosome Y is not statistically significant (Table S8). Interestingly chromosome 18 contains several tumor suppressor genes including *DDC*, *DPC4* and *JV18-1/MADR2* [60]. Overall 22 of 23 (96%) of the significant tests performed by chromosome supports the hypothesis that the specialization level of the transcriptome falls in tumors. It is important

to underline that these tests are independent, because they are using data from the different sets of loci present in each chromosome. Also the test performed for the difference in specialization between all loci (All chromosomes in Table S8) strongly endorses the hypothesis that cancer reduces the specialization of transcriptomes.

Given that the libraries downloaded for dataset ***C*** were composed of a mixtures of normal or tumor tissues, only the "complete group" analysis was performed here, but future work can include the data-mining of individual libraries to evaluate the specialization and diversity as well as gene specificity in particular tissues and specific types of cancer. Table S9 presents the number of tags and loci per chromosome in dataset ***C***. From this table we can appreciate that the number of loci per chromosome is highly variable and approximately correlated with chromosome size.

Table S10 presents examples of the most transcribed genes that were expressed exclusively in tumors. From Table S10 we can note that the genes associated with "carboxypeptidase B1" is the one with highest exclusively expression in tumor tissues. This gene has been repeatedly reported in association with cancer [61]. As tables 1, Table S10 only exemplify the rich possibilities of data mining that are possible by using gene specificities and target specificities in cancer research.

Dataset *D* – Human microarray data of normal and precancerous states in breast tissue

Figure1 ***D*** presents the estimated values of diversity and specialization for eight paired samples of transcriptomes. The data are paired by patient of origin and were obtained from microdissected samples of normal breast tissue (terminal duct lobular units; TDLU) or hyperplastic enlarged lobular units (HELUs) that are the earliest histologically identifiable potential precursor of breast cancer [62]. From Figure1 ***D*** we can see that in seven of the eight patients, there is a diminution of the transcriptome specialization in the transcriptome of pre-cancerous state (HELUs) when compared with the normal tissue (TDLUs). This diminution in specialization is probably due to the changes observed by [62] in the transcriptome that suggest that HELUs evolve from TDLUs primarily by reactivation of pathways involved in embryonic development and suppression of terminal differentiation. In the analysis of dataset ***D*** it was not possible to obtain the confidence intervals for the parameters, neither to test the significance of the differences, given that the data are indirect and continuous measures of gene expression without true replicates.

Dissecting differences in transcriptome specialization

Transcriptome specialization is defined [2] as

$$\delta_j = \sum_{i=1}^{g} p_{ij} S_i \qquad [1.]$$

where S_i is gene specificity (see Mathematical Addendum) thus the difference in specialization between two transcriptomes, say δ_j - δ_k, can be written as

$$\delta_j - \delta_k = \sum_{i=1}^{g} (p_{ij} - p_{ik}) S_i \qquad [2.]$$

The elements of the sum in [2.] that are relevant are the ones for which $p_{ij} - p_{ik} \neq 0$, that is, the genes that present a distinct level of relative expression in the transcriptomes. If $p_{ij} - p_{ik} > 0$, then the gene i is over-expressed in transcriptome j, and if $p_{ij} - p_{ik} < 0$ the reverse is true; i.e., the gene i is over-expressed in transcriptome k. Assume that the sub-index j corresponds to a normal transcriptome and k to the corresponding cancer transcriptome. Now let us re-index the variables $\{p_{ij}, p_{ik}\}$ in such a way that a new sub-index r will point to the values for which $p_{ij} - p_{ik} > 0$ and a the sub-index v will denote the values for which $p_{ij} - p_{ik} < 0$. Then [2.] above can be written as

$$\delta_j - \delta_k = \sum_{r=1}^{n} (p_{rj} - p_{rk}) S_r + \sum_{v=1}^{c} (p_{vj} - p_{vk}) S_v \qquad [3.]$$

where the first sum is over the genes over-expressed in the normal tissue (and thus always gives a positive value) and the second sum is over the genes over-expressed in cancer (and thus it always gives a negative value) and the total number of over-expressed genes is $n + c \leq g$.

Then the hypothesis that cancer diminishes the specialization of the tissues, say $\delta_j - \delta_k > 0$, is equivalent to the hypothesis that

$$\sum_{r=1}^{n} (p_{rj} - p_{rk}) S_r > -\sum_{v=1}^{c} (p_{vj} - p_{vk}) S_v \qquad [4.]$$

To make the notation more compact, let us denote de differences in expression as $d_i = p_{ij} - p_{ik}$ and then the values of $d_r;\ r = 1, 2, \dots n$, denote the (positive) differences in expression of the genes over-expressed in the normal tissue, while $d_v;\ v = 1, 2, \dots c$, denote the (negative) differences in expression of the genes over-expressed in cancer. Thus finally the hypothesis can be re-written as

$$\sum_{r=1}^{n} d_r S_r > -\sum_{v=1}^{c} d_v S_v \qquad [5.]$$

We can try to have a better understanding of the phenomenon by inspecting the relations that exist between the number of genes over-expressed in each category (n *versus* c), as well as the mean values of the over-expression deviations, say

$$\bar{d}_r = \frac{1}{n} \sum_{r=1}^{n} d_r \,,\ \bar{d}_v = \frac{1}{c} \sum_{v=1}^{c} d_v \qquad [6.]$$

and the means of the specificity coefficients for these kinds of genes, say

$$\bar{S}_r = \frac{1}{n}\sum_{r=1}^{n} S_r \,,\ \bar{S}_v = \frac{1}{c}\sum_{v=1}^{c} S_v \,. \qquad [7.]$$

It is important to underline that these indexes must be calculated separately for each pair of organs (*j* and *k*) that want to be examined. We can examine for example if it is the case that the average of specificity for the genes over-expressed in normal tissues, $\bar{S}_r$, is larger than the corresponding mean in the genes over-expressed in cancer, $\bar{S}_v$.

For datasets ***A*** and ***B*** and within them for each comparison between a cancerous and normal state, we present graphs of S_i *versus* d_i, that permit a visual appreciation of the grounds for the difference in specialization, as well as tables containing the values of *n, c*, $\bar{S}_r$, $\bar{S}_v$, $\bar{d}_r$ and $\bar{d}_v$. We also performed t-test for the hypotheses than the means of specificity are equal, say H0: $\bar{S}_r = \bar{S}_v$ and also for the hypotheses than the mean of the deviations are equal, say H0: $\bar{d}_r = -\bar{d}_v$.

The influence of a given gene over the change in specialization in a tissue can be measured as the absolute value of the difference in expression times the specialization of the gene. Here we denote such influence as ι_i given by

$$\iota_i = \left| d_i S_i \right| \qquad [8.]$$

A gain in comprehension of the nature of the change in specialization can be obtained by examining the largest values of ι_i for a set of genes in each comparison.

Results of dissecting differences in transcriptome specialization

We have seen that there is a significant change in the specialization of the transcriptome when comparing normal and cancerous tissues. Here we present the results of dissecting that change by plotting and testing the differential expression of genes in normal and cancerous tissues and its specificity in datasets ***A***, ***B*** and for chromosome 18 in dataset ***C***.

Figure S11 presents, as example, the scatter plots for the differences in expression: Relative frequency in Normal – Relative frequency in Cancer = $d_i = p_{ij} - p_{ik}$ *versus* values of gene specificities (S_i) for the liver (dataset ***A***). From Figure S11 we can see that there is a tendency of over-expression of highly specific genes in the normal tissue. This tendency is especially noticeable in liver (Figure S11), prostate and skin (data not shown). This implies that the change in specialization of the tissues can generally be explained by the down regulation or shoot-down of highly specific genes during the process of carcinogenesis.

From Table S11 we can see that in all cases the average specificity (S_i) of the over-expressed genes is highly significant (P-value ≤ 0.00005), except in the case of kidney where the significance is a bit lower (P-value = 0.014). In the majority of the cases, the average specificity of the genes over-expressed in the normal tissues, $\bar{S}_r$, is larger than the average specificity of the genes over-expressed in the cancer tissues, $\bar{S}_v$, the exceptions being "Skin", "Lymphr" and "Eye" where the reverse is true. This means that in general more specific genes are turned down when going from the normal to the cancerous state, and this major factor imply the diminishing of tissue specialization. In the case of "Skin" the average specificity of the genes over-expressed in cancer (2.44) is much larger than the specificity of the genes over-expressed in the normal tissue (2.01), but the average frequency of change in the set over-expressed in the normal tissue ($\bar{d}_r$; value 0.000202) is much larger than the average frequency of change in the set over-expressed in the cancer tissue ($\bar{d}_v$; value 0.000064), and thus the net effect is a drop in the specialization of the cancerous tissue. The same happens in the case of "Lymphr". The case of "Eye" where the specialization of the tissue appears higher in the cancer tissue than in the normal deserves further study. In this case the average specificity of the genes over-expressed in the normal tissue (1.74) is significantly lower than the average specificity of the genes over-expressed in the cancer tissue (2.11), and the average change of expression in the genes over-expressed in the normal tissue ($\bar{d}_r$; value 0.000215) is 2.4 times larger than the average change of expression in the genes over-expressed in the cancer tissue ($\bar{d}_v$; value 0.000089). From Table S1 we can notice that the number of gene tags in the normal eye tissue (10,679) represents just 25% of the number of tags present in the eye cancer libraries (42,029), thus it is possible that many eye-specific genes are not estimated (have a sample frequency of zero), just because the sample size of the normal library is too small. This has a double effect, at one hand it decreases the number of "eye-specific" genes estimated, and on the other it increases the specificity of some "eye-specific" genes that are present in the eye cancer libraries, without been exclusive of cancer, and thus artificially increases the specialization of the eye cancer transcriptome.

From the last row in Table S11 we can see that the average number of genes over-expressed in cancer (5,981) is 1.78 times larger than the average number of genes over-expressed in the corresponding normal tissues (3,359); this means that in general a very large set of genes is de-regulated in cancer and that cancerous tissues tend to over-express many genes that are expressed at lower or null frequencies in normal tissues. However, the average specificity of the genes over-expressed in normal tissues (2.22) is larger than the average specificity of genes over-expressed in cancer (2.03). Also the average change in the genes over-expressed in normal tissues (0.000244) is 1.79 times larger than the average change

in the genes over-expressed in cancer tissues (0.000136), and thus this explains the general drop of specialization of the cancer tissues when compared with their normal counterparts.

Table S12 presents the ten genes with most influence in the change of specialization for each human organ studied in dataset ***A*** (see equation 8.).

From Table S12 we can see that the majority (104/120; 87%) of the most influential genes are genes over-expressed in the normal tissue with regard to the cancerous state, i.e., genes for which $p_{ij}-p_{ik}>0$. In eight cases (marked in bold in the column S_i of Table S12) the most influential genes reach the largest possible value for gene specificity by being expressed only in one of the tissues, say, $max\{S_i\} = log_2(24) = 4.5850$ These genes are *CHAD* (Chondroadherin; Bone), *UMOD* (Uromodulin; Kidney), *CYP19A1* and *CSH1* (Cytochrome and Chorionic somatomammotropin hormone 1 respectively in Placenta) and *PRM1* (Protamine 1), *PRM2* (Protamine 2), *SPATA4* (Spermatogenesis associated 4) and an unidentified cDNA clone (*C14orf37*), all four of them in Testis. It is also worth noting that within the most influential genes in Table S13 there is a gene with low specificity ($S_i \leq 0.6431$) that corresponds to the eukaryotic translation elongation factor 1 alpha 1 (*LOC286184* and *HNRPA1P5*). This gene is over-expressed in cancerous tissues of Lymphr, Testis and Eye, and it reaches the high level of influence, ι_i, not by its specificity but for its large over-expression in cancer tissues ($p_{ij}-p_{ik}<0$). The five genes with highest influence (larger ι_i) in Table S12 are *ALB* (Albumin; Liver), FGG (Fibrinogen gamma chain; Liver), *MSMB* (Microseminoprotein; Prostate), *RAB8A* (Metastasis associated lung adenocarcinoma transcript 1; Prostate) and *SERPINA1* (Serpin peptidase inhibitor, clade A; Liver). All these four genes have a relatively large specificity, except *RAB8A* with S_i=2.0606. This gene, a transcript originally associated with lung carcinoma, is the most frequent gene in Table S12, being repressed ($p_{ij}-p_{ik}>0$) in five types of cancers (Prostate, Lung, Kidney, Muscle and Testis). Distinct kinds of keratine genes (numbers 1, 5, 10, 14, 17, 18, 19 and 79) are present in Table S12 in cancers of eye and skin and always over-expressed in cancer ($p_{ij}-p_{ik}>0$).

The cases presented in Table S12, and before in Table 1 exemplify the richness of data-mining information that the application of the information tools can bring to the study of human cancer.

Figure S12 presents a scatter plot for the differences in expression: Relative frequency in Normal – Relative frequency in Cancer = $d_i = p_{ij} - p_{ik}$ *versus* values of gene specificities (S_i) for the 5 mouse liver (dataset ***B***). From Figure S12 we can see that there is a tendency of over-expression of highly specific genes in normal liver. The same tendency was observed in the other mouse organs (data not shown). As in the case of dataset ***A***, this implies that the change in specialization of the tissues can be mainly explained by the down regulation of highly specific genes during the process of carcinogenesis.

Table S13 presents the statistics for the genes over-expressed in normal (N) and cancerous (C) tissues in the mouse dataset (***B***).

From Table S13 we can see that the average specialization of over-expressed genes is significantly distinct for all five organs, and for four of them the average specialization of genes over-expressed in normal tissues is larger than in the cancerous counterparts, the exception being mammary gland (MG). In the case of MG we can see that the average of over-expression of genes in the normal organ ($\bar{d}_r$) is more than 10 times larger than the corresponding average in cancerous tissues ($\bar{d}_v$); this partially explain the drop of specialization in the cancerous MG with regard to its normal counterpart. Table S14 present the ten most influential genes that explain the drop of specialization of the cancerous tissues. From Table S14, we can see that in the group of the ten most influential genes in MG we have four specific genes clearly associated with milk production which large drop in expression in cancerous tissues explain the drop of specialization in this tissue.

From Table S14 we can observe that for each organ there are examples of highly organ-specific genes (maximum value of S_i) that are switch-off in the cancerous state, explaining the drop of specialization of the tissue. In this case the maximum value of gene specificity is $max\{S_i\} = log_2(10) = 3.3219$, that is reached by ten genes (values of S_i marked in black in Table S14). This kind of genes are scattered among the five studied organs, being three of them of liver (members of the major urinary protein family), three in lung (two members of the surfactant associated protein and one chemokine ligand), one unidentified locus in mammary gland (MG) and *Ms4a1* in spleen; all of them over-expressed in normal tissues. The exception to the specific genes is a tyrosinase and a MAS-related GPR from skin, which is over-expressed in the cancerous tissues. For the 50 genes presented in Table S14, 37 (71%) are over-expressed in normal tissues, confirming that the drop of specialization in cancer tissues is mainly due to the reduction of expression of highly specific genes. In Table S14 the three genes with the largest influence in specialization drop are members of the Casein family (*Csn2*, *Csn1s2b* and *Csn3*) in mammary gland (MG) that are shut-down from the normal to the cancerous state of this organ.

In dataset ***C*** (Human Transcriptome Map) the only chromosome that does not satisfy the hypothesis of decreasing specialization in tumor tissues is chromosome 18. This anomaly can be due to random variation, given that when analyzing all the loci together (see row “All chromosomes” in Table S8 and Figure S10) there is a significant drop of specialization in the tumor tissues when compared with the normal counterpart. It is also worth noting that the number of tags for the normal tissues in chromosome 18: 92,302 is less than half than the tags obtained for normal tissues: 203,690 (Table S3). This sample size difference affects the estimation of low expression loci, i.e., genes with an equal but

low expression in both tumor and normal tissues will be detected only in tumor tissues and thus will be wrongly classified as "specific" to tumor, artificially inflating the specialization of the tumor tissues. Other possibility is that loci that affect functions gained by the tumoral cells would be clustered into the chromosome 18, making then the tumor tissues more specialized than the normal one when looking only to genes of this chromosome. To investigate this possibility we carried out an influence analysis on the chromosome 18. Table S15 presents the results of the influence analysis carried out on chromosome 18.

From Table S15 we can see that for chromosome 18 (dataset ***C***) the average specialization of the genes over-expressed in the normal tissues is significantly smaller than the corresponding value in the tumor tissues, which explains the increase in specialization of the tumor tissues, without ruling out that this could be an effect of the smaller sample size (number of tags) in the normal tissues. The average difference in over-expressed genes is not significantly different between normal and tumor tissues.

To further understand the increase of specialization in chromosome 18 (dataset ***C***) we detected the ten loci with more influence in the phenomenon. These results are presented in Table S16.

In Table S16 we see that one of the most influential genes is a mitogen-activated protein kinase 4 (*MAPK4*). *MAPK4* belong to a family of serine/threonine-specific protein kinases that respond to extracellular stimuli (mitogens) and regulate various cellular activities, such as gene expression, mitosis, differentiation, and cell survival/apoptosis [63]. This locus is expressed 2.77 times more in tumor than normal tissues. Further study is needed to test the hypothesis that loci involved with the functions gained by tumoral cells are more frequent in chromosome 18 than in other chromosomes.

Analysis of dataset *D* (microarray data for human normal and precancerous tissues)

Figure S13 shows that in seven of the eight patients studied (88%) there is a decrease in the estimated specialization of the precancerous tissues. Given that there were not replicates of the data, it was impossible to calculate the confidence intervals for H_j (Diversity) and δ_j (Specialization), and thus there is no way to judge the statistical significance of these results. Also, as mentioned before, the estimated rank of variation of diversity and specialization in the human transcriptome is much smaller when using microarrays than when counting gene tags [2], and this possibly distort the expression of genes with extreme expressions.

General considerations

From the evidence presented here we can advance the hypothesis that in general cancer diminishes the specialization of the affected tissues. This implies that even when cancer tissues gain a set of specialized functions, mainly related with cell cycle, angiogenesis and capability to form metastasis, the deregulation of sets of genes with tissue-specific function usually inclines the specialization balance to the side of specialization loss for cancer tissues.

In the practical side, the application of gene specificity and target specificity provides a powerful tool for the data mining of the many studies already performed comparing normal with cancerous tissues. We have exemplified how this method has the capability to recover not only the genes well known to be associated with cancer, but also less well studied genes that are highly and generally up-regulated during neoplasias and can help in the understanding of the process and, hopefully, signal to new possibilities of intervention by drug or genomic medicine. The same tools can be used to pinpoint genes down-regulated in cancer or genes that remain constant during the phenomenon, and thus can be used as controls.

Mathematical Addendum

Information properties of transcriptomes

The formulas for H_j, S_i and δ_j were presented in [2], and are also presented in the Methods section. As an extension we define here the target specificity of the *i*-th gene about the *j*-th tissue as:

$$TS_{ij} = \frac{p_{ij}}{p_i t} S_i .$$

The idea behind TS is to decompose S_i into additive components due to each sampled tissue.

Meaning of the coefficient in TS

$$\frac{p_{ij}}{p_i t} = p_{(j|i)},$$

i.e. it is the probability of the *j*-th tissue given the *i*-th gene.

Properties of TS

1. It measures the specificity of the *i*-th gene for the *j*-th tissue

2. $\sum_{j=1}^{t} TS_{ij} = S_i$

3. $0 \le TS_{ij} \le S_i$

4. $TS_{ij} = S_i$ if and only if $\frac{p_{ij}}{p_i t} = 1$

5. **If** $TS_{ij} = S_i$ **then** $S_i = \log_2 t$

Consequences of the properties of TS

1. The minimum value of *TS* is zero, and it is reached when either the gene specificity S_i is zero, or when the gene is absolutely not transcribed in the target tissue
2. The maximum value of *TS* is $log_2 t$ and takes place when a gene is transcribed in the target tissue but untranscribed in the remaining sampled tissues
3. When *TS* for a given gene is maximum in a tissue, then it is zero in the remaining sampled tissues

Source Code for the R function to calculate informational properties of the transcriptomes

```
marv <- function(x) {

# Octavio Martínez de la Vega
# omartine@ira.cinvestav.mx
# June 2009
# Version 1.0

# This software is distributed under the terms of the GNU GENERAL
# PUBLIC LICENSE Version 2, June 1991.
# This is free software and comes with ABSOLUTELY NO WARRANTY.

# marv
# Implements the methods presented in:
# Martínez O, Reyes-Valdés H (2008)
# Defining diversity, specialization, and gene specificity in transcriptomes
# through information theory.
# Proceedings of the National Academy of Sciences 105: 9709-9714.

# x must be a data.frame with
#       columns = organs or transcriptome conditions
#       rows = genes
# The data must be counts of the number of gene tags
# in each organ or normalized microarray lectures.

x.g <- length(x[,1]) # i = gene; 1, 2, ... g.
```

```
x.t <- length(x[1,]) # j = organ; 1, 2, ... t.
h.max <- log2(x.t)
x.sum.tags <- apply(x,2,sum,na.rm=T)
x.p <- data.frame(x[,1]/x.sum.tags[1])
for (i in 2:x.t){
x.p <- data.frame(x.p,x[,i]/x.sum.tags[i])
}
attributes(x.p)$names <- paste("p_", attributes(x)$names, sep = "")
attributes(x.p)$row.names <- paste("p_",attributes(x)$row.names,sep="")
p.gen <- apply(x.p,1,mean,na.rm=T)
h.org <- apply(-x.p*log2(x.p),2,sum,na.rm=T)
hr.org <- apply(-x.p*log2(p.gen),2,sum,na.rm=T)
dj.org <- hr.org - h.org
Si <- (apply((x.p/p.gen)*log2(x.p/p.gen),1,sum,na.rm=T))/x.t # x.p/p.gen = Matrix
of pij/pi
delta.organ <- apply(x.p*Si,2,sum,na.rm=T)

# Results for gene
# Description
# "pi" - Mean relative frequency for each gene [Eq. 2]
# "Si" - Gene specificity [Eq. 3]
gen.res <- data.frame(p.gen,Si)
attributes(gen.res)$names <- c("pi","Si")

# Results per organ
# Description
# "tags" - Total sum of the tags per organ
# "Hj" - Organ diversity [Eq. 1]
# "Delta-j" - Average gene specificity (specialization) [Eq. 4]
# "Dj" - Divergence [Eq.6]

x.res <- data.frame(x.sum.tags, h.org, delta.organ, dj.org)
attributes(x.res)$names <- c("tags", "Hj", "Deltaj","Dj")

# Result for system
# Vector with:
# Genes - Total number of genes.
# Organs - Total number of organs
# H - Diversity of the system [Eq. 2 but with pi = mean frequency of genes]
temp <- c(x.g, x.t, sum(-p.gen*log2(p.gen),na.rm=T),log2(x.g))
attributes(temp)$names <- c("Genes","Organs","H","Hmax")

# Final object
res <- list(x.res,gen.res,x.p,temp)
attributes(res)$names <- c("organ","gene","freq","system")
res
}
```

Supporting References

1. Hanahan D, Weinberg RA (2000) The Hallmarks of Cancer. Cell 100: 57-70.

2. Martínez O, Reyes-Valdés H (2008) Defining diversity, specialization, and gene specificity in transcriptomes through information theory. Proceedings of the National Academy of Sciences 105: 9709–9714.

3. Sandberg R, Ernberg I (2005) Assessment of tumor characteristic gene expression in cell lines using a tissue similarity index (TSI). Proceedings of the National Academy of Sciences of the United States of America 102: 2052-2057.

4. Hirokawa N, Takemura R (2004) Kinesin superfamily proteins and their various functions and dynamics. Experimental Cell Research 301: 50-59.

5. Saito N, Okada Y, Noda Y, Kinoshita Y, Kondo S, et al. (1997) KIFC2 Is a Novel Neuron-Specific C-Terminal Type Kinesin Superfamily Motor for Dendritic Transport of Multivesicular Body-Like Organelles. Neuron 18: 425-438.

6. Nath S, Bananis E, Sarkar S, Stockert RJ, Sperry AO, et al. (2007) Kif5B and Kifc1 Interact and Are Required for Motility and Fission of Early Endocytic Vesicles in Mouse Liver. Mol Biol Cell 18: 1839-1849.

7. Dairkee SH, Ji Y, Ben Y, Moore DH, Meng Z, et al. (2004) A molecular 'signature' of primary breast cancer cultures; patterns resembling tumor tissue. BMC Genomics 5: 47.

8. Koller E, Propp S, Zhang H, Zhao C, Xiao X, et al. (2006) Use of a Chemically Modified Antisense Oligonucleotide Library to Identify and Validate Eg5 (Kinesin-Like 1) as a Target for Antineoplastic Drug Development. Cancer Res 66: 2059-2066.

9. Krishnan KJ, Reeve AK, Samuels DC, Chinnery PF, Blackwood JK, et al. (2008) What causes mitochondrial DNA deletions in human cells? Nat Genet 40: 275-279.

10. Spelbrink JN, Li FY, Tiranti V, Nikali K, Yuan QP, et al. (2001) Human mitochondrial DNA deletions associated with mutations in the gene encoding Twinkle, a phage T7 gene 4-like protein localized in mitochondria. Nature Genetics 28: 223-231.

11. Moraes CT (2001) A helicase is born. Nature Genetics 28: 200-201.

12. Kelso RJ, Hudson AM, Cooley L (2002) Drosophila Kelch regulates actin organization via Src64-dependent tyrosine phosphorylation. J Cell Biol 156: 703-713.

13. Kim IF, Mohammadi E, Huang RCC (1999) Isolation and characterization of IPP, a novel human gene encoding an actin-binding, kelch-like protein. Gene 228: 73-83.

14. Fujita T, Igarashi J, Okawa ER, Gotoh T, Manne J, et al. (2008) CHD5, a Tumor Suppressor Gene Deleted From 1p36.31 in Neuroblastomas. J Natl Cancer Inst 100: 940-949.

15. Skommer J, Wlodkowic D, Pelkonen J (2007) Gene-expression profiling during curcumin-induced apoptosis reveals downregulation of CXCR4. Experimental Hematology 35: 84-95.

16. Nakatsu Y, Asahina H, Citterio E, Rademakers S, Vermeulen W, et al. (2000) XAB2, a Novel Tetratricopeptide Repeat Protein Involved in Transcription-coupled DNA Repair and Transcription. J Biol Chem 275: 34931-34937.

17. Nitta M, Saijo M, Kodo N, Matsuda T, Nakatsu Y, et al. (2000) A novel cytoplasmic GTPase XAB1 interacts with DNA repair protein XPA. Nucl Acids Res 28: 4212-4218.

18. Kuraoka I, Ito S, Wada T, Hayashida M, Lee L, et al. (2008) Isolation of XAB2 Complex Involved in Pre-mRNA Splicing, Transcription, and Transcription-coupled Repair. J Biol Chem 283: 940-950.

19. Nishitani H, Lygerou Z, Nishimoto T, Nurse P (2000) The Cdt1 protein is required to license DNA for replication in fission yeast. Nature 404: 625-628.

20. Tatsumi Y, Sugimoto N, Yugawa T, Narisawa-Saito M, Kiyono T, et al. (2006) Deregulation of Cdt1 induces chromosomal damage without rereplication and leads to chromosomal instability. J Cell Sci 119: 3128-3140.

21. Xouri G, Lygerou Z, Nishitani H, Pachnis V, Nurse P, et al. (2004) Cdt1 and geminin are down-regulated upon cell cycle exit and are over-expressed in cancer-derived cell lines. European Journal of Biochemistry 271: 3368-3378.

22. Xu L-G, Li L-Y, Shu H-B (2004) TRAF7 Potentiates MEKK3-induced AP1 and CHOP Activation and Induces Apoptosis. J Biol Chem 279: 17278-17282.

23. Bouwmeester T, Bauch A, Ruffner H, Angrand PO, Bergamini G, et al. (2004) A physical and functional map of the human TNF-alpha/NF-kappa B signal transduction pathway. Nature Cell Biology 6: 97-105.

24. Kobayashi T, Urabe K, Orlow SJ, Higashi K, Imokawa G, et al. (1994) The Pmel 17/silver locus protein. Characterization and investigation of its melanogenic function. J Biol Chem 269: 29198-29205.

25. Bödding M (2007) TRP proteins and cancer. Cellular Signalling 19: 617-624.

26. Tracey B. Lewis JERRBBMKBWESSALRDNCTWLPPS (2005) Molecular classification of melanoma using real-time quantitative reverse transcriptase-polymerase chain reaction. Cancer 104: 1678-1686.

27. Oppermann U (2007) Carbonyl Reductases: The Complex Relationships of Mammalian Carbonyl- and Quinone-Reducing Enzymes and Their Role in Physiology. Annual Review of Pharmacology and Toxicology 47: 293-322.

28. Bhati R, Patterson C, Livasy CA, Fan C, Ketelsen D, et al. (2008) Molecular Characterization of Human Breast Tumor Vascular Cells. Am J Pathol 172: 1381-1390.

29. Eriko Okochi-Takada KNMWAMSITYTU (2006) Silencing of the <I>UCHL1</I> gene in human colorectal and ovarian cancers. International Journal of Cancer 119: 1338-1344.

30. Dong C, Wilhelm D, Koopman P (2004) Sox genes and cancer. Cytogenet Genome Res 105: 442-447.
31. Potterf SB, Furumura M, Dunn KJ, Arnheiter H, Pavan WJ (2000) Transcription factor hierarchy in Waardenburg syndrome: regulation of MITF expression by SOX10 and PAX3. Human Genetics 107: 1-6.
32. Garraway LA, Sellers WR (2006) Lineage dependency and lineage-survival oncogenes in human cancer. Nat Rev Cancer 6: 593-602.
33. Kim H-J, Sohn K-M, Shy ME, Krajewski KM, Hwang M, et al. (2007) Mutations in PRPS1, Which Encodes the Phosphoribosyl Pyrophosphate Synthetase Enzyme Critical for Nucleotide Biosynthesis, Cause Hereditary Peripheral Neuropathy with Hearing Loss and Optic Neuropathy (CMTX5). The American Journal of Human Genetics 81: 552-558.
34. Mutter GL, Baak JPA, Fitzgerald JT, Gray R, Neuberg D, et al. (2001) Global Expression Changes of Constitutive and Hormonally Regulated Genes during Endometrial Neoplastic Transformation. Gynecologic Oncology 83: 177-185.
35. Reyes I, Tiwari R, Geliebter J, Reyes N (2007) DNA microarray analysis reveals metastasis-associated genes in rat prostate cancer cell lines. Biomédica 27: 190-203.
36. Akey DT, Zhu X, Dyer M, Li A, Sorensen A, et al. (2002) The inherited blindness associated protein AIPL1 interacts with the cell cycle regulator protein NUB1. Hum Mol Genet 11: 2723-2733.
37. Krippl P, Langsenlehner U, Renner W, Yazdani-Biuki B, Wolf G, et al. (2004) The 825C> T polymorphism of the G-protein beta-3 subunit gene (GNB3) and breast cancer. Cancer Letters 206: 59-62.
38. Heizmann CW, Ackermann GE, Galichet A (2007) Pathologies involving the S100 proteins and RAGE. Calicum Signaling and Disease, Springer Verlag.
39. Egberts F, Pollex A, Egberts JH, Kaehler KC, Weichenthal M, et al. (2008) Long-Term Survival Analysis in Metastatic Melanoma: Serum S100B Is an Independent Prognostic Marker and Superior to LDH. Onkology 31: 380-384.
40. Wang H, Hu X, Ding X, Dou Z, Yang Z, et al. (2004) Human Zwint-1 Specifies Localization of Zeste White 10 to Kinetochores and Is Essential for Mitotic Checkpoint Signaling. J Biol Chem 279: 54590-54598.
41. Obuse C, Iwasaki O, Kiyomitsu T, Goshima G, Toyoda Y, et al. (2004) A conserved Mis12 centromere complex is linked to heterochromatic HP1 and outer kinetochore protein Zwint-1. Nature Cell Biology 6: 1135-1141.

42. Weaver BAA, Cleveland DW (2006) Does aneuploidy cause cancer? Current Opinion in Cell Biology 18: 658-667.
43. Teschendorff AE, Naderi A, Barbosa-Morais NL, Caldas C (2006) PACK: Profile Analysis using Clustering and Kurtosis to find molecular classifiers in cancer. Bioinformatics 22: 2269-2275.
44. Miller WR, Larionov AA, Renshaw L, Anderson TJ, White S, et al. (2007) Changes in breast cancer transcriptional profiles after treatment with the aromatase inhibitor, letrozole. Pharmacogenetics and Genomics 17: 813-826.
45. Klee EW, Finlay JA, McDonald C, Attewell JR, Hebrink D, et al. (2006) Bioinformatics Methods for Prioritizing Serum Biomarker Candidates. Clin Chem 52: 2162-2164.
46. Buterin T, Koch C, Naegeli H (2006) Convergent transcriptional profiles induced by endogenous estrogen and distinct xenoestrogens in breast cancer cells. Carcinogenesis 27: 1567-1578.
47. Sengupta M, Chaki M, Arti N, Ray K (2007) SLC45A2 variations in Indian oculocutaneous albinism patients. Mol Vis 13: 1406-1411.
48. Justin Graf JVIHAvD (2007) Promoter polymorphisms in the <I>MATP</I> (<I>SLC45A2</I>) gene are associated with normal human skin color variation. Human Mutation 28: 710-717.
49. Lemmens I, Van de Ven WJ, Kas K, Zhang CX, Giraud S, et al. (1997) Identification of the multiple endocrine neoplasia type 1 (MEN1) gene. The European Consortium on MEN1. Hum Mol Genet 6: 1177-1183.
50. Ohkura N, Kishi M, Tsukada T, Yamaguchi K (2001) Menin, a Gene Product Responsible for Multiple Endocrine Neoplasia Type 1, Interacts with the Putative Tumor Metastasis Suppressor nm23. Biochemical and Biophysical Research Communications 282: 1206-1210.
51. Findlay VJ, Townsend DM, Morris TE, Fraser JP, He L, et al. (2006) A Novel Role for Human Sulfiredoxin in the Reversal of Glutathionylation. Cancer Res 66: 6800-6806.
52. Lonergan KM, Chari R, deLeeuw RJ, Shadeo A, Chi B, et al. (2006) Identification of Novel Lung Genes in Bronchial Epithelium by Serial Analysis of Gene Expression. Am J Respir Cell Mol Biol 35: 651-661.
53. Chari R, Lonergan K, Ng R, MacAulay C, Lam W, et al. (2007) Effect of active smoking on the human bronchial epithelium transcriptome. BMC Genomics 8: 297.
54. Wu CG, Groenink M, Bosma A, Reitsma PH, van Deventer SJ, et al. (1997) Rat ferritin-H: cDNA cloning, differential expression and localization during hepatocarcinogenesis. Carcinogenesis 18: 47-52.

55. Torti SV, Kwak EL, Miller SC, Miller LL, Ringold GM, et al. (1988) The molecular cloning and characterization of murine ferritin heavy chain, a tumor necrosis factor-inducible gene. J Biol Chem 263: 12638-12644.

56. Patel JH, Loboda AP, Showe MK, Showe LC, McMahon SB (2004) Analysis of genomic targets reveals complex functions of MYC. Nat Rev Cancer 4: 562-568.

57. Andersen JS, Lam YW, Leung AKL, Ong S-E, Lyon CE, et al. (2005) Nucleolar proteome dynamics. Nature 433: 77-83.

58. Wang T, Zeng J, Lowe CB, Sellers RG, Salama SR, et al. (2007) Species-specific endogenous retroviruses shape the transcriptional network of the human tumor suppressor protein p53. Proceedings of the National Academy of Sciences 104: 18613-18618.

59. Dos Santos ML, Palanch CG, Salaorni S (2006) Transcriptome characterization of human mammary cell lines expressing different levels of ERBB2 by serial analysis of gene expression. INTERNATIONAL JOURNAL OF ONCOLOGY 28: 1441.

60. Wodarz D, Komarova NL (2005) Computational biology of cancer: lecture notes and mathematical modeling: World Scientific.

61. Nakamura T, Fidler IJ, Coombes KR (2007) Gene Expression Profile of Metastatic Human Pancreatic Cancer Cells Depends on the Organ Microenvironment. Cancer Res 67: 139-148.

62. Lee S, Medina D, Tsimelzon A, Mohsin SK, Mao S, et al. (2007) Alterations of Gene Expression in the Development of Early Hyperplastic Precursors of Breast Cancer. Am J Pathol 171: 252-262.

63. Pearson G, Robinson F, Beers Gibson T, Xu B-e, Karandikar M, et al. (2001) Mitogen-Activated Protein (MAP) Kinase Pathways: Regulation and Physiological Functions. Endocr Rev 22: 153-183.
