# Supplementary figures and images for: Cancer Reduces Transcriptome Specialization
Source: PLoS One. 2010 May 3;5(5):e10398. doi: 10.1371/journal.pone.0010398 (PMC2862708; doi:10.1371/journal.pone.0010398)

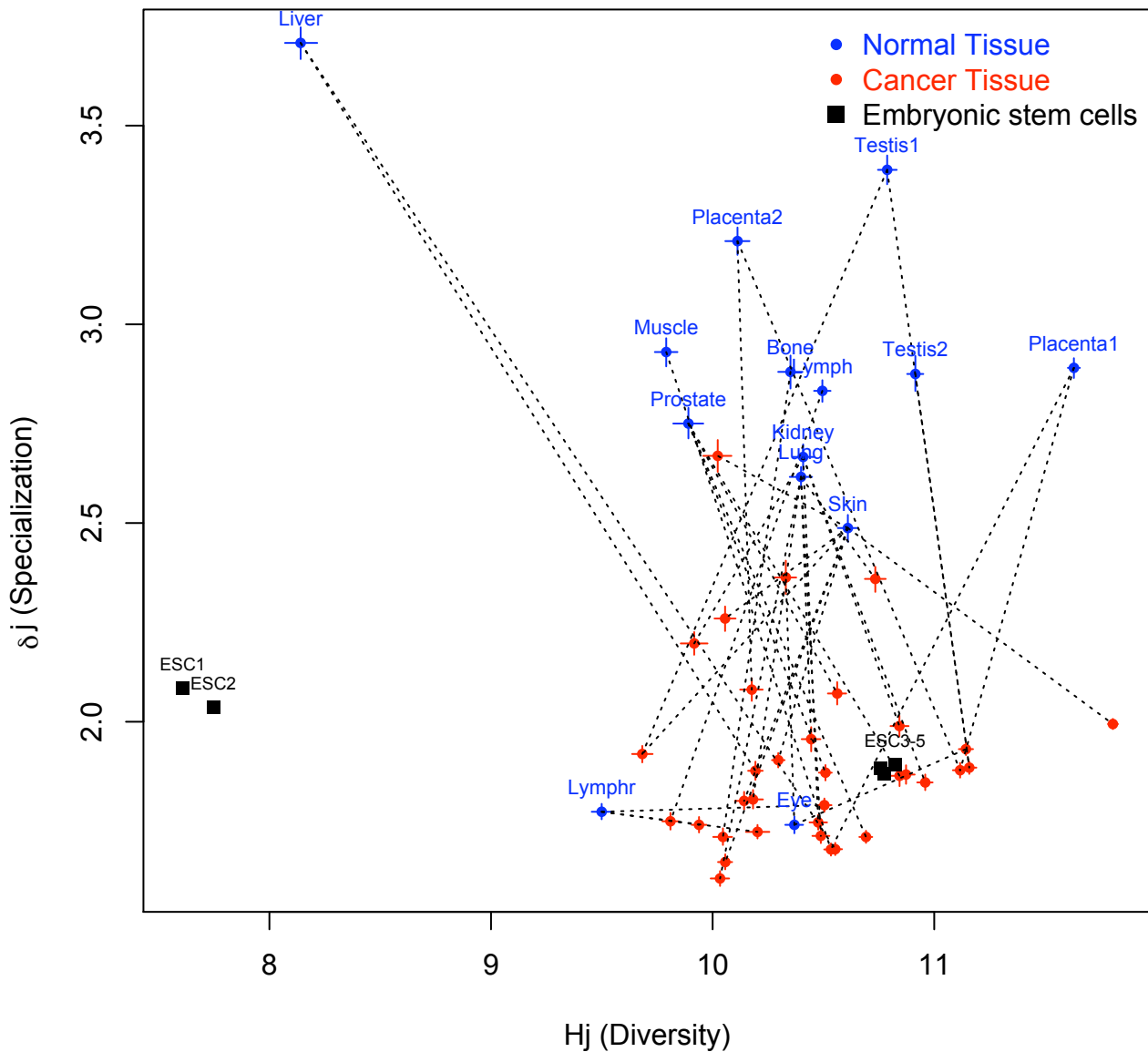

Supplement: Figure S1 — Estimated values of Hj (diversity) and δj (specialization) in each one of the libraries of dataset A, ungrouped analysis. (0.22 MB PDF) [file pone.0010398.s002.pdf]

**Bone**

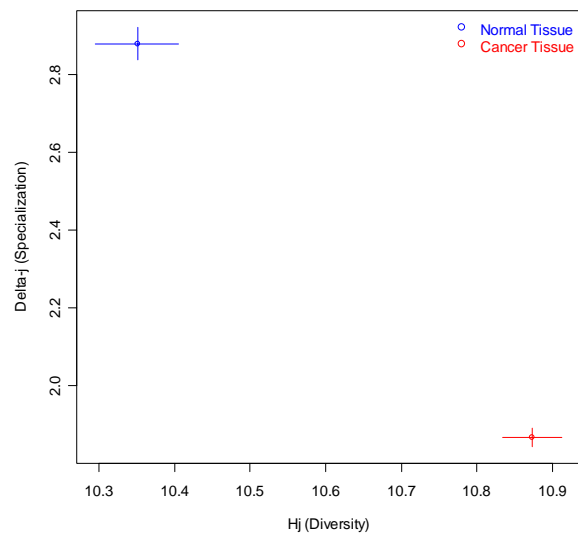

**Eye**

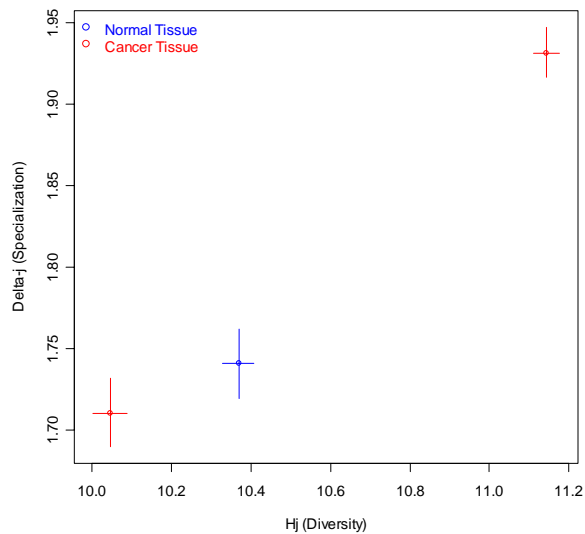

**Kidney**

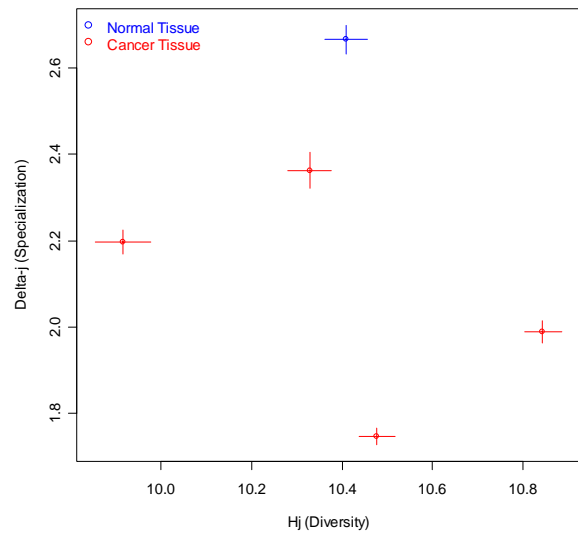

**Liver**

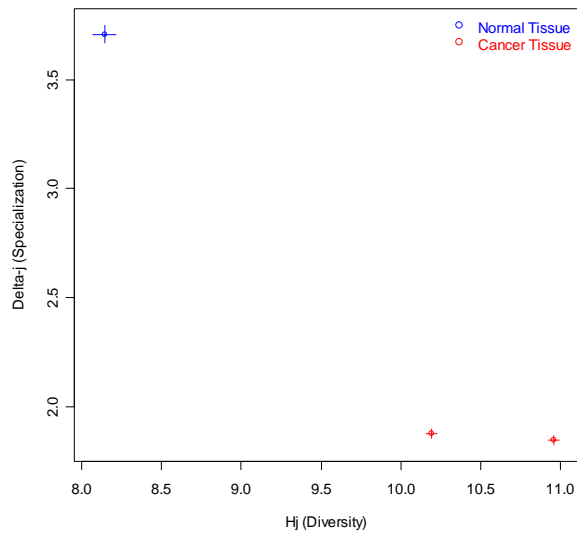

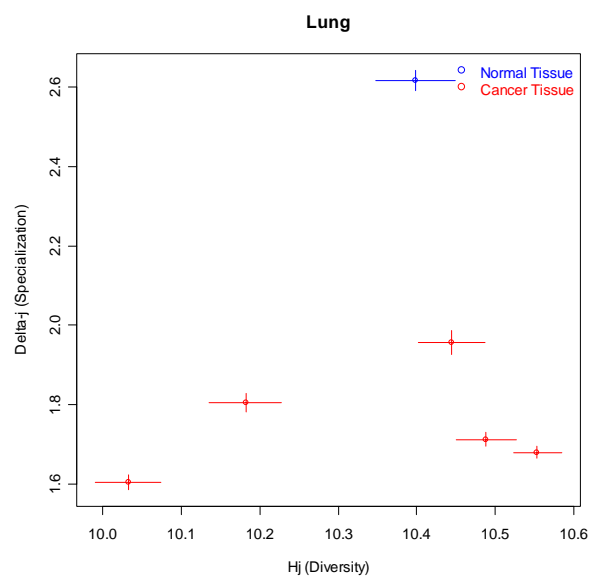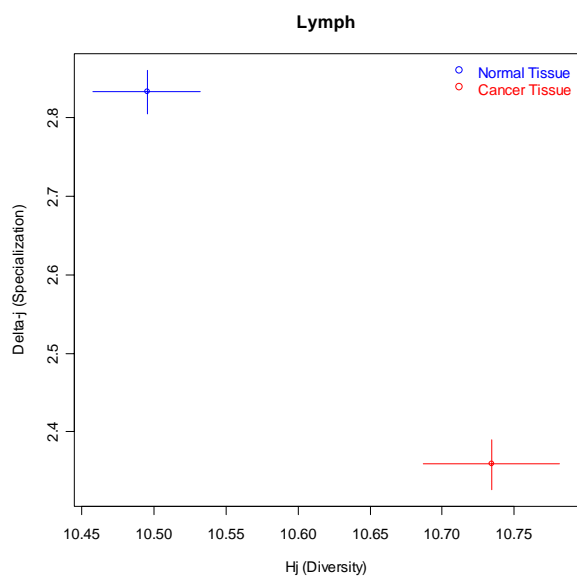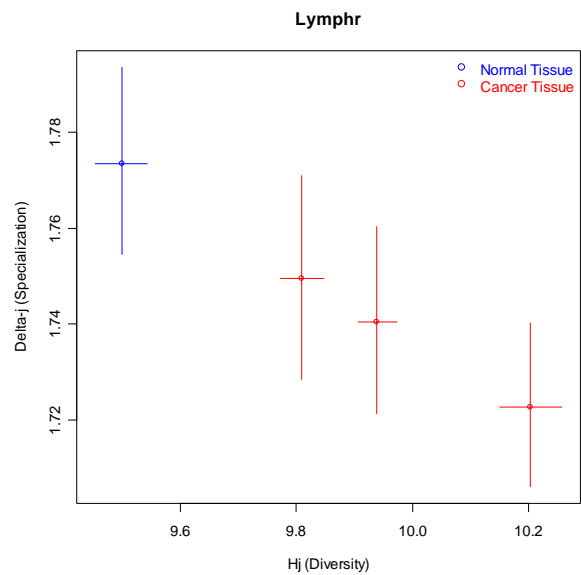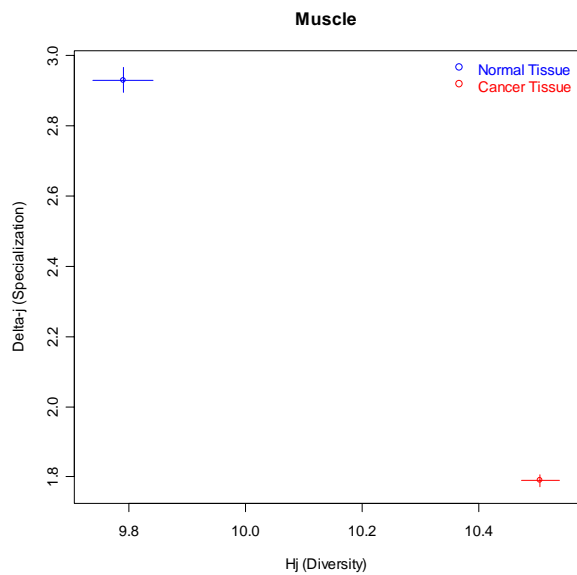

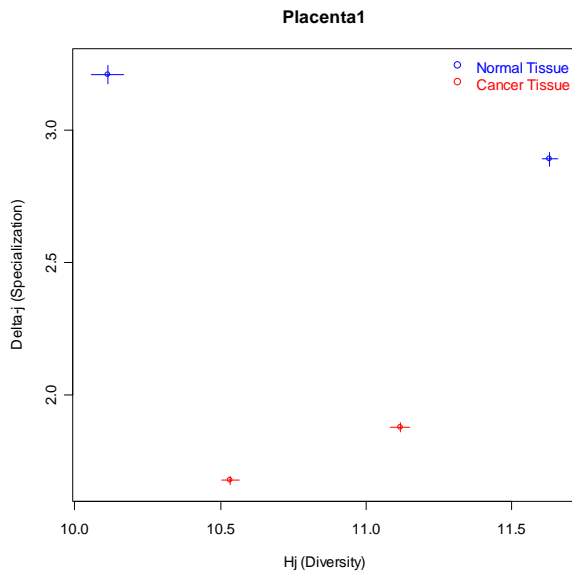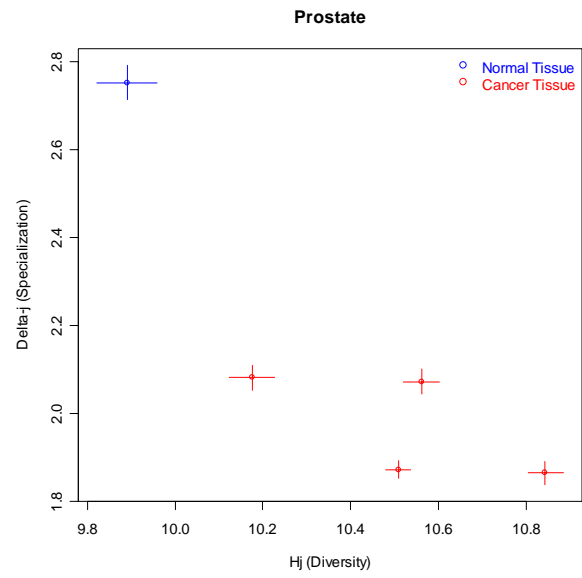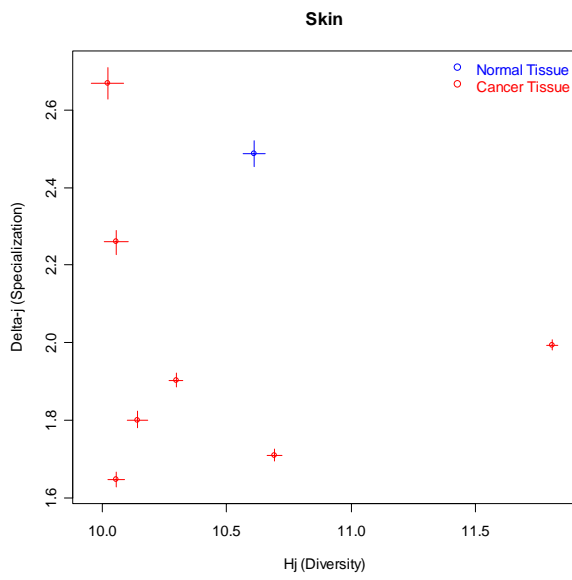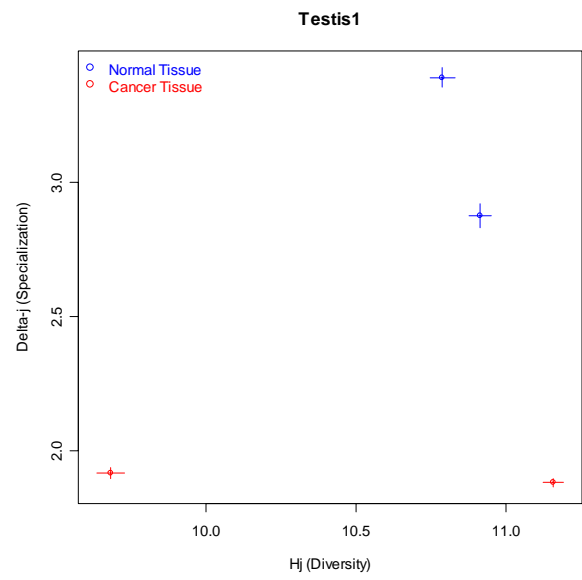

Supplement: Figure S2 — Estimated values of Hj (diversity) and δj (specialization) in each one of the libraries of dataset A, non-grouped analysis. One panel per organ. (0.03 MB PDF) [file pone.0010398.s003.pdf]
