# Supplementary material for: Cancer Reduces Transcriptome Specialization
Source: PLoS One. 2010 May 3;5(5):e10398. doi: 10.1371/journal.pone.0010398 (PMC2862708; doi:10.1371/journal.pone.0010398)
Supplement: Figure S4 — Estimated values of Hj (diversity) and δj (specialization) in each one of the libraries of dataset B (mouse data), non-grouped analysis. (0.07 MB PDF) [file pone.0010398.s005.pdf]

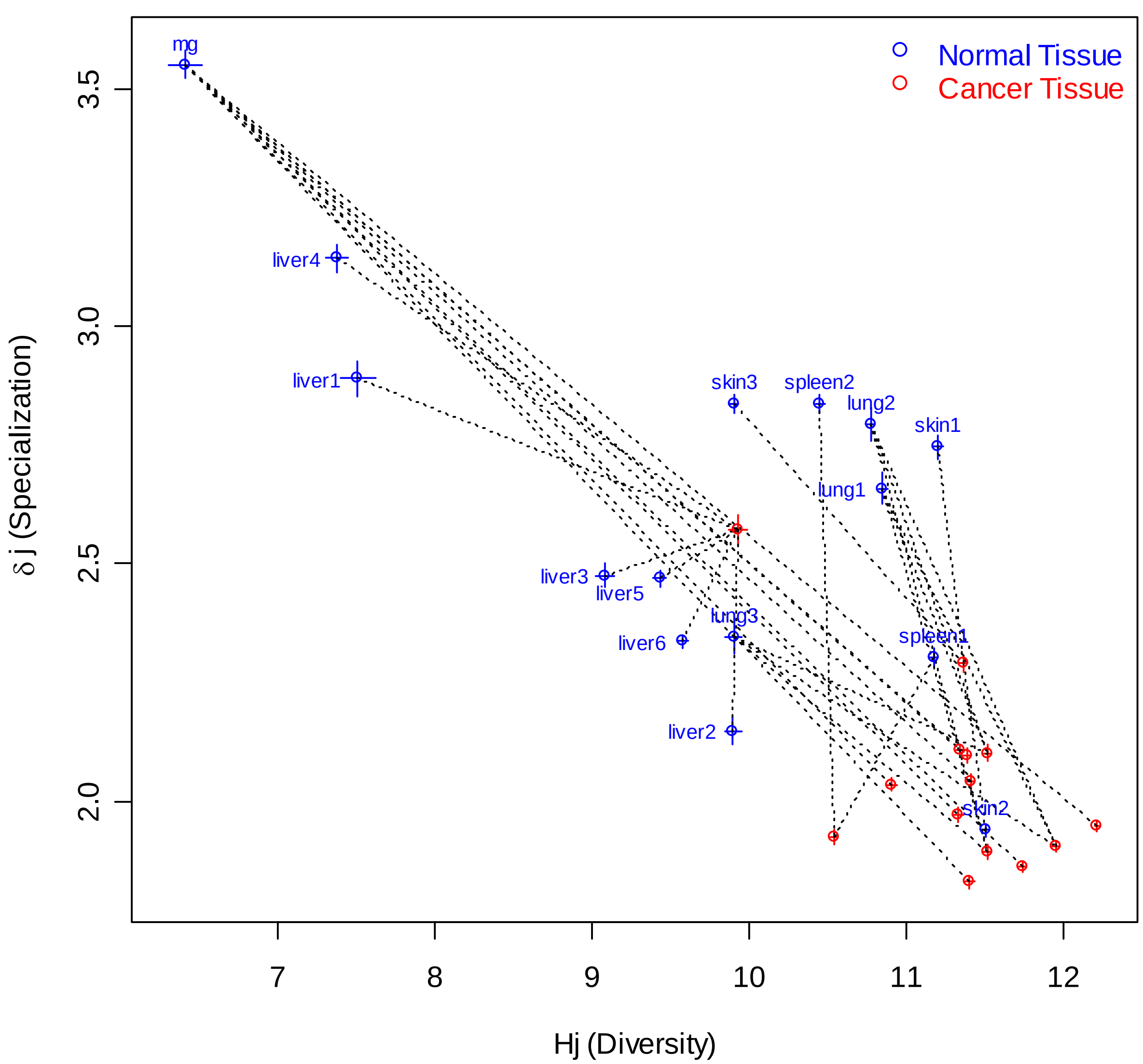

Fig. S4. Estimated values of H_j (diversity) and δ_j (specialization) in each one of the libraries of dataset ***B*** (mouse data), non-grouped analysis. Open circles are plotted in the mean of the 2000 bootstrap replicates for each parameter and the corresponding approximate 95% confidence intervals are plotted as continuous lines in each axe. Discontinuous lines join comparable libraries (same tissue). For clarity the graph for each set of comparable mouse libraries are presented in Fig. S5.
