# Supplementary material for: Cancer Reduces Transcriptome Specialization
Source: PLoS One. 2010 May 3;5(5):e10398. doi: 10.1371/journal.pone.0010398 (PMC2862708; doi:10.1371/journal.pone.0010398)
Supplement: Figure S5 — Estimated values of Hj (diversity) and δj (specialization) in each one of the libraries of dataset B (mouse data), non-grouped analysis. One panel per organ. (0.08 MB PDF) [file pone.0010398.s006.pdf]

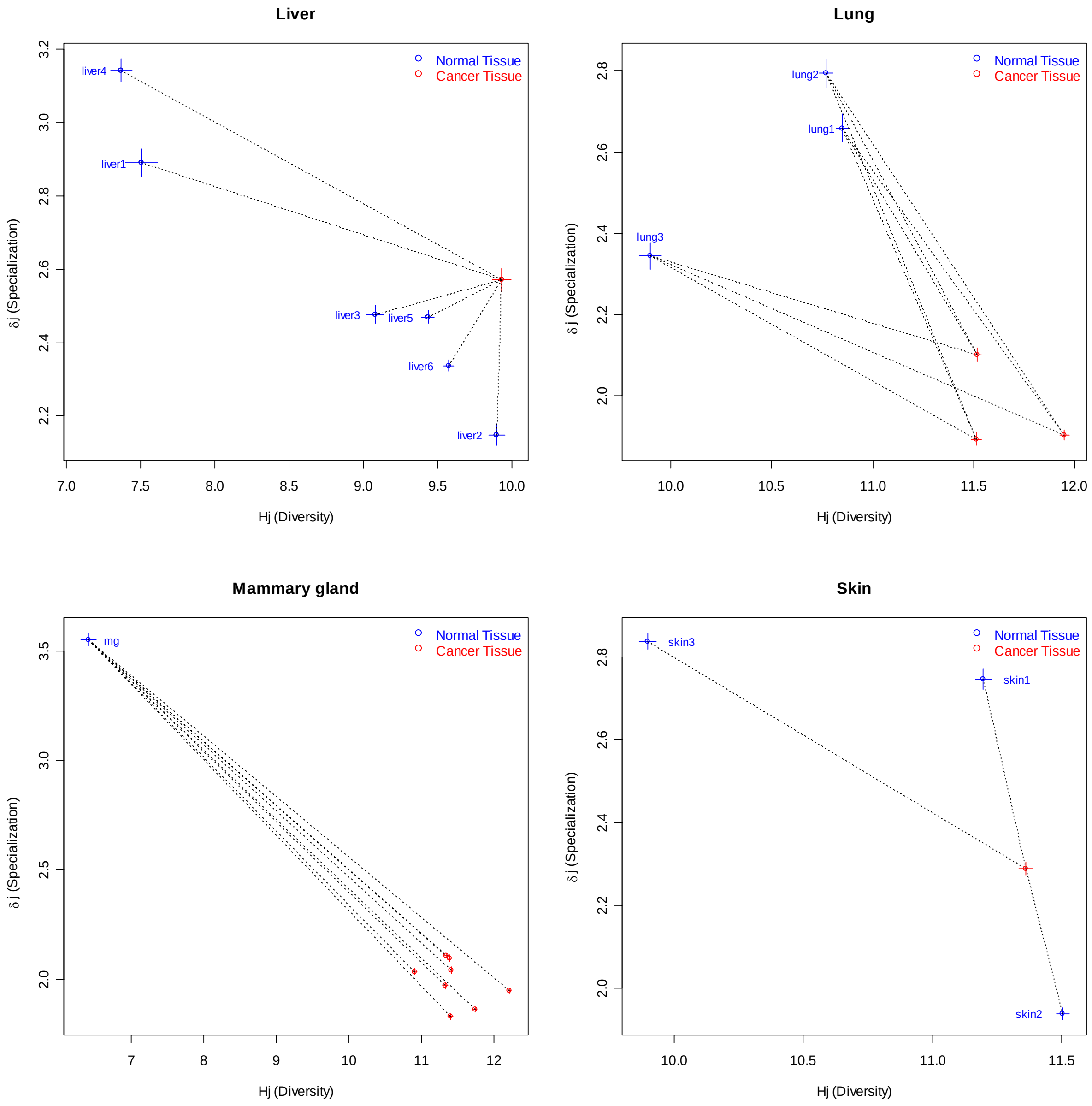

Liver
Normal Tissue
Cancer Tissue
liver4
liver1
liver3
liver5
liver6
liver2
δj (Specialization)
Hj (Diversity)
Lung
lung2
lung1
lung3
Mammary gland
mg
Skin
skin3
skin1
skin2

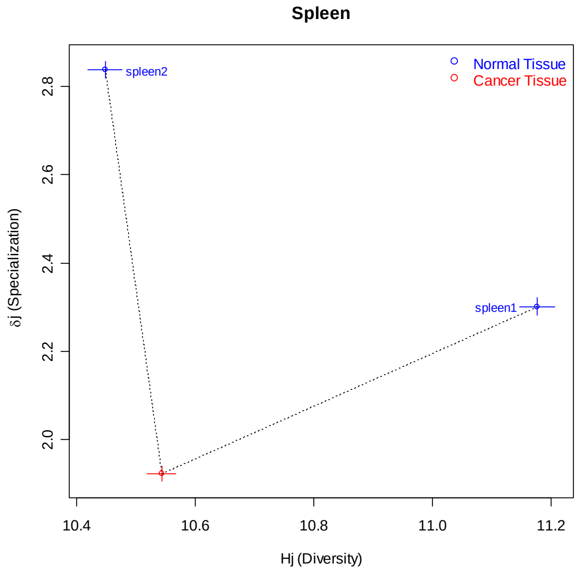

Fig. S5. Estimated values of H_j (diversity) and δ_j (specialization) in each one of the libraries of dataset ***B*** (mouse data), non-grouped analysis. Open circles are plotted in the mean of the 2000 bootstrap replicates for each parameter and the corresponding approximate 95% confidence intervals are plotted as continuous lines in each axe. Each one of the panels presents the results for one organ.
