# Supplementary material for: Cancer Reduces Transcriptome Specialization
Source: PLoS One. 2010 May 3;5(5):e10398. doi: 10.1371/journal.pone.0010398 (PMC2862708; doi:10.1371/journal.pone.0010398)
Supplement: Figure S6 — Estimated values of Hj (diversity) and δj (specialization) in normal and cancerous transcriptomes obtained by grouping all organs in dataset B (mouse data). (0.05 MB PDF) [file pone.0010398.s007.pdf]

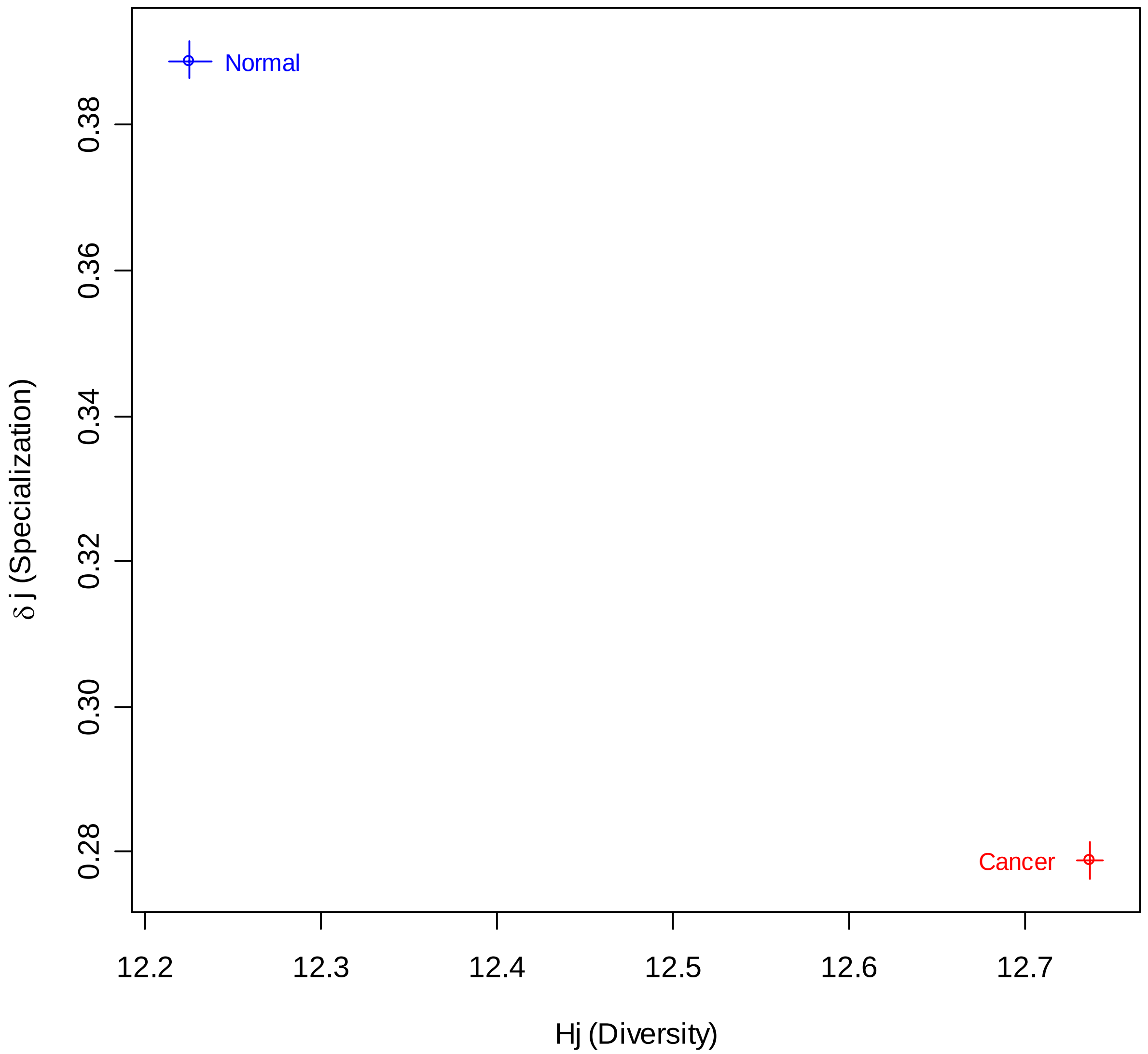

Fig. S6. Estimated values of H_j (diversity) and δ_j (specialization) in normal and cancerous transcriptomes obtained by grouping all organs in dataset ***B*** (mouse data). Open circles are plotted in the mean of the 2000 bootstrap replicates for each parameter and the corresponding approximate 95% confidence intervals are plotted as continuous lines in each axe.
