# Supplementary material for: Cancer Reduces Transcriptome Specialization
Source: PLoS One. 2010 May 3;5(5):e10398. doi: 10.1371/journal.pone.0010398 (PMC2862708; doi:10.1371/journal.pone.0010398)
Supplement: Figure S8 — Estimated values of Hj (diversity) and δj (specialization) in chromosomes 21, 18, 13, 22, 20 and X (dataset C). (0.05 MB PDF) [file pone.0010398.s009.pdf]

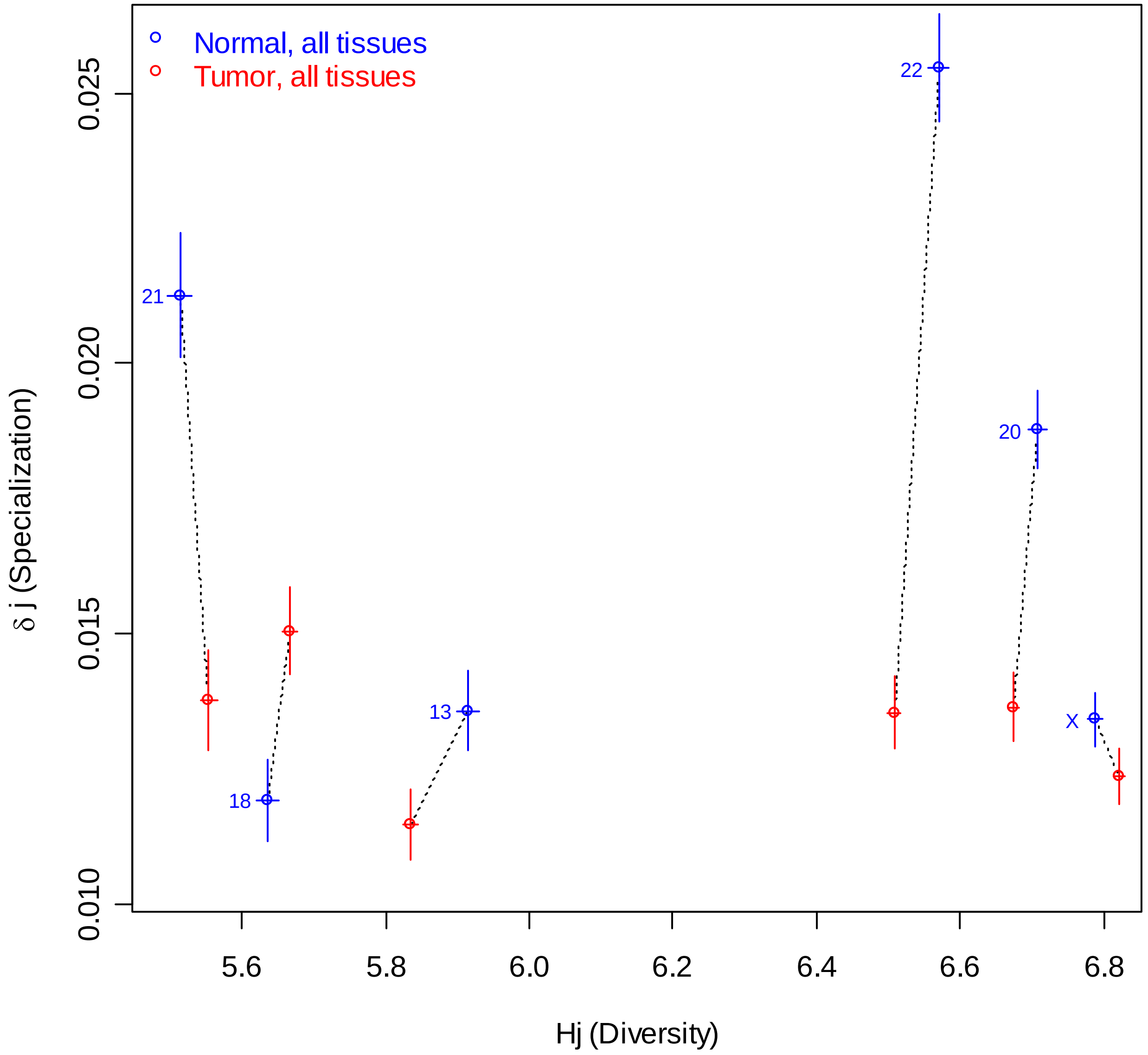

Fig. S8. Estimated values of H_j (diversity) and δ_j (specialization) in chromosomes 21, 18, 13, 22, 20 and X (dataset ***C***). Open circles are plotted in the mean of the 2000 bootstrap replicates for each parameter and the corresponding approximate 95% confidence intervals are plotted as continuous lines in each axe. This figure amplifies the brown box of Fig. 2.
