# Supplementary material for: Cancer Reduces Transcriptome Specialization
Source: PLoS One. 2010 May 3;5(5):e10398. doi: 10.1371/journal.pone.0010398 (PMC2862708; doi:10.1371/journal.pone.0010398)
Supplement: Figure S9 — Estimated values of Hj (diversity) and δj (specialization) in chromosomes 14, 8, 15, 9, 10, 16, 4, 6, 7, 5, 11, 3, 12, 17, 19 and 2. Dataset C. (0.06 MB PDF) [file pone.0010398.s010.pdf]

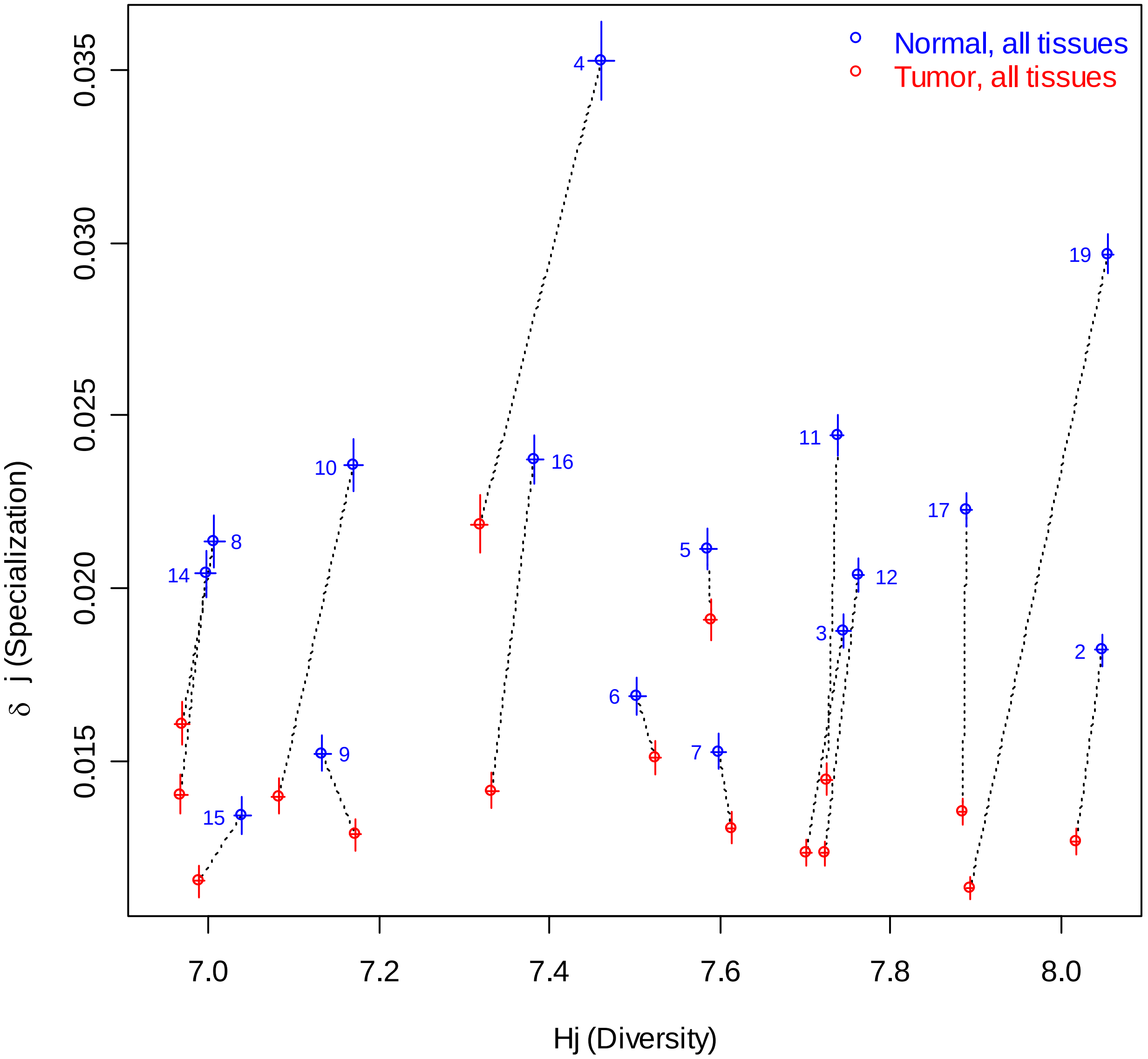

Fig. S9. Estimated values of H_j (diversity) and δ_j (specialization) in chromosomes 14, 8, 15, 9, 10, 16, 4, 6, 7, 5, 11, 3, 12, 17, 19 and 2. Dataset ***C***. Open circles are plotted in the mean of the 2000 bootstrap replicates for each parameter and the corresponding approximate 95% confidence intervals are plotted as continuous lines in each axe. This figure amplifies the purple box of Fig. 2.
