# Supplementary material for: Cancer Reduces Transcriptome Specialization
Source: PLoS One. 2010 May 3;5(5):e10398. doi: 10.1371/journal.pone.0010398 (PMC2862708; doi:10.1371/journal.pone.0010398)
Supplement: Figure S11 — Example of scatter plot for the differences in expression in Dataset A (Human dataset). (0.36 MB PDF) [file pone.0010398.s012.pdf]

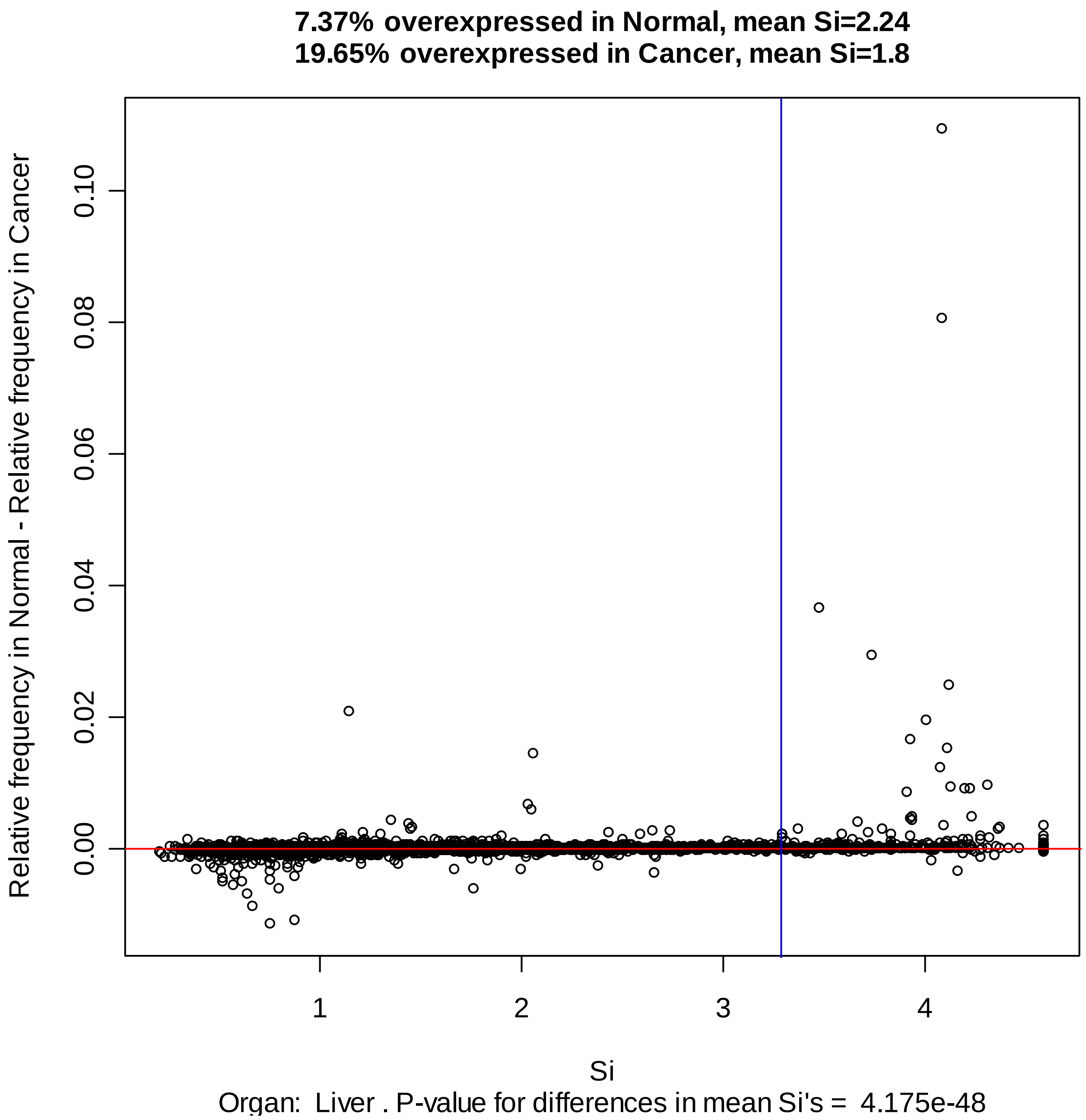

Figure S11. Example of scatter plot for the differences in expression in Dataset ***A*** (Human dataset). Relative frequency in Normal – Relative frequency in Cancer = p_{ij} - p_{ik} *versus* values of gene specificities (S_i). The percentages of genes over-expressed in each condition and the corresponding mean values of these gene specificities are presented in the title. The organ and p-value for the tests of the differences between the gene specificities in each case are presented in the subtitle. Points above the red line are over-expressed in normal tissues; points below the red line are over-expressed in cancer. The blue line points to the mean of gene specificities.
