# Supplementary material for: Cancer Reduces Transcriptome Specialization
Source: PLoS One. 2010 May 3;5(5):e10398. doi: 10.1371/journal.pone.0010398 (PMC2862708; doi:10.1371/journal.pone.0010398)
Supplement: Figure S12 — Example of scatter plot for the differences in expression in Dataset B (Mouse dataset). (0.38 MB PDF) [file pone.0010398.s013.pdf]

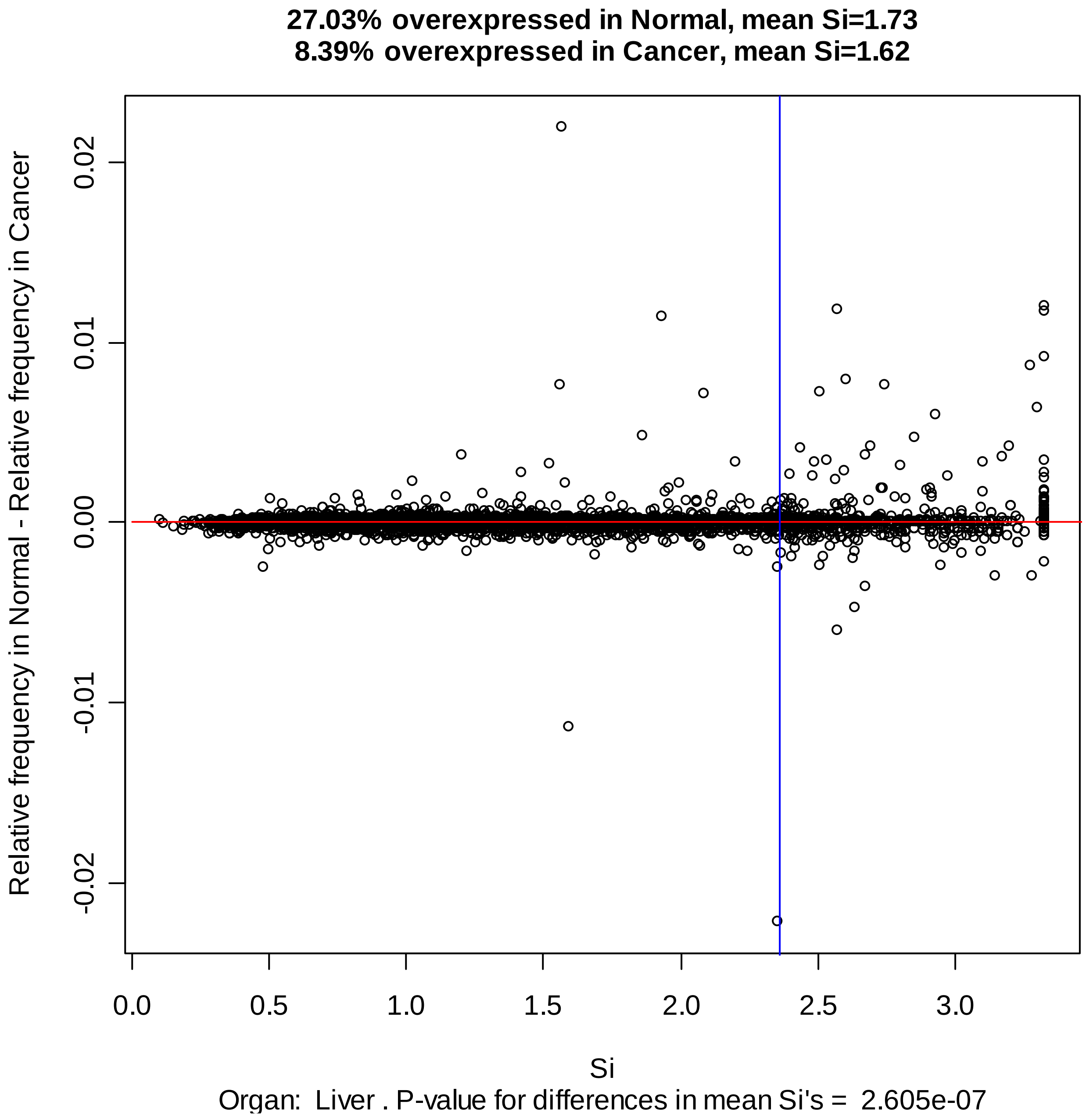


Fig. S12. Example of scatter plot for the differences in expression in Dataset ***B*** (Mouse dataset). Relative frequency in Normal – Relative frequency in Cancer = $p_{ij}$ - $p_{ik}$ *versus* values of gene specificities ($S_i$). The percentages of genes over-expressed in each condition and the corresponding mean values of these gene specificities are presented in the titles. The organ and p-value for the tests of the differences between the gene specificities in each case are presented in the subtitle. Points above the red line are over-expressed in normal tissue; points below the red line are over-expressed in cancer. The blue line points to the mean of gene specificities.
