# Supplementary material for: Cancer Reduces Transcriptome Specialization
Source: PLoS One. 2010 May 3;5(5):e10398. doi: 10.1371/journal.pone.0010398 (PMC2862708; doi:10.1371/journal.pone.0010398)
Supplement: Figure S13 — Scatter plot of Hj (Diversity) and δj (Specialization) in transcriptomes of normal (blue) and precancerous (red) tissues in dataset D. (0.10 MB PDF) [file pone.0010398.s014.pdf]

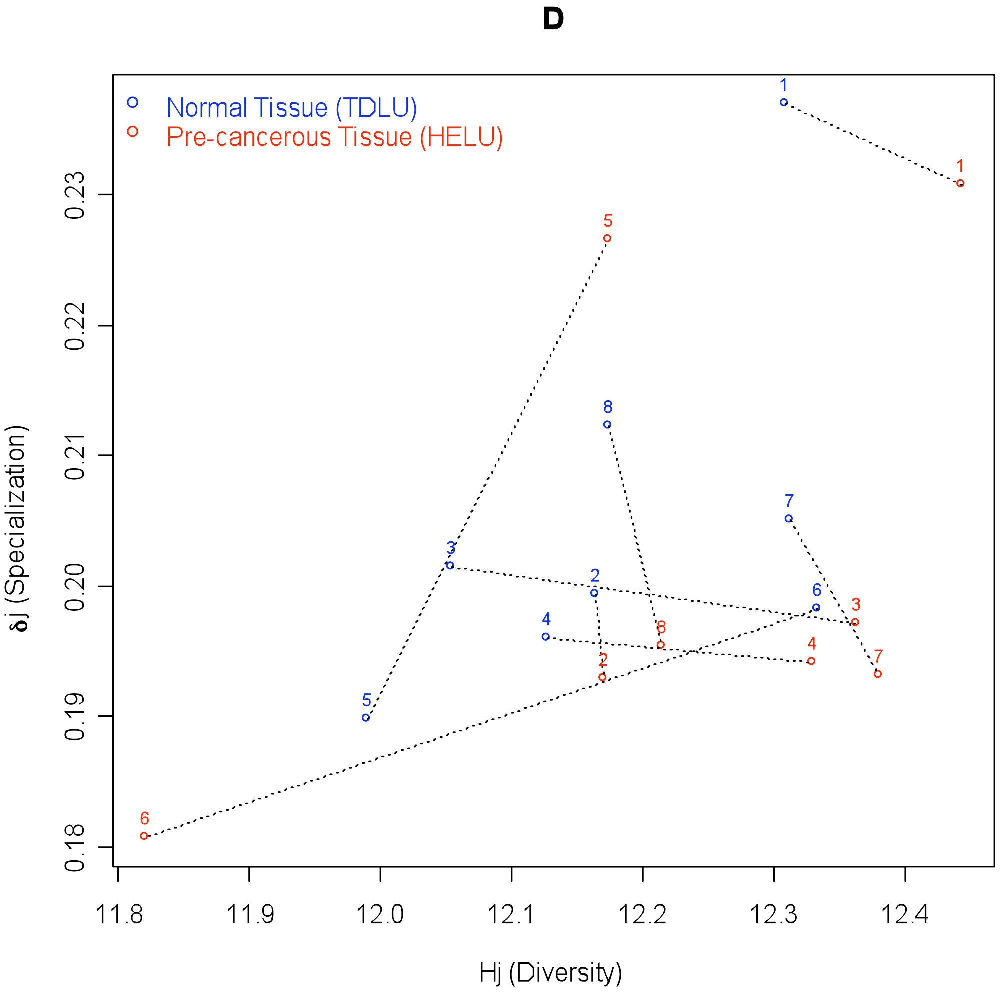

Figure S13. Scatter plot of H_j (Diversity) and δ_j (Specialization) in transcriptomes of normal (blue) and precancerous (red) tissues in dataset ***D***, NCBI GEO accession GDS2739; Human microarray paired samples of normal terminal duct lobular units (TDLUs; normal tissue) and hyperplastic enlarged lobular units (HELUs; precancerous tissues) from eight patients.
