# Supplementary material for: Cancer Reduces Transcriptome Specialization
Source: PLoS One. 2010 May 3;5(5):e10398. doi: 10.1371/journal.pone.0010398 (PMC2862708; doi:10.1371/journal.pone.0010398)
Supplement: Table S1 — Human libraries from the "Cancer Genome Anatomy Project" selected for analysis (Dataset A). (0.07 MB PDF) [file pone.0010398.s015.pdf]

Name	Lib. Id.	Tags Sum	Tissue Description	Tissue	Histology
Bone	lib10410	5230	Bone, subchondral	Bone	Normal
BoneC	lib8568	9539	Bone, osteosarcoma	Bone	neoplasia
Eye	lib7269	10679	Eye, normal, pigmented retinal epithelium	Eye	Normal
EyeC1	lib5374	15241	Eye, retinoblastoma	Eye	neoplasia
EyeC2	lib5606	26788	Eye, retinoblastoma	Eye	neoplasia
Kidney	lib6833	7559	Kidney	Kidney	normal
KidneyC1	lib991	5010	Kidney, 5 pooled tumors, including broad spectrum of kidney tumor types	Kidney	neoplasia
KidneyC2	lib4068	13901	Kidney, renal cell adenocarcinoma	Kidney	neoplasia
KidneyC3	lib5950	6535	Kidney, hypernephroma	Kidney	neoplasia
KidneyC4	lib8646	8414	Kidney, hypernephroma	kidney	neoplasia
Liver	lib6989	9399	Liver	Liver	normal
LiverC1	lib8584	13397	Liver, adenocarcinoma	Liver	neoplasia
LiverC2	lib9631	13728	Liver, hepatocellular carcinoma	Liver	neoplasia
Lung	lib6834	12534	Lung	Lung	normal
LungC1	lib2586	22674	Lung, small cell carcinoma	Lung	neoplasia
LungC2	lib5607	17379	Lung, large cell carcinoma	Lung	neoplasia
LungC3	lib5608	5763	Lung, carcinoma, large cell undifferentiated.	Lung	neoplasia
LungC4	lib7012	13824	Lung, large cell carcinoma	Lung	neoplasia
LungC5	lib10453	8805	Lung, epidermoid carcinoma	Lung	neoplasia
Lymph	lib3718	9861	Lymph, normal germinal center B cells	lymph node	normal
LymphC	lib1451	9193	Lymphoma, follicular mixed small and large cell	lymph node	neoplasia
Lymphr	lib6987	16393	Primary B-Cells from Tonsils	lymphoreticular	normal
LymphrC1	lib4069	12720	Lymph, Burkitt lymphoma	lymphoreticular	neoplasia
LymphrC2	lib7318	15591	Lymph, lymphoma	lymphoreticular	neoplasia
LymphrC3	lib9630	15586	Lymph, lymphoma	lymphoreticular	neoplasia
Muscle	lib6761	9316	Skeletal Muscle	muscle	normal
MuscleC	lib3714	23777	Muscle, rhabdomyosarcoma	muscle	neoplasia
Placenta1	lib368	13966	Placenta, two pooled, one 8 weeks, one 9 weeks post conception.	placenta	normal
Placenta2	lib6835	9289	Placenta	placenta	normal
PlacentaC1	lib4070	21622	Placenta, choriocarcinoma	placenta	neoplasia
PlacentaC2	lib2587	17623	Placenta, choriocarcinoma	placenta	neoplasia
Prostate	lib6763	6729	Prostate	prostate	normal
ProstateC1	lib6831	6780	Prostate, adenocarcinoma.	prostate	neoplasia
ProstateC2	lib8585	8320	Prostate, adenocarcinoma.	prostate	neoplasia
ProstateC3	lib8834	15603	Prostate, carcinoma	prostate	neoplasia
ProstateC4	lib14129	8053	Prostate	prostate	neoplasia
Skin	lib8848	8042	Skin, normal	Skin	normal
SkinC1	lib5346	21672	Skin, melanotic melanoma.	Skin	neoplasia
SkinC2	lib6757	7072	Skin, melanotic melanoma, high MDR.	Skin	neoplasia
SkinC3	lib5610	37803	Skin, melanotic melanoma.	Skin	neoplasia
SkinC4	lib7270	10613	Skin, melanotic melanoma, high MDR.	Skin	neoplasia
SkinC5	lib8775	14810	Skin, melanoma, amelanotic	Skin	neoplasia
SkinC6	lib9901	18725	Skin, melanoma, melanotic	Skin	neoplasia
SkinC7	lib8849	6708	Skin, squamous cell carcinoma	Skin	neoplasia
Testis1	lib6762	7623	Testis	Testis	normal
Testis2	lib13710	5003	Testis	Testis	normal
TestisC1	lib6832	11635	Testis, embryonal carcinoma	Testis	neoplasia
TestisC2	lib8657	24169	Testis, embryonal carcinoma	Testis	neoplasia
ESC1	lib13039	9790	Embryonic stem cells, WA01, passage 38	Embryonic	normal
ESC2	lib13040	9984	Embryonic trophoblasts, made from WA01 stem cells	Embryonic	normal
ESC3	lib16754	8819	Embryonic stem cells, isolated from the Inner Cell Mass of Blastocyst stage embryos and differentiated to an early endodermal cell type	Embryonic	normal

ESC4	lib16981	12858	mbryonic stem cells, isolated from the Inner Cell Mass of Blastocyst stage embryos and differentiated to an early neural progentior cell type	Embryonic	normal
ESC5	lib16958	9050	Embryonic stem cells, isolated from the Inner Cell Mass of Blastocyst stage embryos	Embryonic	normal

Name – Name given to the library in the text and figures. Lib. Id. – Identification number of the library.
Tags Sum – Total number of tags in the library. Histology – Reported histology of the tissue.
