# Supplementary material for: Cancer Reduces Transcriptome Specialization
Source: PLoS One. 2010 May 3;5(5):e10398. doi: 10.1371/journal.pone.0010398 (PMC2862708; doi:10.1371/journal.pone.0010398)
Supplement: Table S2 — Mouse libraries from the "Cancer Genome Anatomy Project" selected for analysis (Dataset B). (0.05 MB PDF) [file pone.0010398.s016.pdf]

**Name**	**Lib._id.**	**Tags sum**	**Tissue**	**Histology**
liver1	1883	5010	Liver	normal
liver2	4141	5292	Liver	normal
liver3	12883	7087	Liver	normal
liver4	12109	7251	Liver	normal
liver5	1299	20034	Liver	normal
liver6	7065	26656	Liver	normal
liverC	7225	5322	Liver	neoplasia
lung1	5469	5100	Lung	normal
lung2	5497	5101	Lung	normal
lung3	7216	5688	Lung	normal
lungC1	2728	14976	Lung	neoplasia
lungC2	9962	16368	Lung	neoplasia
lungC3	2727	27882	Lung	neoplasia
mg	5493	7248	Mammary gland	normal
mgC1	9963	15024	Mammary gland	neoplasia
mgC2	9964	19668	Mammary gland	neoplasia
mgC3	2745	20831	Mammary gland	neoplasia
mgC4	2726	22321	Mammary gland	neoplasia
mgC5	2744	23406	Mammary gland	neoplasia
mgC6	2568	29910	Mammary gland	neoplasia
mgC7	2569	40311	Mammary gland	neoplasia
mgC8	2754	44194	Mammary gland	neoplasia
skin1	498	14795	Skin	normal
skin2	9852	19192	Skin	normal
skin3	12278	34026	Skin	normal
skinC	9853	24941	Skin	neoplasia
spleen1	9847	13744	Spleen	normal
spleen2	12279	25341	Spleen	normal
spleenC	12254	16845	Spleen	neoplasia
HSC	17889	21341	Hematopoietic stem cells	normal

Name – Name given to the library in the text and figures.
Lib. Id. – Identification number of the library.
Tags Sum – Total number of tags in the library.
Histology – Reported histology of the tissue.
