# Supplementary material for: Cancer Reduces Transcriptome Specialization
Source: PLoS One. 2010 May 3;5(5):e10398. doi: 10.1371/journal.pone.0010398 (PMC2862708; doi:10.1371/journal.pone.0010398)
Supplement: Table S3 — Number of tags and loci per chromosome in dataset C (Human normal and tumor tissues from the "Human Transcriptome Map"). (0.02 MB PDF) [file pone.0010398.s017.pdf]

Chromosome	Number of tags		Number of Loci
	Normal	Tumor	
1	533,083	1,200,614	6,347
2	400,670	900,062	4,631
3	351,882	797,785	3,870
4	128,700	288,371	2,715
5	234,584	510,554	3,135
6	266,215	591,756	3,321
7	273,493	636,203	3,088
8	177,118	414,116	2,451
9	247,234	530,245	2,514
10	207,326	464,192	2,666
11	327,027	719,230	3,380
12	387,683	854,985	3,385
13	105,466	243,002	1,341
14	184,972	411,688	2,050
15	216,591	503,892	2,078
16	235,887	528,369	2,444
17	413,801	912,081	3,411
18	92,302	203,690	1,073
19	394,969	895,025	3,227
20	147,104	342,046	1,662
21	70,981	157,949	753
22	126,751	278,237	1,316
X	208,267	444,064	1,952
Y	15,729	33,084	106
All chromosomes	5,747,834	12,861,239	62,916
