# Supplementary material for: Cancer Reduces Transcriptome Specialization
Source: PLoS One. 2010 May 3;5(5):e10398. doi: 10.1371/journal.pone.0010398 (PMC2862708; doi:10.1371/journal.pone.0010398)
Supplement: Table S4 — Approximate 99% Confidence Intervals for the difference between specializations in all pairs of comparable tissues (normal versus cancer) in dataset A (grouped analysis). (0.02 MB PDF) [file pone.0010398.s018.pdf]

**Comparison**	**Difference**	**S(Difference)**	**Lower limit**	**Upper limit**	**Shapiro P**
Eye – EyeC	-0.0658	0.0110	-0.0873	-0.0446	0.8456
Lymphr – LymphrC	0.0695	0.0095	0.0505	0.0876	0.5590
Lymph – LymphC	0.4554	0.0178	0.4214	0.4920	0.1441
Skin – SkinC	0.4614	0.0153	0.4311	0.4903	0.1737
Kidney – KidneyC	0.5391	0.0160	0.5098	0.5704	0.0840
Lung – LungC	0.6278	0.0115	0.6053	0.6508	0.4258
Prostate – ProstateC	0.6300	0.0172	0.5958	0.6640	0.1596
Muscle – MuscleC	0.8404	0.0172	0.8067	0.8740	0.9142
Bone – CONEC	0.8925	0.0208	0.8519	0.9332	0.7187
Placenta – PlacentaC	0.9725	0.0109	0.9518	0.9939	0.5251
Testis – TestisC	1.0744	0.0138	1.0473	1.1020	0.6552
Liver – LiverC	1.4559	0.0176	1.4213	1.4894	0.2792

S(Difference) – Standard deviation of the difference; Lower and Upper limits are approximate 99% limits for the true difference obtained by the Bootstrap Percentile Interval method. Shapiro P – Probability of the Shapiro-Wilks test for normality of the differences.
