# Supplementary material for: Cancer Reduces Transcriptome Specialization
Source: PLoS One. 2010 May 3;5(5):e10398. doi: 10.1371/journal.pone.0010398 (PMC2862708; doi:10.1371/journal.pone.0010398)
Supplement: Table S5 — Approximate 99% Confidence Intervals for the difference between specializations in all pairs of comparable libraries (non-grouped analysis, dataset A). (0.03 MB PDF) [file pone.0010398.s019.pdf]

Comparison	Difference	S(Difference)	Lower limit	Upper limit	Shapiro P
Bone – BoneC	1.0132	0.0243	0.9643	1.0614	0.1051
Eye - EyeC1	0.0305	0.0149	0.0009	0.0591	0.2758
Eye - EyeC2	-0.1905	0.0132	-0.2162	-0.1650	0.9832
Kidney - KidneyC1	0.3033	0.0273	0.2492	0.3566	0.7864
Kidney - KidneyC2	0.9196	0.0189	0.8809	0.9562	0.4410
Kidney - KidneyC3	0.4692	0.0220	0.4256	0.5127	0.4173
Kidney - KidneyC4	0.6772	0.0213	0.6358	0.7175	0.7286
Liver - LiverC1	1.8608	0.0220	1.8174	1.9038	0.8945
Liver - LiverC2	1.8315	0.0229	1.7840	1.8759	0.0434
Lung - LungC1	0.9368	0.0151	0.9069	0.9661	0.8153
Lung - LungC2	0.9033	0.0157	0.8733	0.9339	0.7785
Lung - LungC3	0.6599	0.0204	0.6205	0.7002	0.3846
Lung - LungC4	1.0104	0.0158	0.9800	1.0417	0.2541
Lung - LungC5	0.8115	0.0175	0.7783	0.8464	0.7101
Lymph – LymphC	0.4732	0.0212	0.4309	0.5147	0.8984
Lymphr - LymphrC1	0.0240	0.0143	-0.0038	0.0509	0.6990
Lymphr - LymphrC2	0.0507	0.0129	0.0247	0.0766	0.3532
Lymphr - LymphrC3	0.0329	0.0138	0.0066	0.0598	0.8785
Muscle – MuscleC	1.1402	0.0202	1.1007	1.1791	0.8540
Placenta1 - PlacentaC1	1.2119	0.0149	1.1818	1.2404	0.2454
Placenta1 - PlacentaC2	1.0125	0.0158	0.9825	1.0435	0.4092
Placenta2 - PlacentaC1	1.5308	0.0196	1.4910	1.5683	0.6835
Placenta2 - PlacentaC2	1.3314	0.0202	1.2918	1.3708	0.4525
Prostate - ProstateC1	0.6696	0.0245	0.6234	0.7182	0.0793
Prostate - ProstateC2	0.8865	0.0240	0.8389	0.9339	0.4078
Prostate - ProstateC3	0.8785	0.0220	0.8349	0.9201	0.3656
Prostate - ProstateC4	0.6793	0.0246	0.6320	0.7283	0.5930
Skin - SkinC1	0.7773	0.0187	0.7404	0.8143	0.1106
Skin - SkinC2	0.2277	0.0235	0.1831	0.2730	0.7852
Skin - SkinC3	0.4932	0.0185	0.4561	0.5290	0.5283
Skin - SkinC4	0.6866	0.0203	0.6485	0.7271	0.2480
Skin - SkinC5	0.8405	0.0198	0.7998	0.8799	0.3459
Skin - SkinC6	0.5840	0.0195	0.5466	0.6224	0.4616
Skin - SkinC7	-0.1816	0.0262	-0.2316	-0.1267	0.1177
Testis1 - TestisC1	1.4699	0.0212	1.4281	1.5115	0.1114
Testis1 - TestisC2	1.5042	0.0203	1.4648	1.5438	0.5059
Testis2 - TestisC1	0.9563	0.0241	0.9074	1.0043	0.1606
Testis2 - TestisC2	0.9906	0.0232	0.9455	1.0383	0.6804

S(Difference) – Standard deviation of the difference; Lower and Upper limits are approximate 99% limits for the true difference obtained by the Bootstrap Percentile Interval method.
ShapiroP – Probability of the Shapiro-Wilks test of normality for the differences.
