# Supplementary material for: Cancer Reduces Transcriptome Specialization
Source: PLoS One. 2010 May 3;5(5):e10398. doi: 10.1371/journal.pone.0010398 (PMC2862708; doi:10.1371/journal.pone.0010398)
Supplement: Table S6 — Approximate 99% Confidence Intervals for the difference between specializations in all pairs of comparable tissues (normal versus cancer) in the B dataset (mouse data); grouped analysis. (0.01 MB PDF) [file pone.0010398.s020.pdf]

**Comparison**	**Mean Difference**	**S(Difference)**	**LL**	**UL**	**Shapiro P**
liver – liverC	0.0542	0.0131	0.0204	0.0880	0.6213
lung - lungC	0.3109	0.0080	0.2903	0.3316	0.0668
mg – mgC	1.0826	0.0111	1.0539	1.1113	0.8195
skin - skinC	0.1289	0.0078	0.1087	0.1491	0.3198
spleen - spleenC	0.3452	0.0089	0.3223	0.3681	0.0214
S(Difference) – Standard deviation of the difference; Lower and Upper limits (LL and UL) are approximate 99% limits for the true difference obtained by the Bootstrap Percentile Interval method. Shapiro P – Probability of the Shapiro-Wilks test for normality for the differences.					
