# Supplementary material for: Cancer Reduces Transcriptome Specialization
Source: PLoS One. 2010 May 3;5(5):e10398. doi: 10.1371/journal.pone.0010398 (PMC2862708; doi:10.1371/journal.pone.0010398)
Supplement: Table S7 — Approximate 99% Confidence Intervals for the difference between specializations in all pairs of comparable libraries in dataset B (mouse data); non-grouped analysis. (0.03 MB PDF) [file pone.0010398.s021.pdf]

| **Comparison** | **Mean Difference** | **S(Difference)** | **LL** | **UL** | **Shapiro P** |
|---|---|---|---|---|---|
| Liver1 - liverC | 0.3196 | 0.0252 | 0.2558 | 0.3852 | 0.7859 |
| Liver2 - liverC | -0.4225 | 0.0210 | -0.4735 | -0.3682 | 0.1901 |
| Liver3 - liverC | -0.0943 | 0.0196 | -0.1435 | -0.0467 | 0.6766 |
| Liver4 - liverC | 0.5727 | 0.0224 | 0.5145 | 0.6305 | 0.4935 |
| Liver5 - liverC | -0.1006 | 0.0175 | -0.1443 | -0.0573 | 0.6630 |
| Liver6 - liverC | -0.2338 | 0.0170 | -0.2761 | -0.1900 | 0.9974 |
| Lung1 - lungC1 | 0.7651 | 0.0191 | 0.7197 | 0.8143 | 0.2918 |
| Lung1 - lungC2 | 0.5572 | 0.0191 | 0.5124 | 0.6081 | 0.1085 |
| Lung1 - lungC3 | 0.7555 | 0.0182 | 0.7122 | 0.8009 | 0.6281 |
| Lung2 - lungC1 | 0.9003 | 0.0192 | 0.8510 | 0.9497 | 0.7893 |
| Lung2 - lungC2 | 0.6924 | 0.0198 | 0.6408 | 0.7432 | 0.7090 |
| Lung2 - lungC3 | 0.8908 | 0.0187 | 0.8451 | 0.9387 | 0.3813 |
| Lung3 - lungC1 | 0.4518 | 0.0185 | 0.4049 | 0.4996 | 0.2176 |
| Lung3 - lungC2 | 0.2439 | 0.0187 | 0.1968 | 0.2920 | 0.3149 |
| Lung3 - lungC3 | 0.4423 | 0.0178 | 0.3940 | 0.4880 | 0.7325 |
| mg – mgC1 | 1.4559 | 0.0169 | 1.4112 | 1.4995 | 0.8649 |
| mg – mgC2 | 1.5092 | 0.0167 | 1.4674 | 1.5515 | 0.9801 |
| mg – mgC3 | 1.5781 | 0.0169 | 1.5325 | 1.6230 | 0.5572 |
| mg – mgC4 | 1.7216 | 0.0161 | 1.6827 | 1.7641 | 0.4184 |
| mg – mgC5 | 1.6891 | 0.0160 | 1.6491 | 1.7288 | 0.8315 |
| mg – mgC6 | 1.5171 | 0.0163 | 1.4752 | 1.5597 | 0.5534 |
| mg – mgC7 | 1.6033 | 0.0158 | 1.5621 | 1.6467 | 0.0835 |
| mg – mgC8 | 1.4414 | 0.0158 | 1.4007 | 1.4827 | 0.8337 |
| skin1 - skinC | 0.4569 | 0.0146 | 0.4190 | 0.4938 | 0.7170 |
| skin2 - skinC | -0.3502 | 0.0107 | -0.3782 | -0.3219 | 0.8708 |
| skin3 - skinC | 0.5479 | 0.0128 | 0.5150 | 0.5816 | 0.9169 |
| spleen1 - spleenC | 0.3777 | 0.0132 | 0.3415 | 0.4119 | 0.1212 |
| spleen2 - spleenC | 0.9128 | 0.0130 | 0.8816 | 0.9483 | 0.1097 |

S(Difference) – Standard deviation of the difference; Lower and Upper Limits (LL and UL) are approximate 95% limits for the true difference obtained by the Bootstrap Percentile Interval method.
Shapiro P – Probability of the Shapiro-Wilks test of normality for the differences.
