# Supplementary material for: Cancer Reduces Transcriptome Specialization
Source: PLoS One. 2010 May 3;5(5):e10398. doi: 10.1371/journal.pone.0010398 (PMC2862708; doi:10.1371/journal.pone.0010398)
Supplement: Table S8 — Approximate 99% Confidence interval for the differences in specialization between normal and tumor tissues in chromosomes in the analysis of dataset C (Human Transcriptome Map). (0.02 MB PDF) [file pone.0010398.s022.pdf]

chromosome	Difference	S Difference	LL	UL	Shapiro P
All	0.006825	0.000070	0.006643	0.007017	0.149055
1	0.007803	0.000236	0.007213	0.008396	0.506395
2	0.005530	0.000226	0.004922	0.006068	0.005744
3	0.006404	0.000252	0.005731	0.007039	0.148218
4	0.013406	0.000593	0.011945	0.014919	0.738149
5	0.002073	0.000319	0.001250	0.002900	0.618355
6	0.001794	0.000286	0.001063	0.002541	0.757439
7	0.002217	0.000262	0.001536	0.002882	0.938966
8	0.005271	0.000382	0.004223	0.006273	0.334166
9	0.002357	0.000261	0.001689	0.002973	0.766277
10	0.009588	0.000376	0.008644	0.010575	0.996131
11	0.009959	0.000315	0.009174	0.010755	0.524240
12	0.008030	0.000244	0.007391	0.008661	0.865837
13	0.002095	0.000385	0.001142	0.003122	0.451651
14	0.006392	0.000370	0.005400	0.007329	0.436918
15	0.001887	0.000264	0.001242	0.002581	0.983861
16	0.009591	0.000361	0.008610	0.010496	0.436337
17	0.008728	0.000249	0.008085	0.009323	0.563266
18	-0.003124	0.000448	-0.004254	-0.001985	0.969628
19	0.018367	0.000293	0.017654	0.019147	0.070569
20	0.005117	0.000380	0.004162	0.006095	0.388010
21	0.007478	0.000589	0.005848	0.008909	0.050179
22	0.011913	0.000518	0.010582	0.013231	0.813541
X	0.001045	0.000269	0.000320	0.001748	0.899547
Y	-0.000325	0.000594	-0.001821	0.001125	0.538364

LL and UL are the lower and upper 99% confidence levels for the value of the difference in transcriptome specialization (Difference)
