# Supplementary material for: Cancer Reduces Transcriptome Specialization
Source: PLoS One. 2010 May 3;5(5):e10398. doi: 10.1371/journal.pone.0010398 (PMC2862708; doi:10.1371/journal.pone.0010398)
Supplement: Table S10 — Examples of genes exclusively expressed at relatively high rate in tumor tissues in the analysis of dataset C (HTM). (0.02 MB PDF) [file pone.0010398.s024.pdf]

**Locus Id**	**Description**	**Chr.**	***p_i* in tumor**
218449_s1	carboxypeptidase B1 (tissue)	3	1.09E-05
58538_s1	glutamate receptor, metabotropic 5	11	2.66E-06
2029_s1	chromosome 1 open reading frame 158	1	1.15E-06
339089_s1	testis-specific transcript, Y-linked 20	Y	9.99E-07
4439_s1	chromosome 1 open reading frame 63	1	8.97E-07
291838_s1	neuronal cell adhesion molecule	7	8.71E-07
250289_s1	EST	5	7.69E-07
36977_s1	phytanoyl-CoA 2-hydroxylase interacting protein-like	10	6.92E-07
236349_s6	phosphodiesterase 5A, cGMP-specific	4	6.41E-07
281241_s1	Unknown	7	5.64E-07
102029_s1	transmembrane protein 85	15	5.64E-07
121235_s1	EST	16	5.64E-07
201899_s1	EST	3	5.12E-07
31758_s1	ATP synthase, H+ transporting, mitochondrial F1 complex, gamma polypeptide 1	10	5.12E-07
235206_s1	dickkopf homolog 2 (Xenopus laevis)	4	4.87E-07
254365_s1	Unknown	5	4.87E-07
267661_s1	runt-related transcription factor 2	6	4.87E-07
309232_s1	trichorhinophalangeal syndrome I	8	4.87E-07
130734_s5	Non available	17	4.87E-07
182452_s1	chromosome 20 open reading frame 141	20	4.87E-07
336523_s1	Unknown	X	4.87E-07
267362_s2	zinc finger protein 318	6	4.36E-07
271737_s1	KIAA0776	6	4.36E-07
310078_s1	EST	8	4.36E-07
80809_s1	LATS, large tumor suppressor, homolog 2 (Drosophila)	13	4.36E-07
110778_s1	IQ motif containing GTPase activating protein 1	15	4.36E-07
330033_s1	sex comb on midleg-like 1 (Drosophila)	X	4.36E-07
