# Supplementary material for: Cancer Reduces Transcriptome Specialization
Source: PLoS One. 2010 May 3;5(5):e10398. doi: 10.1371/journal.pone.0010398 (PMC2862708; doi:10.1371/journal.pone.0010398)
Supplement: Table S11 — Statistical analyses of genes over expressed in normal and cancer tissues in the human dataset A with regard to their specificity and differences in frequency of expression. (0.08 MB PDF) [file pone.0010398.s025.pdf]

Organ	Specialization			Over-expressed genes				Average S_i in over-expressed genes by category				Average differences in over-expressed genes by category			
	N	C	Diff.	Normal (N)		Cancer (C)									
	δ_j	δ_k	δ_j- δ_k	*N*	%	*c*	%	$\bar{S}_r$	$\bar{S}_v$	t	P-value	$\bar{d}_r$	$\bar{d}_v$	t	P-value
Liver	2.84	1.33	1.52	2,071	7.37	5,520	19.65	**2.24**	**1.80**	14.83	0.000000	0.000425	0.000159	3.58	0.000355
Testis	2.32	1.28	1.04	5,411	19.27	5,840	20.79	**2.74**	**2.04**	30.02	0.000000	0.000130	0.000120	1.33	0.182986
Placenta	2.20	1.28	0.92	6,476	23.06	5,449	19.40	**2.59**	**2.00**	27.53	0.000000	0.000111	0.000133	-2.69	0.007151
Bone	2.16	1.28	0.89	2,632	9.37	3,765	13.40	**2.48**	**1.74**	23.92	0.000000	0.000309	0.000216	5.80	0.000000
Muscle	2.17	1.34	0.83	2,688	9.57	4,001	14.25	**2.11**	**1.80**	11.16	0.000000	0.000327	0.000220	4.62	0.000004
Prostate	1.98	1.35	0.63	2,774	9.88	7,023	25.00	**2.18**	**2.05**	4.82	0.000001	0.000263	0.000104	5.36	0.000000
Lung	1.85	1.25	0.61	4,145	14.76	7,663	27.28	**2.30**	**2.20**	4.07	0.000048	0.000187	0.000101	4.84	0.000001
Kidney	1.91	1.40	0.51	3,106	11.06	6,996	24.91	**2.19**	**2.12**	2.45	0.014472	0.000238	0.000106	11.36	0.000000
Skin	1.82	1.38	0.45	3,192	11.36	10,053	35.79	**2.01**	**2.44**	-18.83	0.000000	0.000202	0.000064	14.22	0.000000
Lymph	2.08	1.69	0.39	2,778	9.89	3,831	13.64	**2.26**	**2.11**	5.07	0.000000	0.000304	0.000221	5.70	0.000000
Lymphr	1.36	1.26	0.10	2,329	8.29	5,102	18.16	**1.78**	**1.90**	-4.86	0.000001	0.000221	0.000101	5.11	0.000000
Eye	1.29	1.35	-0.06	2,705	9.63	6,524	23.23	**1.74**	**2.11**	-16.80	0.000000	0.000215	0.000089	10.72	0.000000
**Average**	**2.00**	**1.35**	**0.65**	**3,359**	**11.96**	**5981**	**21.29**	**2.22**	**2.03**	**NA**	**NA**	**0.000244**	**0.000136**	**NA**	**NA**

N – Normal, C – Cancer. t – Value of the t statistic for the corresponding hypothesis test. See Materials and Methods for the meaning of the column headers. In red cases where the average specialization of over-expressed genes in normal tissues is larger than the corresponding average specialization in cancer; in blue the cases where the opposite is true.
