# Supplementary material for: Cancer Reduces Transcriptome Specialization
Source: PLoS One. 2010 May 3;5(5):e10398. doi: 10.1371/journal.pone.0010398 (PMC2862708; doi:10.1371/journal.pone.0010398)
Supplement: Table S12 — The ten genes with largest influence (See Eq. 8) in the change of specialization of cancerous tissues per organ. Dataset A (human dataset). (0.14 MB PDF) [file pone.0010398.s026.pdf]

Organ	p_{ij}-p_{ik}	Si	ι_i	Gen symbol	Description
Bone	0.0183	3.7219	0.0682	*COL1A1*	Collagen, type I, alpha 1
	0.0233	2.4433	0.0570	*PREP*	CD74 molecule, major histocompatibility complex, class II invariant chain
	0.0126	2.0840	0.0263	*IGHV3-23*	Immunoglobulin heavy constant mu
	0.0099	2.2386	0.0223	*IGLV3-21*	Immunoglobulin lambda joining 3
	0.0092	1.8924	0.0175	*PMS1*	Secreted protein, acidic, cysteine-rich (osteonectin)
	0.0056	2.2882	0.0129	*FN1*	Fibronectin 1
	0.0034	3.4897	0.0120	*LYZ*	Lysozyme (renal amyloidosis)
	0.0045	2.6026	0.0116	*COL1A2*	Collagen, type I, alpha 2
	0.0031	3.5005	0.0107	*CXCL12*	Chemokine (C-X-C motif) ligand 12 (stromal cell-derived factor 1)
	0.0019	**4.5850**	0.0088	*CHAD*	Chondroadherin
Eye	-0.0406	2.3404	0.0950	*ATP5A1*	ATP synthase, H+ transporting, mitochondrial F1 complex, alpha subunit 1, cardiac muscle
	0.0089	2.3814	0.0212	*KRT19*	Keratin 19
	0.0066	1.7633	0.0116	*KRT18*	Keratin 18
	0.0053	1.8446	0.0097	*MGAT4B*	Mannosyl (alpha-1,3-)-glycoprotein beta-1,4-N-acetylglucosaminyltransferase, isozyme B
	-0.0142	0.6431	0.0091	*LOC286184*	Eukaryotic translation elongation factor 1 alpha 1
	0.0092	0.8740	0.0080	*ACTG1*	Actin, gamma 1
	-0.0018	4.4037	0.0080	*AIPL1*	Aryl hydrocarbon receptor interacting protein-like 1
	-0.0024	3.2574	0.0078	*CRABP2*	Cellular retinoic acid binding protein 2
	0.0122	0.6179	0.0076	*IQCE*	Actin, beta
	-0.0016	4.4709	0.0073	*GNB3*	Guanine nucleotide binding protein (G protein), beta polypeptide 3
Kidney	0.0149	3.0899	0.0461	*SPP1*	Secreted phosphoprotein 1 (osteopontin, bone sialoprotein I, early T-lymphocyte activation 1)
	0.0193	2.0606	0.0399	*RAB8A*	Metastasis associated lung adenocarcinoma transcript 1 (non-protein coding)
	-0.0087	2.4195	0.0210	*DHDDS*	Transforming growth factor, beta-induced, 68kDa
	0.0048	4.1757	0.0199	*BHMT*	Betaine-homocysteine methyltransferase
	0.0061	3.1837	0.0194	*LOC145663*	Glycine amidinotransferase (L-arginine:glycine amidinotransferase)
	0.0068	2.4985	0.0170	*SUCLG1*	Succinate-CoA ligase, alpha subunit
	0.0036	**4.5850**	0.0164	*UMOD*	Uromodulin (uromucoid, Tamm-Horsfall glycoprotein)
	0.0038	4.2281	0.0161	*TMEM27*	Transmembrane protein 27
	0.0086	1.4464	0.0124	*EIF3A*	Peroxiredoxin 3
	0.0059	2.0289	0.0119	*LOC100129792*	Selenoprotein P, plasma, 1
Liver	0.1092	4.0869	0.4464	*ALB*	Albumin
	0.0806	4.0863	0.3292	*FGG*	Fibrinogen gamma chain
	0.0367	3.4788	0.1275	*SERPINA1*	Serpin peptidase inhibitor, clade A (alpha-1 antiproteinase, antitrypsin), member 1
	0.0293	3.7364	0.1093	*ORM2*	Orosomucoid 1
	0.0249	4.1199	0.1026	*SAA1*	Serum amyloid A2
	0.0195	4.0086	0.0781	*MARVELD3*	Haptoglobin
	0.0164	3.9271	0.0644	*APOH*	Apolipoprotein H (beta-2-glycoprotein I)
	0.0153	4.1105	0.0630	*RLBP1*	Serum amyloid A2
	0.0123	4.0724	0.0503	*APCS*	Amyloid P component, serum
	0.0095	4.3144	0.0409	*ECHS1*	C-reactive protein, pentraxin-related
Lung	0.0581	2.0606	0.1196	*RAB8A*	Metastasis associated lung adenocarcinoma transcript 1 (non-protein coding)
	0.0152	4.5500	0.0690	*SFTPC*	Surfactant, pulmonary-associated protein C
	0.0191	1.4591	0.0279	*B2M*	Beta-2-microglobulin

	0.0229	1.1466	0.0262	*FTL*	Ferritin, light polypeptide
	0.0103	2.3148	0.0239	*SAT1*	Spermidine/spermine N1-acetyltransferase 1
	0.0058	3.0551	0.0178	*IGJ*	Immunoglobulin J polypeptide, linker protein for immunoglobulin alpha and mu polypeptides
	0.0075	2.3447	0.0176	*LOC100132330*	Netrin 2-like (chicken)
	-0.0168	0.7566	0.0127	*LOC643281*	Glyceraldehyde-3-phosphate dehydrogenase
	0.0034	3.4788	0.0119	*SERPINA1*	Serpin peptidase inhibitor, clade A (alpha-1 antiproteinase, antitrypsin), member 1
	0.0058	1.9756	0.0115	*HLA-DRB5*	Major histocompatibility complex, class II, DR beta 3
Lymph	0.0108	4.0121	0.0435	*PNO1*	Inositol polyphosphate-5-phosphatase, 145kDa
	-0.0156	2.4433	0.0380	*PREP*	CD74 molecule, major histocompatibility complex, class II invariant chain
	0.0097	3.4892	0.0338	*KIAA0746*	KIAA0746 protein
	0.0085	3.3704	0.0287	*SBF1*	SET binding factor 1
	-0.0110	2.5251	0.0277	*HLA-DRA*	Major histocompatibility complex, class II, DR alpha
	0.0055	4.1578	0.0228	*INPP5D*	Inositol polyphosphate-5-phosphatase, 145kDa
	0.0051	4.3151	0.0219	*DENND3*	DENN/MADD domain containing 3
	0.0058	3.1221	0.0180	*DNMT1*	DNA (cytosine-5-)-methyltransferase 1
	0.0044	3.6351	0.0158	*FAM53B*	Family with sequence similarity 53, member B
	-0.0104	1.4591	0.0152	*B2M*	Beta-2-microglobulin
Lymphr	0.0393	2.4433	0.0959	*PREP*	CD74 molecule, major histocompatibility complex, class II invariant chain
	0.0117	2.0840	0.0244	*IGHV3-23*	Immunoglobulin heavy constant mu
	0.0088	2.3447	0.0206	*LOC100132330*	Netrin 2-like (chicken)
	-0.0273	0.6431	0.0175	*LOC286184*	Eukaryotic translation elongation factor 1 alpha 1
	0.0133	1.1925	0.0159	*RPL23A*	Ribosomal protein L23a
	0.0166	0.8740	0.0145	*ACTG1*	Actin, gamma 1
	0.0146	0.8944	0.0131	*RPS3A*	Ribosomal protein S3A
	0.0040	2.6771	0.0107	*CORO1A*	Coronin, actin binding protein, 1A
	0.0039	2.2386	0.0088	*IGLV3-21*	Immunoglobulin lambda joining 3
	0.0069	1.2472	0.0086	*RPL10A*	Ribosomal protein L10a
Muscle	0.0221	4.0996	0.0907	*MYL1*	Myosin, light chain 1, alkali; skeletal, fast
	0.0144	4.2225	0.0610	*LOC100131039*	Titin
	-0.0183	2.7967	0.0510	*LOC100131951*	Serine hydroxymethyltransferase 2 (mitochondrial)
	0.0106	4.1878	0.0445	*MYOT*	Myotilin
	0.0099	4.3945	0.0434	*TRDN*	Triadin
	0.0091	4.3722	0.0399	*XIRP2*	Xin actin-binding repeat containing 2
	0.0147	2.6333	0.0386	*MRCL3*	Myosin regulatory light chain MRCL3
	0.0180	2.0606	0.0372	*RAB8A*	Metastasis associated lung adenocarcinoma transcript 1 (non-protein coding)
	0.0172	2.0907	0.0359	*LOC100132526*	Reticulon 4
	0.0087	4.1005	0.0357	*PLN*	Phospholamban
Placenta	0.0221	4.5066	0.0994	*CGA*	Glycoprotein hormones, alpha polypeptide
	-0.0121	3.8925	0.0470	*H19*	H19, imprinted maternally expressed transcript (non-protein coding)
	0.0108	3.9718	0.0429	*TFPI2*	Tissue factor pathway inhibitor 2
	0.0061	**4.5850**	0.0278	*CYP19A1*	Cytochrome P450, family 19, subfamily A, polypeptide 1
	0.0059	4.5449	0.0267	*CSH2*	Chorionic somatomammotropin hormone 2
	0.0052	**4.5850**	0.0239	*CSH1*	Chorionic somatomammotropin hormone 1 (placental lactogen)
	0.0051	4.4117	0.0226	*DIPAS*	Pregnancy-associated plasma protein A, pappalysin 1
	0.0077	2.6699	0.0206	*GDF15*	Growth differentiation factor 15
	0.0045	4.5534	0.0204	*PSG1*	Pregnancy specific beta-1-glycoprotein 1

	0.0040	4.2553	0.0172	*KISS1*	KiSS-1 metastasis-suppressor
Prostate	0.0357	4.1193	0.1469	*MSMB*	Microseminoprotein, beta-
	0.0664	2.0606	0.1369	*RAB8A*	Metastasis associated lung adenocarcinoma transcript 1 (non-protein coding)
	0.0196	4.2511	0.0834	*SEMG1*	Semenogelin I
	0.0175	4.2957	0.0751	*ACPP*	Acid phosphatase, prostate
	0.0062	4.1368	0.0258	*SEMG2*	Semenogelin II
	0.0063	3.6639	0.0232	*KLK3*	Kallikrein-related peptidase 3
	0.0114	1.4591	0.0166	*B2M*	Beta-2-microglobulin
	0.0034	4.2611	0.0146	*TGM4*	Transglutaminase 4 (prostate)
	0.0034	3.3597	0.0115	*ACTG2*	Actin, gamma 2, smooth muscle, enteric
	0.0028	3.9572	0.0112	*TRGV9*	T cell receptor gamma constant 2
Skin	0.0188	3.7369	0.0701	*FADS2*	Fatty acid desaturase 2
	0.0073	3.4686	0.0252	*KRT14*	Keratin 14 (epidermolysis bullosa simplex, Dowling-Meara, Koebner)
	0.0065	3.5518	0.0232	*KRT5*	Keratin 5 (epidermolysis bullosa simplex, Dowling-Meara/Kobner/Weber-Cockayne types)
	0.0047	4.5660	0.0215	*KRT79*	Keratin 79
	0.0052	3.9904	0.0206	*KRT10*	Keratin 10 (epidermolytic hyperkeratosis; keratosis palmaris et plantaris)
	0.0044	4.2665	0.0189	*KRT1*	Keratin 1 (epidermolytic hyperkeratosis)
	-0.0044	4.1710	0.0185	*SILV*	Silver homolog (mouse)
	0.0088	2.0226	0.0178	*FASN*	Fatty acid synthase
	0.0046	3.5722	0.0164	*CRAT*	Carnitine acetyltransferase
	0.0052	3.0389	0.0158	*KRT42P*	Keratin 17
Testis	0.0068	**4.5850**	0.0312	*PRM1*	Protamine 1
	0.0123	2.0606	0.0254	*RAB8A*	Metastasis associated lung adenocarcinoma transcript 1 (non-protein coding)
	-0.0215	0.6431	0.0139	*LOC286184*	Eukaryotic translation elongation factor 1 alpha 1
	0.0030	**4.5850**	0.0138	*PRM2*	Protamine 2
	0.0030	**4.5850**	0.0138	*SPATA4*	Spermatogenesis associated 4
	0.0030	**4.5850**	0.0138	*C14orf37*	CDNA FLJ25810 fis, clone TST07303
	0.0024	4.4894	0.0107	*ANKRD7*	
	0.0021	4.4451	0.0095	*SPATA22*	Spermatogenesis associated 22
	0.0030	3.0088	0.0092	*CETN3*	Centrin, EF-hand protein, 3 (CDC31 homolog, yeast)
	-0.0168	0.5180	0.0087	*HNRPA1P5*	Eukaryotic translation elongation factor 1 alpha 1

p_{ij}-p_{ik} = Differential expression frequency in normal (j) and cancerous (k) tissue.
