# Supplementary material for: Cancer Reduces Transcriptome Specialization
Source: PLoS One. 2010 May 3;5(5):e10398. doi: 10.1371/journal.pone.0010398 (PMC2862708; doi:10.1371/journal.pone.0010398)
Supplement: Table S13 — Statistical analyses of genes over expressed in normal and cancer tissues in the mouse dataset (B) with regard to their specificity and differences in frequency of expression. (0.07 MB PDF) [file pone.0010398.s027.pdf]

	Specialization			Over-expressed genes				Average S_i in over-expressed genes by category				Average differences in over-expressed genes by category			
	N	C	Dic.	Normal (N)		Cancer (C)									
**Organ**	**δ_j**	**δ_k**	**δ_j- δ_k**	***N***	**%**	***C***	**%**	$\bar{S}_r$	$\bar{S}_v$	**T**	**P-value**	$\bar{d}_r$ (N)	$\bar{d}_v$ (C)	**t**	**P-value**
Liver	1.90	1.79	0.11	6,769	27	2,102	8	**1.73**	**1.62**	5	0.00000	0.00010	0.00033	-15	0.00000
Luna	1.60	1.29	0.31	5,261	21	8,734	35	**1.88**	**1.80**	5	0.00000	0.00012	0.00007	11	0.00000
MG	2.52	1.37	1.15	1,581	6	15,527	62	**1.72**	**2.04**	-14	0.00000	0.00054	0.00005	3	0.01016
Skin	1.63	1.47	0.15	8,080	32	4,851	19	**1.97**	**1.56**	28	0.00000	0.00008	0.00014	-7	0.00000
Soleen	1.73	1.37	0.36	6,232	25	2,581	10	**1.84**	**1.43**	22	0.00000	0.00014	0.00033	-18	0.00000
**Average**	**1.87**	**1.46**	**0.42**	**5,585**	**22**	**6759**	**27**	**1.83**	**1.69**	**9**	**NA**	**0.00020**	**0.00018**	**NA**	**NA**

N – Data from the normal tissue; C – data from the cancerous tissues. t – Value of the t-statistic and associated P-value. See definitions of other columns headings in Materials and Methods. In red cases where the average specialization of over-expressed genes in normal tissues is larger than the corresponding average specialization in cancer; in blue the cases where the opposite is true.
