# Supplementary material for: Cancer Reduces Transcriptome Specialization
Source: PLoS One. 2010 May 3;5(5):e10398. doi: 10.1371/journal.pone.0010398 (PMC2862708; doi:10.1371/journal.pone.0010398)
Supplement: Table S14 — The ten genes with largest influence (See Eq. 8) in the change of specialization of cancerous tissues per organ. Dataset B (mouse dataset). (0.10 MB PDF) [file pone.0010398.s028.pdf]

Organ	p_{ij}-p_{ik}	Si	ι_i	Gen symbol	Description
Liver	-0.0221	2.3518	0.0521	*Alb*	Albumin
	0.0120	**3.3219**	0.0400	*OTTMUSG00000007428*	Novel member of the major urinary protein (Mup) gene family
	0.0117	**3.3219**	0.0389	*OTTMUSG00000007431*	Major urinary protein 1
	0.0219	1.5654	0.0343	*Hbb-b1*	Hemoglobin, beta adult minor chain
	0.0092	**3.3219**	0.0306	*100039247*	Major urinary protein 2
	0.0118	2.5685	0.0303	Not Available	Serine (or cysteine) peptidase inhibitor, clade A, member 1b
	0.0087	3.2763	0.0286	Not Available	Cytochrome P450, family 2, subfamily e, polypeptide 1
	0.0114	1.9310	0.0221	*Apoe*	Apolipoprotein E
	0.0063	3.2983	0.0209	*OTTMUSG00000015595*	Major urinary protein 1
	0.0076	2.7411	0.0209	*LOC545679*	Ferritin light chain 1
Lung	0.0172	3.3124	0.0571	*Scgb1a1*	Secretoglobin, family 1A, member 1 (uteroglobin)
	0.0060	1.5654	0.0094	*Hbb-b1*	Hemoglobin, beta adult minor chain
	-0.0029	2.4190	0.0070	*NA*	Insulin-like growth factor binding protein 5
	-0.0043	1.5251	0.0066	*NA*	CDNA clone IMAGE:40049146
	0.0018	**3.3219**	0.0061	*Sftpa1*	Surfactant associated protein A1
	0.0016	**3.3219**	0.0054	*Cxcl15*	Chemokine (C-X-C motif) ligand 15
	0.0023	2.3005	0.0052	*Slc34a2*	Solute carrier family 34 (sodium phosphate), member 2
	-0.0025	1.9212	0.0047	*Spp1*	Secreted phosphoprotein 1
	0.0013	**3.3219**	0.0044	*Sftpc*	Surfactant associated protein C
	-0.0023	1.9063	0.0043	*NA*	Casein alpha s1
MG	0.2472	3.0653	0.7577	*Csn2*	Casein beta
	0.1382	3.3184	0.4586	*Csn1s2b*	Casein alpha s2-like B
	0.0735	3.0184	0.2220	*Csn3*	Casein kappa
	0.0491	**3.3219**	0.1632	*NA*	Transcribed locus
	0.0256	3.2646	0.0837	*NA*	Transcribed locus
	0.0140	2.9447	0.0412	*Wap*	Whey acidic protein
	0.0075	3.1801	0.0238	*Csn1s1*	Similar to XP_001845517.1 calmodulin-binding transcription activator
	-0.0074	2.7876	0.0206	*Ltf*	Lactotransferrin
	0.0063	3.2078	0.0203	*NA*	Transcribed locus, moderately similar to NP_598738.1 transferrin
	0.0046	3.2005	0.0148	*Csn1s2a*	Transcribed locus
Skin	0.0382	3.1051	0.1187	*Mmp12*	Matrix metallopeptidase 12
	-0.0140	3.2749	0.0457	*NA*	Tyrosinase-related protein 1
	0.0102	3.1996	0.0326	*Mmp8*	Matrix metallopeptidase 8
	-0.0083	**3.3219**	0.0277	*Tyr*	Tyrosinase
	-0.0077	**3.3219**	0.0257	*Mrgpra8*	MAS-related GPR, member B1
	-0.0090	2.7675	0.0249	*Mgll*	Monoglyceride lipase
	0.0108	2.2101	0.0239	*Txnip*	Thioredoxin interacting protein
	0.0093	2.4959	0.0232	*Laptm5*	Lysosomal-associated protein transmembrane 5
	-0.0075	2.9747	0.0224	*Ednrb*	Endothelin receptor type B
	-0.0066	3.1846	0.0210	*Trpm1*	Transient receptor potential cation channel, subfamily M, member 1
Soleen	0.0202	3.2325	0.0653	*Slc4a1*	Solute carrier family 4 (anion exchanger), member 1
	0.0117	3.0433	0.0355	*Tgtp*	T-cell specific GTPase
	0.0100	2.8021	0.0279	*Cd69*	CD69 antigen
	0.0077	**3.3219**	0.0256	*Ms4a1*	Membrane-spanning 4-domains, subfamily A, member 1
	0.0093	2.7163	0.0252	*Tmcc2*	Transmembrane and coiled-coil domains 2
	0.0068	3.2680	0.0221	*NA*	Chemokine (C-X-C motif) ligand 10
	0.0068	2.9154	0.0199	*Cd83*	CD83 antigen

	0.0083	2.2246	0.0185	*NA*	Eukaryotic translation initiation factor 4A, isoform 3
	0.0046	2.6918	0.0125	*Tnfaip3*	Tumor necrosis factor, alpha-induced protein 3
	-0.0068	1.4098	0.0096	*Rpl8*	Ribosomal protein L8
