# Supplementary material for: Cancer Reduces Transcriptome Specialization
Source: PLoS One. 2010 May 3;5(5):e10398. doi: 10.1371/journal.pone.0010398 (PMC2862708; doi:10.1371/journal.pone.0010398)
Supplement: Table S15 — Statistical analyses of loci over expressed in normal and tumor tissues in Chromosome 18 of dataset (C) with regard to their specificity and differences in frequency of expression. (0.06 MB PDF) [file pone.0010398.s029.pdf]

Specialization			Over-expressed loci				Average S_i in over-expressed loci by category				Average differences in over-expressed loci by category			
N	C	Dic.	Normal (N)		Tumor (C)									
δ_j	δ_k	$\delta_j - \delta_k$	*N*	%	*C*	%	$\bar{S}_r$	$\bar{S}_v$	t	P-value	$\bar{d}_r$ (N)	$\bar{d}_v$ (C)	t	P-value
0.009	0.012	-0.003	499	47	574	53	**0.1123**	**0.2019**	-5.3	1.7e-07	0.00016	0.00014	0.44	0.6598
