# Supplementary material for: Cancer Reduces Transcriptome Specialization
Source: PLoS One. 2010 May 3;5(5):e10398. doi: 10.1371/journal.pone.0010398 (PMC2862708; doi:10.1371/journal.pone.0010398)
Supplement: Table S16 — The ten most influential loci in the increase of specialization of tumors in Chromosome 18 (dataset C). (0.05 MB PDF) [file pone.0010398.s030.pdf]

Locus (TU)	Description	Si	p_{ij}-p_{ik}	ι_i
139121_s1	EST (unknown function)	0.9694	-3.7135E-05	3.5999E-05
140035_s1	Aquaporin 4 (AQP4)	0.3722	-4.2907E-05	1.5971E-05
141439_s1	solute carrier family 14 (urea transporter), member 1 (Kidd blood group) (SLC14A1)	0.2997	-8.0686E-06	2.4178E-06
140578_s1	Dystrobrevin, alpha (DTNA)	0.1336	-6.3726E-06	8.5122E-07
142131_s1	mitogen-activated protein kinase 4 (MAPK4)	0.1659	-5.0963E-06	8.4557E-07
138752_s1	similar to KIAA1074 protein (unknown function)	0.0109	-6.2283E-05	6.8135E-07
142311_s1	EST (unknown function)	0.4562	-1.4053E-06	6.4111E-07
143182_s1	phosphatidylinositol glycan anchor biosynthesis, class N	0.5679	-1.087E-06	6.1726E-07
138444_s1	RAB31, member RAS oncogene family	0.0801	-7.5669E-06	6.0617E-07
139157_s1	ankyrin repeat domain 30B (unknown function)	0.0097	-5.627E-05	5.436E-07
TU – Locus identifier.				
