# Supplementary material for: Cancer Reduces Transcriptome Specialization
Source: PLoS One. 2010 May 3;5(5):e10398. doi: 10.1371/journal.pone.0010398 (PMC2862708; doi:10.1371/journal.pone.0010398)
Supplement: Table S17 — Classifications by Gene Ontology of the genes presented in Table 1. (0.05 MB PDF) [file pone.0010398.s031.pdf]

Table S17
Classifications by Gene Ontology of the genes presented in Table 1

Gene	Biological process	Molecular function	Cellular component
SILV	Melanin biosynthetic process from tyrosine	Not available	Extracellular region
DHRS2	Oxidation reduction	Binding	Nucleus
SOX10	Regulation of transcription, DNA-dependent	DNA binding	Nucleus
TRAF7	Activation of MAPKKK activity	Ubiquitin-protein ligase activity	Ubiquitin ligase complex
C10orf2	Not available	Not available	Not available
PRPS1	Nucleoside metabolic process	Magnesium ion binding	Cytosol
MLANA	Not available	Not available	Integral to plasma membrane
AIPL1	Retina homeostasis	Farnesylated protein binding	Photoreceptor inner segment
MAGEA6	Not available	Not available	Not available
KLHL21	Ubiquitin-dependent protein catabolic process	Protein binding	Not available
GNB3	Signal transduction	GTPase activity	Nucleus
KIFC1	Mitotic sister chromatid segregation	Nucleotide binding	Nucleus
S100B	Axonogenesis	Calcium ion binding	Ruffle
CDT1	DNA replication checkpoint	DNA binding	Nucleus
ZWINT	Mitotic sister chromatid segregation	Protein N-terminus binding	Condensed chromosome kinetochore
XAB2	Blastocyst development	Protein binding	Intracellular
SLC45A2	Melanin biosynthetic process from tyrosine	Not available	Membrane
