# Supplementary material for: Cancer Reduces Transcriptome Specialization
Source: PLoS One. 2010 May 3;5(5):e10398. doi: 10.1371/journal.pone.0010398 (PMC2862708; doi:10.1371/journal.pone.0010398)
Supplement: Table S18 — Classifications by Gene Ontology for the genes presented in Table 2. (0.05 MB PDF) [file pone.0010398.s032.pdf]

Table S18
Classifications by Gene Ontology for the genes presented in Table 2

Organ	Gene	Biological process	Molecular function	Cellular component
Eye	OTX2	Cell fate specification	Transcription factor activity	Nucleus
Liver	ASGR2	Endocytosis	Binding	Membrane
Lung	T	Vasculogenesis	Transcription factor activity	Nucleus
Lymph	C4orf7	Not available	Not available	Not available
Limphr	IL9R	Signal transduction	Receptor activity	Membrane
Placenta	DNMT3L	In utero embryonic development	Enzyme activator activity	Condensed nuclear chromosome
Skin	MLANA	Not available	Not available	Integral to plasma membrane
